# Supplementary material for: Assessment of breath volatile organic compounds in acute cardiorespiratory breathlessness: a protocol describing a prospective real-world observational study
Source: BMJ Open. 2019 Mar 8;9(3):e025486. doi: 10.1136/bmjopen-2018-025486 (PMC6429860; doi:10.1136/bmjopen-2018-025486)
Supplement: Author's manuscript [file bmjopen-2018-025486.draft_revisions.pdf]

BMJ Open

BMJ Open is committed to open peer review. As part of this commitment we make the peer review history of every article we publish publicly available.

When an article is published we post the peer reviewers' comments and the authors' responses online. We also post the versions of the paper that were used during peer review. These are the versions that the peer review comments apply to.

The versions of the paper that follow are the versions that were submitted during the peer review process. They are not the versions of record or the final published versions. They should not be cited or distributed as the published version of this manuscript.

BMJ Open is an open access journal and the full, final, typeset and author-corrected version of record of the manuscript is available on our site with no access controls, subscription charges or pay-per-view fees (http://bmjopen.bmj.com).

If you have any questions on BMJ Open's open peer review process please email info.bmjopen@bmj.com

BMJ Open

Assessment of Breath Volatile Organic Compounds in Acute Cardio-respiratory Breathlessness: A Prospective Real World Observational Study

Journal:	*BMJ Open*
Manuscript ID	bmjopen-2018-025486
Article Type:	Protocol
Date Submitted by the Author:	17-Jul-2018

Complete List of Authors:	Ibrahim, Wadah; Leicester NIHR Biomedical Research Centre (Respiratory theme); College of Life Sciences, Department of Infection, Immunity and Inflammation, University of Leicester<br>Wilde, Michael; University of Leicester, Department of Chemistry<br>Cordell, Rebecca; University of Leicester, Department of Chemistry<br>Salman, Dahlia; Loughborough University, Department of Chemistry<br>Ruszkiewicz, Dorota; Loughborough University, Department of Chemistry<br>Bryant, Luke; University of Leicester, Department of Chemistry<br>Richardson, Matthew; Leicester NIHR Biomedical Research Centre (Respiratory theme); University of Leicester, Infection, Inflammation and Immunity<br>Free, Robert; Leicester NIHR Biomedical Research Centre (Respiratory theme); University of Leicester, Infection, Inflammation and Immunity<br>Zhao, Bo; Leicester NIHR Biomedical Research Centre (Respiratory theme); University of Leicester, Infection, Inflammation and Immunity<br>Yousuf, Ahmed; University of Leicester, Infection, Inflammation and Immunity; Leicester NIHR Biomedical Research Centre (Respiratory theme)<br>White, Christobelle; University of Leicester, Infection, Inflammation and Immunity; Leicester NIHR Biomedical Research Centre (Respiratory theme)<br>Russell, Richard; University of Leicester, Infection, Inflammation and Immunity; Leicester NIHR Biomedical Research Centre (Respiratory theme)<br>Jones, Sheila; University Hospitals of Leicester NHS Trust<br>Patel, Bharti; University Hospitals of Leicester NHS Trust<br>Awal, Asia; University Hospitals of Leicester NHS Trust<br>Phillips, Rachael; NIHR Leicester Clinical Research Facility<br>Fowkes, Graham; NIHR Leicester Clinical Research Facility<br>McNally, Teresa; Paediatric Clinical Investigation Centre<br>Foxon, Clare; Paediatric Clinical Investigation Centre<br>Bhatt, Hetan; Leicester NIHR Biomedical Research Centre (Respiratory theme)<br>Peltrini, Rosa; Leicester NIHR Biomedical Research Centre (Respiratory theme); University of Leicester, Department Infection, Inflammation and Immunity<br>Singapuri, Amisha; Leicester NIHR Biomedical Research Centre (Respiratory theme)<br>Hargadon, Beverley; Leicester NIHR Biomedical Research Centre (Respiratory theme); University of Leicester, Infection, Inflammation and Immunity<br>Suzuki, T; Department of Cardiovascular Sciences, University of Leicester, , Cardiovascular Research Centre; Leicester NIHR Biomedical Research

Centre (Cardiovascular theme)
Ng, Leong; Department of Cardiovascular Sciences, University of Leicester, Cardiovascular Research Centre; Leicester NIHR Biomedical Research Centre (Cardiovascular theme)
Gaillard, Erol; Leicester NIHR Biomedical Research Centre (Respiratory theme); Paediatric Clinical Investigation Centre
Beardsmore, Caroline; Leicester NIHR Biomedical Research Centre (Respiratory theme); Paediatric Clinical Investigation Centre
Ryanna, Kimuli; University Hospitals of Leicester NHS Trust
Pandya, Hitesh; Discovery Medicine, Respiratory Therapeutic Area, GlaxoSmithKline PLC
Coates, Tim; University of Leicester, Infection, Inflammation and Immunity; Department of Cardiovascular Sciences, University of Leicester
Monks, Paul; University of Leicester, Infection, Inflammation and Immunity; University of Leicester, Department of Chemistry
Greening, Neil; Leicester NIHR Biomedical Research Centre (Respiratory theme); University of Leicester, Infection, Inflammation and Immunity
Brightling, Christopher; Leicester NIHR Biomedical Research Centre (Respiratory theme); University of Leicester, Infection, Inflammation and Immunity
Thomas, Paul; Loughborough University, Department of Chemistry
Siddiqui, Salman; Leicester NIHR Biomedical Research Centre (Respiratory theme); University of Leicester, Infection, Inflammation and Immunity

Keywords: Breathlessness, Observational study, Volatile Organic Compound, Breath analysis

SCHOLARONE™ Manuscripts

For peer review only

Assessment of Breath Volatile Organic Compounds in Acute Cardio-respiratory Breathlessness: A Prospective Real World Observational Study

Wadah Ibrahim[1,2], Michael Wilde[3], Rebecca Cordell[3], Dahlia Salman[4], Dorota Ruszkiewicz[4], Luke Bryant[3], Matthew Richardson[1,2], Robert C. Free[1,2], Bo Zhao[1,2], Ahmed Yousuf[1,2], Christobelle White[1,2], Richard J Russell[1,2], Sheila Jones[5], Bharti Patel[5], Asia Awal[5], Rachael Phillips[6], Graham Fowkes[6], Teresa McNally[7], Clare Foxon[7], Hetan Bhatt[1,2], Rosa Peltrini[1,2], Amisha Singapuri[1,2], Beverley Hargadon[1,2], Toru Suzuki[8,9], Leong L. Ng[8,9], Erol A Gaillard[2,7], Caroline Beardsmore[2,7], Kimuli Ryanna[5], Hitesh Pandya[11], Tim Coates[2,10], Paul Monks[2,3], Neil Greening[1,2], Chris E Brightling[1,2], Paul Thomas[4], Salman Siddiqui[1,2]

wadah.ibrahim@leicester.ac.uk; mjw77@leicester.ac.uk; rc145@leicester.ac.uk; d.salman@lboro.ac.uk; d.m.ruszkiewicz2@lboro.ac.uk; lb368@leicester.ac.uk; mr251@leicester.ac.uk; rcf8@leicester.ac.uk; bo.zhao@leicester.ac.uk; ajy7@leicester.ac.uk; cmjw3@leicester.ac.uk; rjr22@leicester.ac.uk; rjr22@leicester.ac.uk; sheila.jones@uhl-tr.nhs.uk; bharti.m.patel@uhl-tr.nhs.uk; asia.awal@uhl-tr.nhs.uk; rachael.phillips@uhl-tr.nhs.uk; graham.fowkes@uhl-tr.nhs.uk; tm23@leicester.ac.uk; clare.foxon@uhl-tr.nhs.uk; hetan.bhatt@uhl-tr.nhs.uk; rp417@leicester.ac.uk; amisha.singapuri@uhl-tr.nhs.uk; beverley.hargadon@uhl-tr.nhs.uk; ts263@le.ac.uk; lln1@le.ac.uk; eag15@leicester.ac.uk; csb@leicester.ac.uk; kimuli.ryanna@uhl-tr.nhs.uk; hitesh.x.pandya@gsk.com; tim.coates@leicester.ac.uk; paul.monks@leicester.ac.uk; neil.greening@leicester.ac.uk; ceb17@leicester.ac.uk; C.L.P.Thomas@lboro.ac.uk; ss338@leicester.ac.uk

Corresponding author: Professor Salman Siddiqui, ss338@le.ac.uk

NIHR Leicester Biomedical Research Centre (Respiratory Theme)

Glenfield Hospital,

Groby Road

Leicester

LE3 9QP

t: +44 116 256 3841

e: ss338@ le.ac.uk

Authors' affiliations:

[1]Leicester NIHR Biomedical Research Centre (Respiratory theme), Glenfield Hospital, Groby Road, Leicester LE3 9QP

[2] College of Life Sciences, Department of Infection, Immunity and Inflammation, University of Leicester, University Road, Leicester LE1 7RH

[3] Department of Chemistry, University of Leicester, University Road, Leicester, LE1 7RH

For peer review only

For peer review only - http://bmjopen.bmj.com/site/about/guidelines.xhtml

[4]Department of Chemistry, Loughborough University, Epinal Way, Loughborough, LE11 3TU

[5] University Hospitals of Leicester NHS Trust, Glenfield Hospital, Groby Road, Leicester LE3 9QP

[6] NIHR Leicester Clinical Research Facility, Research Space, Level 0, Balmoral Building, Leicester Royal Infirmary, Infirmary Square, Leicester, LE1 5WW

[7] Paediatric Clinical Investigation Centre, Leicester Respiratory Biomedical Research Centre, University of Leicester, Leicester Royal Infirmary, Leicester, LE2 7LX

[8] Department of Cardiovascular Sciences, University of Leicester, Cardiovascular Research Centre, Glenfield General Hospital, Leicester LE3 9QP.

[9] Leicester NIHR Biomedical Research Centre (Cardiovascular theme), Glenfield Hospital, Groby Road, Leicester LE3 9QP

[10]Department of Cardiovascular Sciences, University of Leicester, Level G, Jarvis building RMO, Infirmary Square, Leicester LE1 5WW

[11] Discovery Medicine, Respiratory Therapeutic Area, GlaxoSmithKline PLC, Gunnels Wood Road, Stevenage, Hertfordshire, SG1 2NY

For peer review only

Abstract

Introduction

Patients presenting with acute undifferentiated breathlessness are commonly encountered in admissions units across the United Kingdom. Existing blood biomarkers have clinical utility in distinguishing patients with single organ pathologies but have poor discriminatory power in multi factorial presentations. Evaluation of volatile organic compounds (VOC) in exhaled breath offers the potential to develop biomarkers of disease states that underpin acute cardio-respiratory breathlessness, owing to their proximity to the cardio-respiratory system. To date there has been no systematic evaluation of VOC in acute cardio-respiratory breathlessness. The proposed study will seek to use both offline and online VOC technologies to evaluate the predictive value of VOC in identifying common conditions that present with acute cardio-respiratory breathlessness.

Methods and analysis

A prospective real world observational study carried out across three acute admissions units within Leicestershire. Participants with self-reported acute breathlessness, with a confirmed primary diagnosis of either acute heart failure, community acquired pneumonia and acute exacerbation of asthma or COPD will be recruited within 24 hours of admission. Additionally, school age children admitted with severe asthma will be evaluated. All participants will undergo breath sampling on admission and upon recovery following discharge. A range of online technologies including: proton-transfer-reaction mass spectrometry (PTR-MS), gas chromatography ion mobility spectrometry (GC-IMS), atmospheric pressure chemical ionisation- mass spectrometry (APCI-MS) and offline technologies including gas chromatography mass spectroscopy (GC-MS) and comprehensive two-dimensional gas chromatography-mass spectrometry (GCxGC-MS) will be utilised for VOC discovery and replication. For offline technologies a standardised CE marked breath sampling device (ReCIVA®) will be used. All recruited participants will be characterised using existing blood biomarkers including C - reactive protein (CRP), brain derived natriuretic peptide (BNP), Troponin-I and blood eosinophil levels and further evaluated using a range of standardised questionnaires, lung function testing, sputum cell counts and other diagnostic tests pertinent to acute disease.

For peer review only - http://bmjopen.bmj.com/site/about/guidelines.xhtml

Ethics and dissemination

The National Research Ethics Service Committee East Midlands has approved the study protocol (REC number: 16/LO/1747). IRAS 198921. Findings will be presented at academic conferences and published in peer-reviewed scientific journals. Dissemination will be facilitated via a partnership with the East Midlands Academic Health Sciences Network and via interaction with all UK funded MRC/EPSRC molecular pathology nodes.

Key words: Breathlessness; Breath analysis; Volatile Organic Compound, Observational study

For peer review only

For peer review only - http://bmjopen.bmj.com/site/about/guidelines.xhtml

Strengths and Limitations of this Study

Strengths

- A pragmatic real world, prospective, observational study across three admission units that focuses on the systematic discovery and replication of VOC in acutely breathless patients using both online and offline technologies
- The study will evaluate populations that often present with diagnostic uncertainty including elderly multi-morbid patients and school age children
- The proposed study is the largest of its kind in acute disease to characterise VOC with a range of additional assessments that will build a comprehensive phenotype of acute cardio-respiratory exacerbations
- Benchmarking of candidate VOC to established blood based biomarkers utilised in clinical practice e.g. BNP, CRP will form an important method of validation against existing molecular pathology
- The proposed study will build an infrastructure for research and subsequent evaluation of VOC in interventional trials within acute cardio-respiratory exacerbations

Limitations

- Prior acute treatment exposure will need to be accounted for when evaluating potential discriminative biomarkers
- VOC technologies are not currently suited for deployment in patients that are of high clinical acuity e.g. severe respiratory failure and thus the study will self-select patients that can be safely sampled with online and offline methodologies
- Although the study will quantify and evaluate diagnostic uncertainty, the study does not enrich for these cases and the patient population will predominantly comprise of patients in whom a senior clinical decision maker could make the primary clinical diagnosis within 24 hours of admission. As a consequence future studies will be required to evaluate the biomarkers developed in cardio-respiratory exacerbations where the diagnosis is unclear.

For peer review only - http://bmjopen.bmj.com/site/about/guidelines.xhtml

1. Introduction:

Breathlessness is a common symptom of cardio-respiratory illnesses that has a significant direct impact on patients' wellbeing as well as a substantial economic burden on healthcare systems [1]. Although its etiologies can be variable, exacerbations of common complex chronic cardio-respiratory conditions account for approximately 70% of acute presentations with breathlessness, namely exacerbations of asthma and COPD, acute heart failure and community acquired pneumonia [2]. Moreover, moderate and severe breathlessness is significantly associated with all-cause, cardiovascular and COPD mortality[3]. As a consequence symptomatic breathlessness warrants rapid evaluation and targeted diagnostics at presentation.

Diagnostic evaluation of acute breathlessness is heavily reliant on blood based biomarkers e.g. CRP, BNP, Troponin and on occasions blood eosinophil levels. These biomarkers have clinical utility primarily in patients with single pathologies, but have poor discriminatory power in patients with multifactorial presentations of acute breathlessness[4]. There is therefore an unmet need for the development of sensitive and specific biomarkers that differentiate acute breathlessness from its recovery and the common cardio-respiratory conditions that present with acute breathlessness.

CRP plays an important role in diagnosing breathlessness caused by an underlying bacterial pneumonia[5], as well as predicting mortality in patients with COPD [6]. BNP is routinely utilised in acute settings to support the diagnosis of acute heart failure [7].The European Society of Cardiology (ESC) recommends BNP threshold values of <100 pg/mL to rule out acute congestive cardiac failure and values > 500 pg/ml as diagnostic of acute exacerbations of heart failure [8].

The role of peripheral blood eosinophil count in airway inflammation was poorly understood up until the second half of the 19th century when Paul Ehrlich, a German physician and Nobel prize winner, introduced eosin in his technique for white cell differentiation in 1879[9] . Considerable advances in the field of airway inflammation and the role of eosinophils have taken place since [10-12]. More

For peer review only - http://bmjopen.bmj.com/site/about/guidelines.xhtml

recently Bafadhel *et al* suggested that peripheral blood eosinophil count can be used to direct corticosteroid therapy during COPD exacerbations in single centre study [13].

Currently, blood biomarkers together with clinical, physiological and imaging parameters are used in diagnosing the cause of acute breathlessness. Blood biomarkers may be less specific as they originate far from the target organs of interest (the heart and the lungs in cardio-respiratory disease). Sputum, although potentially a more definitive lung specific matrix, is comparatively difficult to obtain particularly in acutely unwell patients, limiting its use in acute disease and highlighting the need for better biomarkers. Ideally these biomarkers would have the following characteristics, (i) they would originate from the target organ of interest, (ii) they would significantly add value to conventional risk scoring and diagnostic algorithms in acute breathlessness, (iii) they would be minimally invasive and suitable for rapid point of care diagnosis in emergency rooms and acute admissions units (iv) they would have diagnostic value in patients with multifactorial acute breathlessness.

Exhaled breath contains thousands of volatile organic compounds (VOC) that reflect biological processes occurring in the host both locally in the airways and systematically offering the potential to develop more effective biomarkers in acutely breathless patients (**Figure 1**).

The proposed program of research will use a combination of offline and online technologies to identify and evaluate the diagnostic and prognostic value of VOC in patients with acute cardio-respiratory related breathlessness (**Figure 2**).

Exhaled breath analysis of volatile chemicals offers the potential to develop more effective biomarkers in acutely breathless patients. The use of breath analysis for disease diagnostics dates back to ancient Greeks where physicians used exhaled breath to diagnose different diseases. Breath odours allow correct associations to certain diseases. For example, the sweet smell of diabetic ketoacidosis, the fishy smell of breath associated to liver illness, the urine-like odour of kidney disease and the smell of the breath of patients with lung abscesses, caused by the proliferation of anaerobic bacteria [14-17].

For peer review only - http://bmjopen.bmj.com/site/about/guidelines.xhtml

More recently, exhaled breath analysis has demonstrated early proof of concept in the diagnosis of acute heart failure, ventilator associated pneumonia[18] and stable state airways disease [15]. The validity of breath analysis has also been demonstrated in breathless children[19]. This population is likely to prefer breath-based tests, as these are minimally invasive. Importantly, a variety of point of care sensors are now available to evaluate potential exhaled breath biomarkers in emergency care settings.

Despite the novelty of non-invasive sampling technology and the growing interest in exhaled breath analysis there remains a disappointing level of comparability across studies due to the lack of standardisation and appropriate data analysis methods.

A recent systemic review by Anders Christiansen *et al* compared eleven publications reporting very heterogeneous designs, methods, patient group sizes, data analytics and, consequently, quite varying results [20].

To our knowledge, no other large studies exploring the use of breath biomarkers in profiling acute breathlessness have been completed. Few studies have explored the use of electronic nose in stable disease with good discriminatory power in COPD [21], Pneumonia [22] and heart failure[23] with relatively small sample size. The focus of the current research study will be to evaluate acutely breathless cardio-respiratory patients using a combination of 'discovery' and near-patient care breath sampling technologies.

Medical Research Council (MRC) and Engineering and Physical Sciences Research Council (EPSRC) have commissioned a series of molecular pathology nodes aimed at developing molecular signatures relevant to disease diagnosis and progression. This was triggered by the clear need for alliance between academic institutions, industry and NHS partners to enhance the benefits of stratified medicine for patients[24, 25].

University of Leicester and Loughborough University were awarded a joint molecular pathology node East Midlands Breathomics Pathology Node (EMBER) which this study forms a key part of.

For peer review only

For peer review only - http://bmjopen.bmj.com/site/about/guidelines.xhtml

2. Methods and Analysis

2.1. Study design

A prospective real world observational study across three acute admissions units within Leicestershire (two adult admissions units and one children's assessment unit). The acute units routinely assess and treat cardio-respiratory admissions due to breathlessness in adults and children.

Participants with self-reported acute breathlessness, either requiring admission or a change in baseline treatment, will be screened for the study. Informed consent will be obtained in all participants following a clinical review by a senior decision maker within 24 hours of acute admission (**Figure 3**).

2.2. Objectives

2.2.1. Primary objective

- To evaluate the sensitivity, specificity, positive and negative predictive value of exhaled breath VOC biomarkers to differentiate acute breathlessness in cardio-respiratory patients.

2.2.2. Secondary objectives:

- To replicate selected breath VOC biomarkers identified in acute breathlessness.
- To discover and replicate breath VOC biomarkers that differentiate the common cardio-respiratory conditions that cause acute breathlessness, specifically (i) acute heart failure, (ii) community acquired pneumonia, (iii) adult exacerbations of asthma and COPD and age-matched adults that do not have cardio-respiratory disease or breathlessness.
- To quantify the level of clinical uncertainty in the primary diagnosis using a 100 mm visual analogue scale and independent clinical adjudication of case notes blinded to the following blood biomarkers (i) CRP (ii) BNP (iii) Troponin-I (iv) blood eosinophils, but not clinical history and acute presentation nor chest x-ray imaging. Potential discriminatory breath VOC biomarkers will be adjusted for clinical uncertainly in statistical models.
- To identify and replicate exhaled breath VOC biomarkers in school age children treated in hospital for severe asthma attacks and compare these to age-matched healthy controls.

For peer review only - http://bmjopen.bmj.com/site/about/guidelines.xhtml

2.2.3. *Exploratory end points (where applicable):*

- To evaluate the dynamic profile of selected breath VOC between the acute state and the recovery state post exacerbation.
- To evaluate the relationship between exhaled VOC biomarkers and clinical outcomes including (i) hospital readmission at 30 and 60 days post event (ii) all-cause mortality over a 2 year period post admission.
- To evaluate the relationship between breath VOC biomarkers and functional measures e.g. physical performance and activity
- To explore potential breath VOC biomarkers of multifactorial acute breathlessness
- To evaluate the relationship between diet, lifestyle and environment upon breath VOC biomarkers

2.3. Sample size estimation

Preliminary data was utilised to conduct sample size estimates from a cohort of acutely breathless patients admitted to acute admissions units over a 6 month period (February 2017 to August 2017). Hundred and twelve adult participants (asthma 46, community acquired pneumonia 26, COPD 22) and eighteen healthy controls were utilised for the analysis.

A panel of ten pre-specified aldehydes, based on literature search [26-28], were extracted from breath using gas chromatography-mass spectrometry (GC-MS). The aldehydes were normalised to a common internal standard and were not background-subtracted.

A closed formula from Hsieh *et al*[29], relating sample size to observable effect size, was used to calculate sample size from logistic regression models of the ten aldehydes with acute breathlessness as the outcome measure. The sample size estimates are also relevant to acute class comparisons vs. the sum of other acute classes.

Based upon the sample size estimates we would have an 80% power, with a type 1 error rate of 5%, to detect an odds ratio of association of 1.2 between two disease classes with 55 patients per class. Given

For peer review only - http://bmjopen.bmj.com/site/about/guidelines.xhtml

For peer review only

the fact that study seeks to discover and replicate breath VOC amongst five adult disease classes (community acquired pneumonia, heart failure, COPD, asthma and healthy aged matched subjects) we would require 110 adult patients per class – 550 patients across the program to achieve these aims.

The closed formulae by Tihaki *et al*,[30] were also utilised to understand the discriminatory power that the samples sizes above would provide with respect to biomarker sensitivity and specificity; The following assumptions were made:

- That a sensitivity of 80 % with a precision of 5% would provide a useful biomarker capable of 'ruling out' an acute class. The same target was applied to specificity.
- We assume a prevalence of acute breathlessness of 80% as the recruitment campaign uses acute breathlessness as the initial stratification tool for recruitment and 1:5 patients recruited will be non-breathless healthy controls
- We aim to balance group sizes across classes equally

For a type 1 error rate of 0.05 and a 95% confidence interval

$N_{sensitivity} = 307$

$N_{specificity} = 1{,}230$

For a type 1 error rate of 0.05 and a 90% confidence interval

$N_{sensitivity} = 218$

$N_{specificity} = 871$

For a type 1 error rate of 0.05 and an 85% confidence interval

$N_{sensitivity} = 166$

$N_{specificity} = 664$

For a type 1 error rate of 0.05 and an 80% confidence interval

$N_{sensitivity} = 131$

$N_{specificity} = 524$

For peer review only - http://bmjopen.bmj.com/site/about/guidelines.xhtml

Therefore, we are powered to identify sensitive biomarkers (≥ 80%) of acute breathlessness with a maximum marginal error in the estimate for sensitivity not exceeding 5% with 95% confidence. Similarly, we are powered to identify specific biomarkers (≥ 80%) of acute breathlessness with a maximum marginal error in the estimate for specificity not exceeding 5% with 80% confidence.

For the primary analysis the outcome will be treated as a nominal variable with levels (i) acute heart failure (ii) community acquired pneumonia (iii) adult exacerbations of asthma and COPD and (iv) acute exacerbations in school age children treated in hospital for severe asthma attacks.

The relationship between the primary outcome and the exhaled breath VOC biomarkers will be modelled using multinomial logistic regression. In addition to metabolomics markers the following independent variables will be included in the model: clinical uncertainty score on a 100 mm VAS scale, age, and a validated co morbidity score (the Charlson comorbidity score)[31, 32].

Receiver operator analyses will be utilised to generate ROC curves for individual and multiple panels VOC predictors in the primary analysis.

To understand the dynamic profile of breath biomarkers during (i) the acute state and (ii) in the chronic state up to 6 months post exacerbation, a repeated measures model with a random intercept and random effect for time will be fitted, the random effects will be fitted for each patient. For the repeated measures mixed model an unstructured covariance will be assumed. To evaluate the relationship between breath biomarkers and hospital readmission at 30 and 60 days Cox proportional hazards and frailty models will be utilised [33]. Analysis of Multivariate Survival Data, [CITE] competing risk models and joint models will be fitted [34]. Relationship between death and breath biomarkers will be evaluated using a logistic regression model. Changes in outcome measures will be measured appropriately for each variable (e.g. paired t-test, Mann-Whitney, repeated measures analysis).Tables of descriptive statistics will be compiled for all key variables

All analysis will be performed using R 3.5.0 https://www.r-project.org/.

For peer review only - http://bmjopen.bmj.com/site/about/guidelines.xhtml

2.4. Discovery and Replication studies

Specific indicator conditions have been selected for targeted recruitment according to their high prevalence and unmet need, their high morbidity and mortality and the need to develop better diagnostic and prognostic algorithms in acute care pathways.

The indicator diagnoses of interest are (i) exacerbations of adult asthma and COPD, (ii) community acquired pneumonia (iii) acute heart Failure and (iv) exacerbation in school age children treated in hospital for severe asthma attacks.

Patient level clinico-pathological and outcome data (spanning the entire acute pathway) will be collected in parallel to breath sampling. In addition, breath samples will be acquired in the stable state post exacerbation (**Figure 3**).

Age matched healthy volunteers will be recruited where possible at separate visits. For acute admission the study team will approach the spouse, parent or sibling of the index case and seek informed consent for study assessments. All healthy subjects will undergo two assessments separated by a duration of 8-16 weeks to match the acute and recovery time points elapsed in their index case/partner/spouse/sibling/child. Additional healthy volunteers will be identified from local recruitment databases and via advertising

2.4.1. Discovery Phase (Project months 1-24):

The aim of the discovery phase is to identify putative discriminatory breath VOC, using both offline and online technologies.

Pre-planned recruitment of acutely breathless patients will be enriched into the following disease strata following senior clinical decision maker assessment and within 24 hours of acute admission. Acute adult heart failure (n=55), adult community acquired pneumonia (n=55), adult exacerbations of asthma (n=55) and COPD (n=55), acute severe asthma attacks in school age children (n=55). Additional age matched healthy volunteers (n=55 adults and 50 children) will be identified as a non-disease reference group.

For peer review only

For peer review only - http://bmjopen.bmj.com/site/about/guidelines.xhtml

2.4.2. Replication Phase (years 3-4)

The aim of the replication phase is to replicate putative discriminatory breath VOC /VOC signatures identified in the discovery phase.

The aim of the discovery phase is to identify putative discriminatory breath VOC, using both offline and online technologies.

Pre-planned recruitment of acutely breathless patients will be enriched into the following disease strata following senior clinical decision maker assessment and within 24 hours of acute admission. Acute adult heart failure (n=55), adult community acquired pneumonia (n=55), adult exacerbations of asthma (n=55) and COPD (n=55), acute severe asthma attacks in school age children (n=55). Additional age matched healthy volunteers (n=55 adults and 50 children) will be identified as a non-disease reference group.

Total combined sample size of the discovery and replication phases = 650 participants

2.5. Schedule of assessments

A schedule of acute assessments is outlined below and aligns to the movement of acute patients through the clinical care pathway and the overall aim of developing a complete phenotypic picture of acutely breathless patients.

2.5.1. Defining acute breathlessness

At presentation (within 24 hours of admission) to one of three acute admissions units potentially eligible patients will be identified following confirmation of acute breathlessness, identified as (i) patient defined acute breathlessness and/or (ii) 1 unit increase above patient reported baseline in the extended medical research council (eMRC) dyspnoea score [35, 36] and at least one of the indicator diagnoses identified as the primary clinical diagnosis by a senior clinical decision maker.

For peer review only - http://bmjopen.bmj.com/site/about/guidelines.xhtml

2.5.2 Informed consent

Patients meeting the pre-specified definition of acute breathlessness will be approached for informed consent in to the breath VOC biomarker study. Only patients that are eligible to give full written informed consent will be recruited.

2.5.3 Collection of blood based pathology markers

Collection of the blood biomarkers CRP, BNP, Troponin-I and blood eosinophil count will be performed both acutely and following recovery, when not taken as part of clinical care pathway. These are currently used in profiling acutely breathless patients in clinical practice (**Table1**).

Test	ANALYSER/METHOD	LOWER LIMIT OF DETECTION	UPPER LIMIT OF DETECTION
C-Reactive protein (CRP)	Siemens Advia Chemistry XPT, PEG enhanced immunoturbidimetric. Siemens Advia 1800, PEG enhanced immunoturbidimetric	5 mg/L	Diluted to result
B-type natriuretic peptide (BNP)	Siemens Advia Centaur XPT, two-site sandwich immunoassay using direct chemiluminescent technology	2.0 pg/mL	1445 pg/mL
Troponin-I	Abbott Architect i2000SR, three-site sandwich immunoassay using direct chemiluminescent technology (CMIA).	5.0 ng/L	50,000 ng/L

Table (1): Type of analyser and methodology used for blood biomarker calculation. The table outlines analyser make, methodology, upper and lower limits of detection as per the University Hospitals of Leicester NHS Foundation trust laboratory guidelines.

2.5.4 Breath VOC sampling

Offline breath sampling using GC-MS coupled with a standardised and CE marked breath sampler-ReCIVA®[37] and comprehensive two-dimensional gas chromatography-mass spectrometry, coupled with a standardised and CE marked breath sampler (ReCIVA® GCxGC-MS) will be performed . Additionally the following online technologies, proton transfer mass spectroscopy (PTR-MS), gas chromatography - ion mobility spectroscopy (GC-IMS) and atmospheric pressure chemical ionisation-

For peer review only - http://bmjopen.bmj.com/site/about/guidelines.xhtml

mass spectrometry (APCI-MS) will be evaluated according to the sampling strategy outlined in

	COPD		Asthma		Pneumonia		Heart Failure		Healthy		Paediatrics	
Time point	1	2	1	2	1	2	1	2	1	2	1	2
Written informed consent	x		x		x		x		x		x	
**Volatile organic compound (VOC) sampling**												
ReCIVA- gas chromatography and mass spectrometry (GC-MS)	x	x	x	x	x	x	x	x	x	x	x	x
ReCIVA- comprehensive two-dimensional gas chromatography (GCxGC-MS)	x	x	x	x	x	x	x	x	x	x	x	x
Atmospheric pressure chemical ionisation- mass spectrometry (APCI-MS)	x	x	x	x	x	x	x	x	x	x	x	x
Proton transfer reaction mass spectrometry (PTRMS)		x		x		x		x	x	x	x	x
Gas chromatography - ion mobility spectrometry (GC-IMS)	x		x		x		x				x	x
**Pathology blood tests**												
Full blood count (including differential cell count)	x	x	x	x	x	x	x	x	x	x	x	x
Brain natriuretic peptide (BNP) [pg/mL]	x	x	x		x		x	x	x			
Troponin-I]ng/L]	x		x		x		x		x			
C-Reactive protein (CRP) [mg/L]	x	x	x	x	x	x	x	x	x	x	x	x
**Lung function tests**											x	x
Hand held forced oscillation technique (FOT)	x	x	x	x	x	x	x	x	x	x		
Fractional exhaled nitric oxide (FeNO) - Flow rate 50 [ml/s]			x	x							x	x
Spontaneous sputum sample	x	x	x	x	x	x	x	x	x	x		
Bio-banking (urine, serum, plasma. sputum supernatants & plugs)	x	x	x	x	x	x	x	x	x	x	x	x
Transthoracic echocardiography	x						x					

section 3, **Figure 3 and Table 2**.

Table (2): Summary of baseline and follow up assessments. The table summarises key assessments carried out at different time points during the study. The participants may undertake any combination of the investigations listed at any of these time points.

For peer review only - http://bmjopen.bmj.com/site/about/guidelines.xhtml

2.5.5. Collection of additional samples for future biomarker campaigns

Collection of additional biomarkers for future biomarker discovery campaigns including (i) a urine sample, (ii) blood samples, up to 85mls for DNA, RNA, plasma and serum and peripheral blood cell flow cytometry in selected subjects and (iii) spontaneous or induced sputum samples (plugs and supernatants) will be carried out (**Table 2**).

All samples will be collected at time point 1 and at 2 (**Figure 3**). The additional samples will be used for future omics analyses, these may include detailed analysis of the metagenome in sputum and proteomics applied to urine and serum samples.

2.5.6. Physiological characterisation

Physiological measures of lung function will be performed in acutely ill participants and at recovery including, (i) Hand held forced oscillation technique (FOT): an easily accessible measure of lung function. Patients favour this to spirometry as it is effort independent, unlike spirometry and requires less than a minute of quiet tidal breathing to obtain triplicate high quality measurements [38], (ii) Fractional exhaled nitric oxide (FeNO): A measure of airway inflammation in asthmatic patients[39, 40] (iii) Echocardiography: Two dimensional transthoracic echocardiography was performed in heart failure and COPD patients using an iE 33 system with S5-1 transducer (frequency transmitted 1.7 MHz, received 3.4 MHz; Philips Medical Systems, Best, The Netherlands). Standard techniques as per American Society of Echocardiography guidelines (ASE)[41] were used to acquire 2D, colour and Doppler images in conventional parasternal long-axis, short-axis and apical 4-, 2- and 3-

For peer review only - http://bmjopen.bmj.com/site/about/guidelines.xhtml

chamber views. Left ventricular ejection fraction (LVEF) was calculated using the biplane method of discs formula (Simpson's rule) to derive left ventricular volume indices.

2.5.7 Recovery follow up

The recovery from an acute exacerbation will be confirmed and identified as patient defined recovery, at the recovery study visit (time point 2) up to six months post-acute event. The schedule of assessments at the recovery visit is outlined (**Table 2).**

2.6. Clinical Adjudication:

In an effort to reduce data variability and minimise bias, an independent panel consisting of two senior acute clinicians (SS & NG) will review all pertinent clinical and diagnostic source documentation, whilst blinded to admission blood biomarkers and clinical diagnosis.

All acutely breathless adult patients' notes will be sequentially divided for adjudication. The panel will independently determine the primary diagnosis of highest probability from a list of the four potential acute indicator diagnoses in section 2.4 and mark their level of clinical certainty on a 100 mm visual analogue scale (VAS scale). The panel members will be able to review imaging, electrocardiograms (ECGs), and other relevant information but not admission blood based pathology tests.

In a subset of patients adjudication will be validated by separate panel member to ensure between observer agreement using Bland-Altman analysis and inter-rater agreement of the primary diagnosis using Kohen's kappa via repeated evaluation of a subset of cases (see statistical methods section 2.3).

2.7. Clinical Informatics:

Clinical data collection will be undertaken using a securely-hosted bespoke database system (ADD) developed within the NIHR Leicester Biomedical Research Centre – Respiratory (BRC). The system links acute admission episodes to hospital pathology records; historical respiratory physiology tests; and demographic information. The system provides functionality to validate data entry; manually verify records and highlight incomplete records. A custom VOC 'module' has been be created to

For peer review only - http://bmjopen.bmj.com/site/about/guidelines.xhtml

support data collection within the study visits (1 and 2), and standardise diagnoses and medications through the use of clinical ontologies as well as linking hospital records/tests to patient visits.

Non-ADD based clinical data (e.g. hospital admissions, re-admissions and mortality) will be extracted from the hospital data warehouse using identifiable patient identifiers, and subsequently pseudonymised prior to integration.

An informatics pipeline will be created to facilitate the transfer of chemo-metric data from remote computers to the data repository. This will include tools to (i) enforce the correct labelling of data sets (e.g. study number, visit, type/source of sample) prior to automated validated transfer to the repository; (ii) record information about the sample process; (iii) search and extract data sets from the repository for subsequent analysis. Prior to analysis, clinical and chemo-metric data will be integrated using the study number and any potentially identifiable information will be removed.

3. Breath profiling

The technologies utilised in the VOC study during discovery and replication phases are:

Offline technologies

- ReCIVA+ GC-MS
- ReCIVA + GC x GC-MS
-

Online technologies

- GC-IMS
- PTR-MS
- APCI-MS

Offline technologies will underpin the discovery analyses owing to their ability to identify chemical identity and their recognition as the analytical gold standard in exhaled breath VOC analysis [42].

For peer review only - http://bmjopen.bmj.com/site/about/guidelines.xhtml

In contrast online technologies will be utilised for VOC biomarker replication and at the recovery visits owing to their portability and potential for future point of care testing. **(Figure 4)**. A brief description of the core VOC platforms is provided below

A CE marked breath sampling device (ReCIVA®) developed by Owlstone Medical, will be used to sample breath onto adsorbent Tenax tubes. This effectively allows de-coupling of the breath sampling from the breath sensor and analysis platforms in selected patients that are not able to mobilise to a real time breath sampling device. The Owlstone ReCIVA sampler will be utilised in breath collection for offline technologies namely GC-MS and GCxGC-MS. The ReCIVA sampler is capable of entraining oxygen and is therefore suitable for patients with mild respiratory failure requiring low flow rates of oxygen to maintain target oxygen saturations [37].

3.1. Gas chromatography and mass spectroscopy (GC-MS): is a commonly applied methodology used to accurately measure trace gases in complex mixtures such as exhaled air [42]. Pre-concentrating breath volatiles by various means and subsequent analysis constitute a reliable and sensitive method for VOC analysis [43]. Despite its high sensitivity, it is however, a time consuming technique and carries a risk of contamination at the pre-concentration step. It is also not suitable for online and multiple measurements limiting its use as a point-of-care testing technology for VOC [44].

3.2. Comprehensive two-dimensional gas chromatography-mass spectrometry (GCxGC-MS): an advanced analytical technique for the analysis of complex organic matrices; its main advantage is the unparalleled separation power it affords over conventional one-dimensional chromatographic techniques [45]. Previous research, albeit sparse, has demonstrated the potential of GCxGC-MS for breath analysis with the number of VOC detected exceeding those detected by conventional GC-MS [46, 47]. GCxGC-MS of breath metabolites has been used for the identification of biomarkers related to glucose metabolism [48, 49], tuberculosis [50] and radiation response [51]. This has generated interest within the breath research community, however, such studies were conducted on a small scale (<50 patients) and involved the use of expensive detectors and modulators. Method development and analysis of the data-rich GCxGC chromatograms, however, can be time-consuming and require specialist knowledge.

For peer review only

For peer review only - http://bmjopen.bmj.com/site/about/guidelines.xhtml

3.3. Proton-transfer-reaction mass spectrometry (PTRMS): a real time technique, capable of simultaneously measuring the evolution of multiple gas metabolites from a single breath. It has been used for the identification of potential useful VOC biomarkers for diagnosis of a variety of diseases: including various cancers[52-54], liver disease[55, 56] and respiratory disease[57]. It has several advantages in clinical settings, such as the speed of sampling, the instant result achieved and the lack of need for sample storage or shipping. However, owing to the lack of pre-concentration or chromatographic separation, sensitivity and definitive compound identification can be somewhat limited when compared to GC-MS.

3.4. Gas chromatography- ion mobility spectrometry (GC-IMS): allows the detection of volatile organic compounds down to ultra-trace level (ng/L - to pg/L - range). For years, IMS has been used to discover potential discriminatory breath VOC in lung cancer [58, 59], COPD[60, 61] and asthma[61]. The technology's multiple advantages of ultra-sensitivity, portability, online sampling and short analysis time (typical analysis time of 10 minutes) with real time detection, brings a promise to provide immediate and potentially reliable results for point of care breath diagnostics. Another concept with IMS devices is that once the required breath signatures have been discovered using GC-MS, IMS offers the potential to be 'tuned' for selective detection of VOC.

3.5. Atmospheric pressure chemical ionisation- mass spectrometry (APCI-MS) semiportable compact version: is one of less sensitive but more affordable versions of mass spectrometers released to the commercial market in recent years. The device uses APCI to produce ions. Although the most common use of APCI-MS systems is the detection in liquid chromatography applications, the technique has proven to be a valuable tool for direct measurement of VOC in air[62, 63] food[64, 65] and breath[66, 67]. Recently, the technique has shown potential for online, real time profiling of pseudo-metabolites in exhaled breath [68] with sensitivity comparable with other techniques. By combining miniaturised MS technology with APCI techniques, adequate quality of on-site, real time measurements with minimal or no sample preparation requirement can be provided. This is a desirable outcome as it overcomes main limitation of using standard breath analysis method in clinical setting,

For peer review only - http://bmjopen.bmj.com/site/about/guidelines.xhtml

which is a need for breath sample collection followed by desorption and time-consuming laboratory analysis.

There remains an overall lack of standardisation and rigour across these technologies which hindered previous advancements in breath discovery; something we intend to minimize.

4. Chemometric processing and data analysis:

GC-MS breath data will be aligned, deconvoluted and the features for each participant will be extracted. The extracted features will be grouped and classified by retention index and mass spectrum. The registered and aligned data will be linked to participant meta-data to generate a breath matrix.

The breath matrix is a $n \times p$ matrix where n is the number of subjects and p is the number of VOC. The breath matrix is high dimensional with $p \gg n$ and many potentially correlated VOC. In view of this, we will employ sparse partial least squares discriminant analysis (sPLS-DA)[69] to investigate which of the VOC can identify breathlessness. We will also investigate which of the VOC can discriminate between the different disease states including acute exacerbations of asthma and COPD and Pneumonia. In addition to the supervised methods, unsupervised methods will be explored, specifically sparse principle component analysis (sPCA)[70].

Extracted VOC will also be investigated. Relationships between VOC and patient reported acute breathlessness will be analysed using logistic regression model. VOC associated with patient associated acute breathlessness will be incorporated into multinomial logistic regression models in conjunction with CRP, BNP, blood eosinophils and Troponin-I, pathology biomarkers currently in use for diagnosing undifferentiated breathlessness. In addition to the conventional binary and multinomial logistic regression models, regression models [71]..

5. Ethics and dissemination:

The study has obtained full ethical approval from the London South East Research ethics Committee, REC reference 16/LO/1747. IRAS project ID 198921. Publications will be prepared according to the MRC-EMBER consortium agreement and the University of Leicester publications policy. All

For peer review only

intended publications will be submitted to the EMBER executive board for review and comments within 60 days of journal submission. Authorship will be according to contribution and internationally recognised guidance on journal authorship.

6. Study dates: 01/2/2017 – 30/10/2020

7. Authors' contributions:

WI and SS drafted the manuscript and all co-authors critically revised and contributed to the manuscript. All co-authors contributed to the study design and development. SS is the Chief investigator for the acute VOC study.

Protocol version: Version 4, 1st April 2018

Funding statement:

This study has been funded by the Medical research Council (MRC) and Engineering and Physical Sciences Research Council (EPSRC) and is sponsored by the University of Leicester. The children's component of this study is also being supported by The Midlands Asthma and Allergy Research Association (MAARA). Grant number [MR/N005880/1]

Competing interests: SS has performed advisory services for Owlstone Medical.

For peer review only

Figure legends:

Figure (1) Relationship between lung proximity and degree of invasiveness of different lung matrices. The figure plots the level of invasiveness of various lung matrices in relation to their proximity to the lung. Given their pathological relevance, the degree of invasiveness of bronchoalveolar lavage (BAL) and lung biopsy makes them less favourable in diagnosing respiratory diseases.

Figure (2) Multi-instrument use in breath sampling. Figure illustrates the various combinations of offline and online devices used in breath sampling and the relevant pros and cons. Offline and online technologies are used for the discovery and validation phases of the study respectively.

Figure (3) Study flow chart. Figure outlines the patient journey from admission through to discharge and follow up. Participants with self-reported acute breathlessness presenting to University Hospitals of Leicester are recruited within 24 hours following a senior decision maker review. Breath sampling is carried out on the first visit, at recruitment, and the second visit, up to 6 months after discharge. Patients are admitted through the standard operational emergency medical streaming and care pathways at the University Hospitals of Leicester NHS Trust. Early outcomes (hospital readmission) are measured at 30 and 60 days and late outcomes (mortality) including respiratory and all-cause mortality are measured at 2 years. Assessments carried out at each time point are summarised in **Table 1**.

Figure (4) Multi-instrument use in breath sampling. Operational space of the analytical technologies used in EMBER for the analysis of volatile organic compounds (VOC) in exhaled breath, including proton transfer reaction-mass spectrometry (PTR-MS), atmospheric pressure chemical ionisation-mass spectrometry (CMS), gas chromatography-ion mobility spectrometry (GC-IMS), gas

For peer review only - http://bmjopen.bmj.com/site/about/guidelines.xhtml

chromatography-mass spectrometry (GC-MS) and two-dimensional gas chromatography-mass spectrometry (GCxGC). Comparing the typical molar mass range detectable; selectivity in detection owing to the type of ionisation involved and the proton affinity of analytes; and the inclusion of a chromatographic separation affecting total time of analysis. The online technologies involving chemical ionisation (PTR-MS, CMS and GC-IMS) can be used in-clinic owing to short analysis times, but only detect lower molar mass molecules with a proton affinity higher than 697 KJ mol-1. Offline chromatographic techniques (GC-MS and GCxGC) detect a wider range of compounds independent of proton affinity; however, the techniques have longer analysis times and involve sample transportation and storage.

For peer review only

For peer review only - http://bmjopen.bmj.com/site/about/guidelines.xhtml

8. References

1. Stephenson, J.J., et al., *Clinical and economic burden of dyspnea and other COPD symptoms in a managed care setting.* Int J Chron Obstruct Pulmon Dis, 2017. **12**: p. 1947-1959.
2. David Ponka, M.K., *Top differential diagnoses in family medicine Dyspnoea Can Fam Physician.* Canadian Family Physician, 2008.
3. Figarska, S.M., H.M. Boezen, and J.M. Vonk, *Dyspnea severity, changes in dyspnea status and mortality in the general population: the Vlagtwedde/Vlaardingen study.* European Journal of Epidemiology, 2012. **27**(11): p. 867-876.
4. Parshall, M.B., et al., *An Official American Thoracic Society Statement: Update on the Mechanisms, Assessment, and Management of Dyspnea.* American Journal of Respiratory and Critical Care Medicine, 2012. **185**(4): p. 435-452.
5. Lim, W.S., et al., *BTS guidelines for the management of community acquired pneumonia in adults: update 2009.* Thorax, 2009. **64 Suppl 3**: p. iii1-55.
6. Leuzzi, G., et al., *C-reactive protein level predicts mortality in COPD: a systematic review and meta-analysis.* Eur Respir Rev, 2017. **26**(143).
7. Moe, G.W., *B-type natriuretic peptide in heart failure.* Curr Opin Cardiol, 2006. **21**(3): p. 208-14.
8. Ponikowski, P., et al., *2016 ESC Guidelines for the diagnosis and treatment of acute and chronic heart failure: The Task Force for the diagnosis and treatment of acute and chronic heart failure of the European Society of Cardiology (ESC)Developed with the special contribution of the Heart Failure Association (HFA) of the ESC.* Eur Heart J, 2016. **37**(27): p. 2129-2200.
9. Alex Sakula, *Charcot leyden crystals and crushmann spirals.* Thorax, 1986.
10. Calhoun, W.J., J. Sedgwick, and W.W. Busse, *The role of eosinophils in the pathophysiology of asthma.* Ann N Y Acad Sci, 1991. **629**: p. 62-72.
11. Pavord, I.D., *Eosinophilic phenotypes of airway disease.* Ann Am Thorac Soc, 2013. **10 Suppl**: p. S143-9.
12. Tashkin, D.P. and M.E. Wechsler, *Role of eosinophils in airway inflammation of chronic obstructive pulmonary disease.* International Journal of Chronic Obstructive Pulmonary Disease, 2018. **13**: p. 335-349.
13. Bafadhel, M., et al., *Blood Eosinophils to Direct Corticosteroid Treatment of Exacerbations of Chronic Obstructive Pulmonary Disease: A Randomized Placebo-Controlled Trial.* American Journal of Respiratory and Critical Care Medicine, 2012. **186**(1): p. 48-55.
14. Bedossa, P. and K. Patel, *Biopsy and Noninvasive Methods to Assess Progression of Nonalcoholic Fatty Liver Disease.* Gastroenterology, 2016. **150**(8): p. 1811-1822 e4.
15. Fens, N., et al., *Exhaled breath analysis by electronic nose in airways disease. Established issues and key questions.* Clin Exp Allergy, 2013. **43**(7): p. 705-15.
16. Probert, C.S., et al., *Volatile organic compounds as diagnostic biomarkers in gastrointestinal and liver diseases.* J Gastrointestin Liver Dis, 2009. **18**(3): p. 337-43.
17. Schubert, J.K., et al., *Breath analysis in critically ill patients: potential and limitations.* Expert Rev Mol Diagn, 2004. **4**(5): p. 619-29.

For peer review only - http://bmjopen.bmj.com/site/about/guidelines.xhtml

18. Fowler, S.J., et al., *Surveillance for lower airway pathogens in mechanically ventilated patients by metabolomic analysis of exhaled breath: a case-control study.* Thorax, 2015. **70**(4): p. 320-5.
19. Florian Gahleitner, C.G.-H., Caroline S Beardsmore, Hitesh C Pandya, CL Paul Thomas, *Metabolomics pilot study to identify VOC of childhood asthma in exhaled breath.* Bioanalysis, 2013.
20. Christiansen, A., et al., *A systematic review of breath analysis and detection of volatile organic compounds in COPD.* J Breath Res, 2016. **10**(3): p. 034002.
21. de Vries, R., et al., *Integration of electronic nose technology with spirometry: validation of a new approach for exhaled breath analysis.* J Breath Res, 2015. **9**(4): p. 046001.
22. Shafiek, H., et al., *Using the Electronic Nose to Identify Airway Infection during COPD Exacerbations.* PLoS One, 2015. **10**(9): p. e0135199.
23. Finamore, P., et al., *Analysis of volatile organic compounds: an innovative approach to heart failure characterization in older patients.* J Breath Res, 2018. **12**(2): p. 026007.
24. *MRC Molecular Pathology, Review 20 Aug.*
25. Medical Research Council, *Molecular Pathology Nodes*. 2014: MRC website.
26. Corradi, M., et al., *Aldehydes in exhaled breath condensate of patients with chronic obstructive pulmonary disease.* Am J Respir Crit Care Med, 2003. **167**(10): p. 1380-6.
27. Ibrahim, B., et al., *Non-invasive phenotyping using exhaled volatile organic compounds in asthma.* Thorax, 2011. **66**(9): p. 804-9.
28. Larstad, M., et al., *Determination of malondialdehyde in breath condensate by high-performance liquid chromatography with fluorescence detection.* J Chromatogr B Analyt Technol Biomed Life Sci, 2002. **766**(1): p. 107-14.
29. F. Y. Hsieh, D.A.B., Michael D. Larsen,, *A simple method of sample size calculation for linear and logistic regression* Statis Med, 1998.
30. Hajian-Tilaki, K., *Sample size estimation in diagnostic test studies of biomedical informatics.* J Biomed Inform, 2014. **48**: p. 193-204.
31. Quan, H., et al., *Updating and validating the Charlson comorbidity index and score for risk adjustment in hospital discharge abstracts using data from 6 countries.* Am J Epidemiol, 2011. **173**(6): p. 676-82.
32. Roffman, C.E., J. Buchanan, and G.T. Allison, *Charlson Comorbidities Index.* J Physiother, 2016. **62**(3): p. 171.
33. Bjarnason, H. and P. Hougaard, *Fisher information for two gamma frailty bivariate Weibull models.* Lifetime Data Anal, 2000. **6**(1): p. 59-71.
34. Lawrence Gould, A., et al., *Joint modeling of survival and longitudinal non-survival data: current methods and issues. Report of the DIA Bayesian joint modeling working group.* Stat Med, 2015. **34**(14): p. 2181-95.
35. Steer, J., et al., *Dyspnoea severity and pneumonia as predictors of in-hospital mortality and early readmission in acute exacerbations of COPD.* Thorax, 2012. **67**(2): p. 117-21.
36. Tsimogianni, A.M., et al., *Predictors of Outcome After Exacerbation of Chronic Obstructive Pulmonary Disease.* Journal of General Internal Medicine, 2009. **24**(9): p. 1043-1048.
37. Kitchen, S., et al., *LATE-BREAKING ABSTRACT: Breathe free: Open source development of a breath sampler by a consortium of breath researchers.* European Respiratory Journal, 2015. **46**(suppl 59).
38. Oostveen, E., et al., *The forced oscillation technique in clinical practice: methodology, recommendations and future developments.* Eur Respir J, 2003. **22**(6): p. 1026-41.
39. Pendharkar, S. and S. Mehta, *The clinical significance of exhaled nitric oxide in asthma.* Canadian Respiratory Journal : Journal of the Canadian Thoracic Society, 2008. **15**(2): p. 99-106.
40. Yates, D.H., *Role of exhaled nitric oxide in asthma.* Immunol Cell Biol, 2001. **79**(2): p. 178-90.

For peer review only

For peer review only - http://bmjopen.bmj.com/site/about/guidelines.xhtml

41. Lang, R.M., et al., *Recommendations for chamber quantification: a report from the American Society of Echocardiography's Guidelines and Standards Committee and the Chamber Quantification Writing Group, developed in conjunction with the European Association of Echocardiography, a branch of the European Society of Cardiology.* J Am Soc Echocardiogr, 2005. **18**(12): p. 1440-63.
42. J. Throck Watson, O.D.S., *Introduction to Mass Spectrometry: Instrumentation, Applications, and Strategies for Data Interpretation, 4th Edition*. 2007: Wiley. 862.
43. Buszewski, B., et al., *Human exhaled air analytics: biomarkers of diseases.* Biomed Chromatogr, 2007. **21**(6): p. 553-66.
44. Amann, A., et al., *Applications of breath gas analysis in medicine.* International Journal of Mass Spectrometry, 2004. **239**(2-3): p. 227-233.
45. Almstetter, M.F., P.J. Oefner, and K. Dettmer, *Comprehensive two-dimensional gas chromatography in metabolomics.* Anal Bioanal Chem, 2012. **402**(6): p. 1993-2013.
46. Das, M.K., et al., *Investigation of Gender-Specific Exhaled Breath Volatome in Humans by GCxGC-TOF-MS.* Analytical Chemistry, 2014. **86**(2): p. 1229-1237.
47. Phillips, M., et al., *Detection of an extended human volatome with comprehensive two-dimensional gas chromatography time-of-flight mass spectrometry.* PLoS One, 2013. **8**(9): p. e75274.
48. Gruber, B., et al., *Vacuum ultraviolet absorption spectroscopy in combination with comprehensive two-dimensional gas chromatography for the monitoring of volatile organic compounds in breath gas: A feasibility study.* J Chromatogr A, 2016. **1464**: p. 141-6.
49. Gruber, B., et al., *Breath gas monitoring during a glucose challenge by a combined PTR-QMS/GCxGC-TOFMS approach for the verification of potential volatile biomarkers.* J Breath Res, 2016. **10**(3): p. 036003.
50. Mellors, T.R., et al., *A new method to evaluate macaque health using exhaled breath: A case study of M. tuberculosis in a BSL-3 setting.* J Appl Physiol (1985), 2017. **122**(3): p. 695-701.
51. Phillips, M., et al., *Breath biomarkers of whole-body gamma irradiation in the Gottingen minipig.* Health Phys, 2015. **108**(5): p. 538-46.
52. Bajtarevic, A., et al., *Noninvasive detection of lung cancer by analysis of exhaled breath.* BMC Cancer, 2009. **9**: p. 348.
53. Lavra, L., et al., *Investigation of VOCs associated with different characteristics of breast cancer cells.* Sci Rep, 2015. **5**: p. 13246.
54. Zhou, W., et al., *Exhaled breath online measurement for cervical cancer patients and healthy subjects by proton transfer reaction mass spectrometry.* Anal Bioanal Chem, 2017. **409**(23): p. 5603-5612.
55. Fernández del Río, R., et al., *Volatile Biomarkers in Breath Associated With Liver Cirrhosis — Comparisons of Pre- and Post-liver Transplant Breath Samples.* EBioMedicine, 2015. **2**(9): p. 1243-1250.
56. O'Hara, M.E., et al., *Limonene in exhaled breath is elevated in hepatic encephalopathy.* J Breath Res, 2016. **10**(4): p. 046010.
57. Tomasz Ligor, C.A., Anton Amann, Konrad Schwarz, Bogusaw Buszewski, Wojciech ebrowski,, *<Comparison of Proton Transfer Reaction-Mass Spectrometry and Gas Chromatography-Mass Spectrometry in Analysis of Breath Samples.pdf>*. Chem. Anal., 2009.
58. Darwiche, K., et al., *Bronchoscopically obtained volatile biomarkers in lung cancer.* Lung, 2011. **189**(6): p. 445-52.
59. Handa, H., et al., *Exhaled Breath Analysis for Lung Cancer Detection Using Ion Mobility Spectrometry.* PLoS ONE, 2014. **9**(12): p. e114555.
60. Bessa, V., et al., *Detection of volatile organic compounds (VOCs) in exhaled breath of patients with chronic obstructive pulmonary disease (COPD) by ion mobility spectrometry.* International Journal for Ion Mobility Spectrometry, 2011. **14**(1): p. 7-13.

For peer review only

For peer review only - http://bmjopen.bmj.com/site/about/guidelines.xhtml

61. Schivo, M., et al., *Mobile instrumentation platform to distinguish airway disorders.* Journal of breath research, 2013. **7**(1): p. 017113-017113.
62. Badjagbo, K., et al., *Direct atmospheric pressure chemical ionization-tandem mass spectrometry for the continuous real-time trace analysis of benzene, toluene, ethylbenzene, and xylenes in ambient air.* J Am Soc Mass Spectrom, 2009. **20**(5): p. 829-36.
63. Healy, R.M., et al., *A multi-year study of VOC emissions at a chemical waste disposal facility using mobile APCI-MS and LPCI-MS instruments.* Environ Pollut, 2018. **232**: p. 220-228.
64. Linforth, R. and A.J. Taylor, *Persistence of Volatile Compounds in the Breath after Their Consumption in Aqueous Solutions.* Journal of Agricultural and Food Chemistry, 2000. **48**(11): p. 5419-5423.
65. Perez-Hurtado, P., et al., *Direct analysis of volatile organic compounds in foods by headspace extraction atmospheric pressure chemical ionisation mass spectrometry.* Rapid Commun Mass Spectrom, 2017. **31**(22): p. 1947-1956.
66. Berchtold, C., L. Meier, and R. Zenobi, *Evaluation of extractive electrospray ionization and atmospheric pressure chemical ionization for the detection of narcotics in breath.* International Journal of Mass Spectrometry, 2011. **299**(2-3): p. 145-150.
67. Kapishon, V., et al., *Atmospheric pressure chemical ionization mass spectrometry of pyridine and isoprene: potential breath exposure and disease biomarkers.* J Breath Res, 2013. **7**(2): p. 026005.
68. Heaney, L.M., et al., *Real-time monitoring of exhaled volatiles using atmospheric pressure chemical ionization on a compact mass spectrometer.* Bioanalysis, 2016. **8**(13): p. 1325-36.
69. Lê Cao, K.-A., S. Boitard, and P. Besse, *Sparse PLS discriminant analysis: biologically relevant feature selection and graphical displays for multiclass problems.* BMC Bioinformatics, 2011. **12**: p. 253-253.
70. Zou, H., T. Hastie, and R. Tibshirani, *Sparse Principal Component Analysis.* Journal of Computational and Graphical Statistics, 2006. **15**(2): p. 265-286.
71. Zou H, H.T., *Regularization and Variable Selection via the Elastic Net.* Journal of the Royal Statistical Society, 2005.

For peer review only - http://bmjopen.bmj.com/site/about/guidelines.xhtml

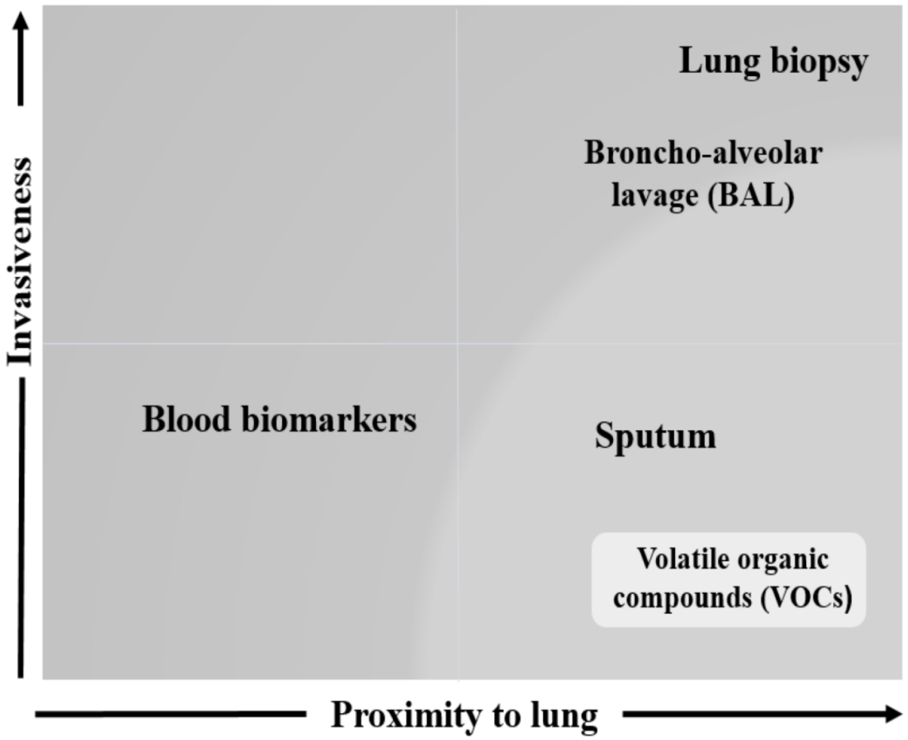

Figure (1): Relationship between lung proximity and degree of invasiveness of different lung matrices. The figure plots the level of invasiveness of various lung matrices in relation to their proximity to the lung. Given their pathological relevance, the degree of invasiveness of bronchoalveolar lavage (BAL) and lung biopsy makes them less favourable in diagnosing respiratory diseases.

144x115mm (300 x 300 DPI)

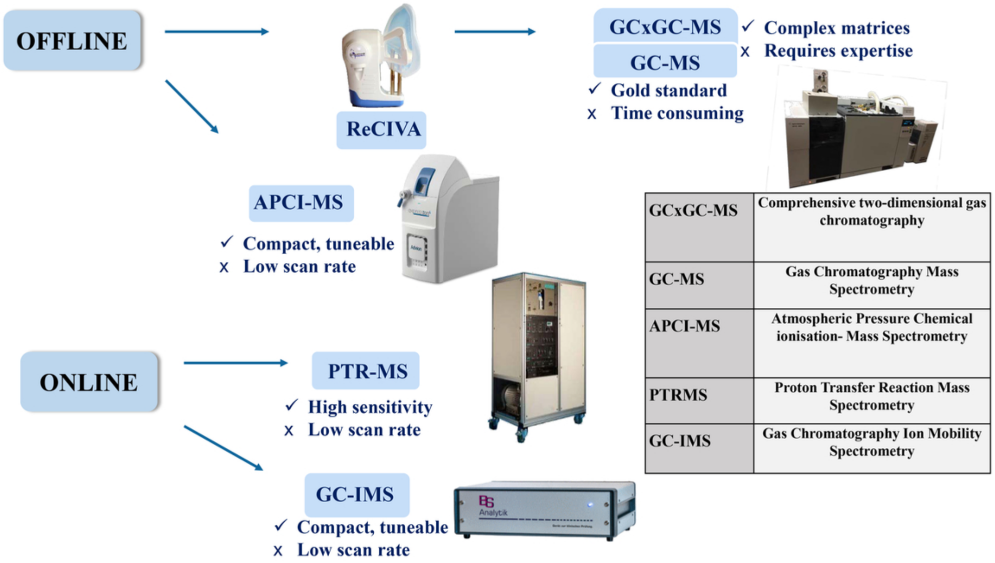

Figure (2): Multi-instrument use in breath sampling. Figure illustrates the various combinations of offline and online devices used in breath sampling and the relevant pros and cons. Offline and online technologies are used for the discovery and validation phases of the study respectively.

97x55mm (300 x 300 DPI)

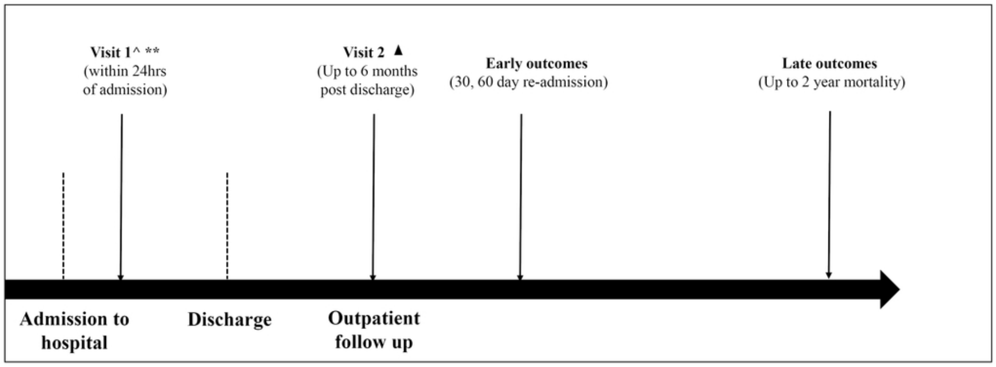

^ Following senior decision maker review
** Breath sampling (ReCIVA-GC-MS, ReCIVA- GCxGC-MS, GC-IMS, APCI-MS)
▲ Breath sampling (ReCIVA-GC-MS, ReCIVA- GCxGC-MS, PTR-MS, APCI-MS)
---- Following University Hospitals of Leicester streaming and clinical care pathways

Figure (3): Study flow chart. Figure outlines the patient journey from admission through to discharge and follow up. Participants with self-reported acute breathlessness presenting to University Hospitals of Leicester are recruited within 24 hours following a senior decision maker review. Breath sampling is carried out on the first visit, at recruitment, and the second visit, up to 6 months after discharge. Patients are admitted through the standard operational emergency medical streaming and care pathways at the University Hospitals of Leicester NHS Trust. Early outcomes (hospital readmission) are measured at 30 and 60 days and late outcomes (mortality) including respiratory and all-cause mortality are measured at 2 years. Assessments carried out at each time point are summarised in Table 1.

86x43mm (300 x 300 DPI)

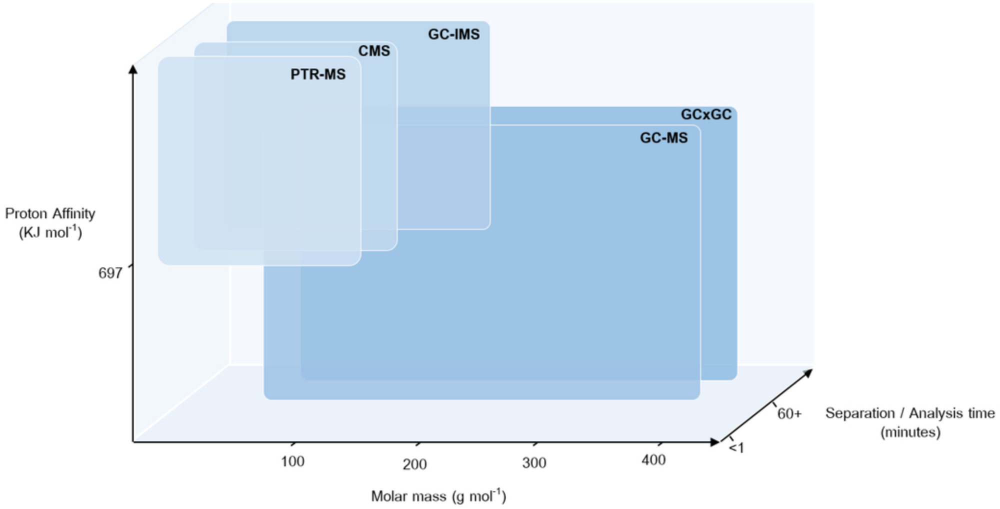

Figure (4): Multi-instrument use in breath sampling. Operational space of the analytical technologies used in EMBER for the analysis of volatile organic compounds (VOC) in exhaled breath, including proton transfer reaction-mass spectrometry (PTR-MS), atmospheric pressure chemical ionisation-mass spectrometry (CMS), gas chromatography-ion mobility spectrometry (GC-IMS), gas chromatography-mass spectrometry (GC-MS) and two-dimensional gas chromatography-mass spectrometry (GCxGC). Comparing the typical molar mass range detectable; selectivity in detection owing to the type of ionisation involved and the proton affinity of analytes; and the inclusion of a chromatographic separation affecting total time of analysis. The online technologies involving chemical ionisation (PTR-MS, CMS and GC-IMS) can be used in-clinic owing to short analysis times, but only detect lower molar mass molecules with a proton affinity higher than 697 KJ mol-1. Offline chromatographic techniques (GC-MS and GCxGC) detect a wider range of compounds independent of proton affinity; however, the techniques have longer analysis times and involve sample transportation and storage.

88x44mm (300 x 300 DPI)

BMJ Open

Assessment of Breath Volatile Organic Compounds in Acute Cardio-respiratory Breathlessness: A protocol describing a Prospective Real World Observational Study

Journal:	*BMJ Open*
Manuscript ID	bmjopen-2018-025486.R1
Article Type:	Protocol
Date Submitted by the Author:	11-Oct-2018
Complete List of Authors:	Ibrahim, Wadah; Leicester NIHR Biomedical Research Centre (Respiratory theme); College of Life Sciences, Department of Infection, Immunity and Inflammation, University of Leicester<br>Wilde, Michael; University of Leicester, Department of Chemistry<br>Cordell, Rebecca; University of Leicester, Department of Chemistry<br>Salman, Dahlia; Loughborough University, Department of Chemistry<br>Ruszkiewicz, Dorota; Loughborough University, Department of Chemistry<br>Bryant, Luke; University of Leicester, Department of Chemistry<br>Richardson, Matthew; Leicester NIHR Biomedical Research Centre (Respiratory theme); University of Leicester, Infection, Inflammation and Immunity<br>Free, Robert; Leicester NIHR Biomedical Research Centre (Respiratory theme); University of Leicester, Infection, Inflammation and Immunity<br>Zhao, Bo; Leicester NIHR Biomedical Research Centre (Respiratory theme); University of Leicester, Infection, Inflammation and Immunity<br>Yousuf, Ahmed; University of Leicester, Infection, Inflammation and Immunity; Leicester NIHR Biomedical Research Centre (Respiratory theme)<br>White, Christobelle; University of Leicester, Infection, Inflammation and Immunity; Leicester NIHR Biomedical Research Centre (Respiratory theme)<br>Russell, Richard; University of Leicester, Infection, Inflammation and Immunity; Leicester NIHR Biomedical Research Centre (Respiratory theme)<br>Jones, Sheila; University Hospitals of Leicester NHS Trust<br>Patel, Bharti; University Hospitals of Leicester NHS Trust<br>Awal, Asia; University Hospitals of Leicester NHS Trust<br>Phillips, Rachael; NIHR Leicester Clinical Research Facility<br>Fowkes, Graham; NIHR Leicester Clinical Research Facility<br>McNally, Teresa; Paediatric Clinical Investigation Centre<br>Foxon, Clare; Paediatric Clinical Investigation Centre<br>Bhatt, Hetan; Leicester NIHR Biomedical Research Centre (Respiratory theme)<br>Peltrini, Rosa; Leicester NIHR Biomedical Research Centre (Respiratory theme); University of Leicester, Department Infection, Inflammation and Immunity<br>Singapuri, Amisha; Leicester NIHR Biomedical Research Centre (Respiratory theme)<br>Hargadon, Beverley; Leicester NIHR Biomedical Research Centre

(Respiratory theme); University of Leicester, Infection, Inflammation and Immunity
Suzuki, T; Department of Cardiovascular Sciences, University of Leicester, , Cardiovascular Research Centre; Leicester NIHR Biomedical Research Centre (Cardiovascular theme)
Ng, Leong; Department of Cardiovascular Sciences, University of Leicester, Cardiovascular Research Centre; Leicester NIHR Biomedical Research Centre (Cardiovascular theme)
Gaillard, Erol; Leicester NIHR Biomedical Research Centre (Respiratory theme); Paediatric Clinical Investigation Centre
Beardsmore, Caroline; Leicester NIHR Biomedical Research Centre (Respiratory theme); Paediatric Clinical Investigation Centre
Ryanna, Kimuli; University Hospitals of Leicester NHS Trust
Pandya, Hitesh; Discovery Medicine, Respiratory Therapeutic Area, GlaxoSmithKline PLC
Coates, Tim; University of Leicester, Infection, Inflammation and Immunity; Department of Cardiovascular Sciences, University of Leicester
Monks, Paul; University of Leicester, Infection, Inflammation and Immunity; University of Leicester, Department of Chemistry
Greening, Neil; Leicester NIHR Biomedical Research Centre (Respiratory theme); University of Leicester, Infection, Inflammation and Immunity
Brightling, Christopher; Leicester NIHR Biomedical Research Centre (Respiratory theme); University of Leicester, Infection, Inflammation and Immunity
Thomas, Paul; Loughborough University, Department of Chemistry
Siddiqui, Salman; Leicester NIHR Biomedical Research Centre (Respiratory theme); University of Leicester, Infection, Inflammation and Immunity

**Primary Subject Heading**:	Respiratory medicine
Secondary Subject Heading:	Research methods, Respiratory medicine
Keywords:	Breathlessness, Observational study, Volatile Organic Compound, Breath analysis

SCHOLARONE™
Manuscripts

Assessment of Breath Volatile Organic Compounds in Acute Cardio-respiratory Breathlessness: A protocol describing a Prospective Real World Observational Study

Wadah Ibrahim[1,2], Michael Wilde[3], Rebecca Cordell[3], Dahlia Salman[4], Dorota Ruszkiewicz[4], Luke Bryant[3], Matthew Richardson[1,2], Robert C. Free[1,2], Bo Zhao[1,2], Ahmed Yousuf[1,2], Christobelle White[1,2], Richard J Russell[1,2], Sheila Jones[5], Bharti Patel[5], Asia Awal[5], Rachael Phillips[6], Graham Fowkes[6], Teresa McNally[7], Clare Foxon[7], Hetan Bhatt[1,2], Rosa Peltrini[1,2], Amisha Singapuri[1,2], Beverley Hargadon[1,2], Toru Suzuki[8,9], Leong L. Ng[8,9], Erol A Gaillard[2,7], Caroline Beardsmore[2,7], Kimuli Ryanna[5], Hitesh Pandya[11], Tim Coates[2,10], Paul S. Monks[2,3], Neil Greening[1,2], Chris E Brightling[1,2], Paul Thomas[4], Salman Siddiqui[1,2]

wadah.ibrahim@leicester.ac.uk; mjw77@leicester.ac.uk; rc145@leicester.ac.uk; d.salman@lboro.ac.uk; d.m.ruszkiewicz2@lboro.ac.uk; lb368@leicester.ac.uk; mr251@leicester.ac.uk; rcf8@leicester.ac.uk; bo.zhao@leicester.ac.uk; ajy7@leicester.ac.uk; cmjw3@leicester.ac.uk; rjr22@leicester.ac.uk; rjr22@leicester.ac.uk; sheila.jones@uhl-tr.nhs.uk; bharti.m.patel@uhl-tr.nhs.uk; asia.awal@uhl-tr.nhs.uk; rachael.phillips@uhl-tr.nhs.uk; graham.fowkes@uhl-tr.nhs.uk; tm23@leicester.ac.uk; clare.foxon@uhl-tr.nhs.uk; hetan.bhatt@uhl-tr.nhs.uk; rp417@leicester.ac.uk; amisha.singapuri@uhl-tr.nhs.uk; beverley.hargadon@uhl-tr.nhs.uk; ts263@le.ac.uk; lln1@le.ac.uk; eag15@leicester.ac.uk; csb@leicester.ac.uk; kimuli.ryanna@uhl-tr.nhs.uk; hitesh.x.pandya@gsk.com; tim.coates@leicester.ac.uk; paul.monks@leicester.ac.uk; neil.greening@leicester.ac.uk; ceb17@leicester.ac.uk; C.L.P.Thomas@lboro.ac.uk; ss338@leicester.ac.uk

Corresponding author: Professor Salman Siddiqui, ss338@leicester.ac.uk

NIHR Leicester Biomedical Research Centre (Respiratory Theme)

Glenfield Hospital,

Groby Road

Leicester

LE3 9QP

t: +44 116 256 3841

e: ss338@ leicester.ac.uk

Authors' affiliations:

[1]Leicester NIHR Biomedical Research Centre (Respiratory theme), Glenfield Hospital, Groby Road, Leicester LE3 9QP

For peer review only - http://bmjopen.bmj.com/site/about/guidelines.xhtml

[2] College of Life Sciences, Department of Infection, Immunity and Inflammation, University of Leicester, University Road, Leicester LE1 7RH

[3] Department of Chemistry, University of Leicester, University Road, Leicester, LE1 7RH

[4]Department of Chemistry, Loughborough University, Epinal Way, Loughborough, LE11 3TU

[5] University Hospitals of Leicester NHS Trust, Glenfield Hospital, Groby Road, Leicester LE3 9QP

[6] NIHR Leicester Clinical Research Facility, Research Space, Level 0, Balmoral Building, Leicester Royal Infirmary, Infirmary Square, Leicester, LE1 5WW

[7] Paediatric Clinical Investigation Centre, Leicester Respiratory Biomedical Research Centre, University of Leicester, Leicester Royal Infirmary, Leicester, LE2 7LX

[8] Department of Cardiovascular Sciences, University of Leicester, Cardiovascular Research Centre, Glenfield General Hospital, Leicester LE3 9QP.

[9] Leicester NIHR Biomedical Research Centre (Cardiovascular theme), Glenfield Hospital, Groby Road, Leicester LE3 9QP

[10]Department of Cardiovascular Sciences, University of Leicester, Level G, Jarvis building RMO, Infirmary Square, Leicester LE1 5WW

[11] Discovery Medicine, Respiratory Therapeutic Area, GlaxoSmithKline PLC, Gunnels Wood Road, Stevenage, Hertfordshire, SG1 2NY

For peer review only

For peer review only - http://bmjopen.bmj.com/site/about/guidelines.xhtml

Abstract

Introduction

Patients presenting with acute undifferentiated breathlessness are commonly encountered in admissions units across the United Kingdom. Existing blood biomarkers have clinical utility in distinguishing patients with single organ pathologies but have poor discriminatory power in multi factorial presentations. Evaluation of volatile organic compounds (VOC) in exhaled breath offers the potential to develop biomarkers of disease states that underpin acute cardio-respiratory breathlessness, owing to their proximity to the cardio-respiratory system. To date there has been no systematic evaluation of VOC in acute cardio-respiratory breathlessness. The proposed study will seek to use both offline and online VOC technologies to evaluate the predictive value of VOC in identifying common conditions that present with acute cardio-respiratory breathlessness.

Methods and analysis

A prospective real world observational study carried out across three acute admissions units within Leicestershire. Participants with self-reported acute breathlessness, with a confirmed primary diagnosis of either acute heart failure, community acquired pneumonia and acute exacerbation of asthma or COPD will be recruited within 24 hours of admission. Additionally, school age children admitted with severe asthma will be evaluated. All participants will undergo breath sampling on admission and upon recovery following discharge. A range of online technologies including: proton-transfer-reaction mass spectrometry (PTR-MS), gas chromatography ion mobility spectrometry (GC-IMS), atmospheric pressure chemical ionisation- mass spectrometry (APCI-MS) and offline technologies including gas

For peer review only - http://bmjopen.bmj.com/site/about/guidelines.xhtml

For peer review only

chromatography mass spectroscopy (GC-MS) and comprehensive two-dimensional gas chromatography-mass spectrometry (GCxGC-MS) will be utilised for VOC discovery and replication. For offline technologies a standardised CE marked breath sampling device (ReCIVA®) will be used. All recruited participants will be characterised using existing blood biomarkers including C - reactive protein (CRP), brain derived natriuretic peptide (BNP), Troponin-I and blood eosinophil levels and further evaluated using a range of standardised questionnaires, lung function testing, sputum cell counts and other diagnostic tests pertinent to acute disease.

Ethics and dissemination

The National Research Ethics Service Committee East Midlands has approved the study protocol (REC number: 16/LO/1747). IRAS 198921. Findings will be presented at academic conferences and published in peer-reviewed scientific journals. Dissemination will be facilitated via a partnership with the East Midlands Academic Health Sciences Network and via interaction with all UK funded MRC/EPSRC molecular pathology nodes.

Key words: Breathlessness; Breath analysis; Volatile Organic Compound, Observational study

For peer review only - http://bmjopen.bmj.com/site/about/guidelines.xhtml

Strengths and Limitations of this Study

- A pragmatic real world, prospective, observational study across three admission units that focuses on the systematic discovery and replication of VOC in acutely breathless patients using both online and offline technologies
- The proposed study is the largest of its kind in acute disease to characterise VOC with a range of additional assessments that will build a comprehensive phenotype of acute cardio-respiratory exacerbations
- The proposed study will build an infrastructure for research and subsequent evaluation of VOC in interventional trials within acute cardio-respiratory exacerbations
- Prior acute treatment exposure will need to be accounted for when evaluating potential discriminative biomarkers
- VOC technologies are not currently suited for deployment in patients that are of high clinical acuity

1. Introduction:

Breathlessness is a common symptom of cardio-respiratory illnesses that has a significant direct impact on patients' wellbeing as well as a substantial economic burden on healthcare systems [1]. Although its etiologies can be variable, exacerbations of common complex chronic cardio-respiratory conditions

For peer review only - http://bmjopen.bmj.com/site/about/guidelines.xhtml
For peer review only

account for approximately 70% of acute presentations with breathlessness, namely exacerbations of asthma and COPD, acute heart failure and community acquired pneumonia [2]. Moreover, moderate and severe breathlessness is significantly associated with all-cause, cardiovascular and COPD mortality[3]. As a consequence symptomatic breathlessness warrants rapid evaluation and targeted diagnostics at presentation.

Diagnostic evaluation of acute breathlessness is heavily reliant on blood based biomarkers e.g. CRP, BNP, Troponin and on occasions blood eosinophil levels. These biomarkers have clinical utility primarily in patients with single pathologies, but have poor discriminatory power in patients with multifactorial presentations of acute breathlessness[4]. There is therefore an unmet need for the development of sensitive and specific biomarkers that differentiate acute breathlessness from its recovery and the common cardio-respiratory conditions that present with acute breathlessness.

CRP plays an important role in diagnosing breathlessness caused by an underlying bacterial pneumonia[5], as well as predicting mortality in patients with COPD [6]. BNP is routinely utilised in acute settings to support the diagnosis of acute heart failure [7].The European Society of Cardiology (ESC) recommends BNP threshold values of <100 pg/mL to rule out acute congestive cardiac failure and values > 500 pg/ml as diagnostic of acute exacerbations of heart failure [8].

The role of peripheral blood eosinophil count in airway inflammation was poorly understood up until the second half of the 19th century when Paul Ehrlich, a German physician and Nobel prize winner, introduced eosin in his technique for white cell differentiation in 1879[9] . Considerable advances in the field of airway inflammation and the role of eosinophils have taken place since [10-12]. More recently Bafadhel *et al* suggested that peripheral blood eosinophil count can be used to direct corticosteroid therapy during COPD exacerbations in single centre study [13].

For peer review only - http://bmjopen.bmj.com/site/about/guidelines.xhtml

Currently, blood biomarkers together with clinical, physiological and imaging parameters are used in diagnosing the cause of acute breathlessness. Blood biomarkers may be less specific as they originate far from the target organs of interest (the heart and the lungs in cardio-respiratory disease). Sputum, although potentially a more definitive lung specific matrix, is comparatively difficult to obtain particularly in acutely unwell patients, limiting its use in acute disease and highlighting the need for better biomarkers. Ideally these biomarkers would have the following characteristics, (i) they would originate from the target organ of interest, (ii) they would significantly add value to conventional risk scoring and diagnostic algorithms in acute breathlessness, (iii) they would be minimally invasive and suitable for rapid point of care diagnosis in emergency rooms and acute admissions units (iv) they would have diagnostic value in patients with multifactorial acute breathlessness.

Exhaled breath contains thousands of volatile organic compounds (VOC) that reflect biological processes occurring in the host both locally in the airways and systematically offering the potential to develop more effective biomarkers in acutely breathless patients (**Figure 1**).

The proposed program of research will use a combination of offline and online technologies to identify and evaluate the diagnostic and prognostic value of VOC in patients with acute cardio-respiratory related breathlessness (**Figure 2**).

Exhaled breath analysis of volatile chemicals offers the potential to develop more effective biomarkers in acutely breathless patients. The use of breath analysis for disease diagnostics dates back to ancient Greeks where physicians used exhaled breath to diagnose different diseases. Breath odours allow correct associations to certain diseases. For example, the sweet smell of diabetic ketoacidosis, the fishy smell of breath associated to liver illness, the urine-like odour of kidney disease and the smell of the breath of patients with lung abscesses, caused by the proliferation of anaerobic bacteria [14-17].

For peer review only - http://bmjopen.bmj.com/site/about/guidelines.xhtml

More recently, exhaled breath analysis has demonstrated early proof of concept in the diagnosis of acute heart failure, and ventilator associated pneumonia[18] . The validity of breath analysis has also been demonstrated in breathless children[19]. This population is likely to prefer breath-based tests, as these are minimally invasive. Importantly, a variety of point of care sensors are now available to evaluate potential exhaled breath biomarkers in emergency care settings.

A study by Van Berkel et al demonstrated the ability to distinguish COPD subjects from controls solely based on the presence of VOCs in breath, suggesting that analysis of VOC might be highly relevant for diagnosis of COPD [20]. This established the basis of further studies of VOC in COPD [21-25].recommending larger studies for validation.

Several other studies found that VOC profiling in diagnosing asthma is potentially feasible [26-32]. This however has been done in relatively small numbers in stable disease.

Despite the novelty of non-invasive sampling technology and the growing interest in exhaled breath analysis there remains a disappointing level of comparability across studies due to the lack of standardisation and appropriate data analysis methods. A recent systemic review by Anders Christiansen *et al* compared eleven publications reporting very heterogeneous designs, methods, patient group sizes, data analytics and, consequently, quite varying results [33].

To our knowledge, no other large studies exploring the use of breath biomarkers in profiling acute breathlessness have been completed. Several studies have explored the use of electronic nose (eNose) in stable disease with good discriminatory power in COPD [34], Pneumonia [35] and heart failure[36] with relatively small sample size. While eNose has now been widely used in detecting various VOC patterns, GC-MS, a largely validated methodology, remains the gold standard technique for detecting VOCs in exhaled breath. The focus of the current research study will be to evaluate acutely breathless cardio-respiratory patients using a combination of 'discovery' and near-patient care breath sampling technologies.

For peer review only - http://bmjopen.bmj.com/site/about/guidelines.xhtml

Medical Research Council (MRC) and Engineering and Physical Sciences Research Council (EPSRC) have commissioned a series of molecular pathology nodes aimed at developing molecular signatures relevant to disease diagnosis and progression. This was triggered by the clear need for alliance between academic institutions, industry and NHS partners to enhance the benefits of stratified medicine for patients[37, 38].

University of Leicester and Loughborough University were awarded a joint molecular pathology node East Midlands Breathomics Pathology Node (EMBER) which this study forms a key part of.

2. Methods and Analysis

2.1. Study design

A prospective real world observational study across three acute admissions units within Leicestershire (two adult admissions units and one children's assessment unit). The acute units routinely assess and treat cardio-respiratory admissions due to breathlessness in adults and children.

Participants with self-reported acute breathlessness, either requiring admission or a change in baseline treatment, will be screened for the study. Informed consent will be obtained in all participants following a clinical review by a senior decision maker within 24 hours of acute admission (**Figure 3**).

2.2. Objectives

2.2.1. Primary objective

- To evaluate the sensitivity, specificity, positive and negative predictive value of exhaled breath VOC biomarkers to differentiate acute breathlessness in cardio-respiratory patients.

2.2.2. Secondary objectives:

- To replicate selected breath VOC biomarkers identified in acute breathlessness.

For peer review only - http://bmjopen.bmj.com/site/about/guidelines.xhtml

- To discover and replicate breath VOC biomarkers that differentiate the common cardio- respiratory conditions that cause acute breathlessness, specifically (i) acute heart failure, (ii) community acquired pneumonia, (iii) adult exacerbations of asthma and COPD and age-matched adults that do not have cardio-respiratory disease or breathlessness.
- To quantify the level of clinical uncertainty in the primary diagnosis using a 100 mm visual analogue scale and independent clinical adjudication of case notes blinded to the following blood biomarkers (i) CRP (ii) BNP (iii) Troponin-I (iv) blood eosinophils, but not clinical history and acute presentation nor chest x-ray imaging. Potential discriminatory breath VOC biomarkers will be adjusted for clinical uncertainly in statistical models.
- To identify and replicate exhaled breath VOC biomarkers in school age children treated in hospital for severe asthma attacks and compare these to age-matched healthy controls.

2.2.3. Exploratory end points (where applicable):

- To evaluate the dynamic profile of selected breath VOC between the acute state and the recovery state post exacerbation.
- To evaluate the relationship between exhaled VOC biomarkers and clinical outcomes including (i) hospital readmission at 30 and 60 days post event (ii) all-cause mortality over a 2 year period post admission.
- To evaluate the relationship between breath VOC biomarkers and functional measures e.g. physical performance and activity
- To explore potential breath VOC biomarkers of multifactorial acute breathlessness
- To evaluate the relationship between diet, lifestyle and environment upon breath VOC biomarkers

2.3. Sample size estimation

For peer review only - http://bmjopen.bmj.com/site/about/guidelines.xhtml

Preliminary data was utilised to conduct sample size estimates from a cohort of acutely breathless patients admitted to acute admissions units over a 6 month period (February 2017 to August 2017). Hundred and twelve adult participants (asthma 46, community acquired pneumonia 26, COPD 22) and eighteen healthy controls were utilised for the analysis.

A panel of ten pre-specified aldehydes, based on literature search [31, 39, 40], were extracted from breath using gas chromatography-mass spectrometry (GC-MS). The aldehydes were normalised to a common internal standard and were not background-subtracted.

A closed formula from Hsieh *et al*[41], relating sample size to observable effect size, was used to calculate sample size from logistic regression models of the ten aldehydes with acute breathlessness as the outcome measure. The sample size estimates are also relevant to acute class comparisons vs. the sum of other acute classes.

Based upon the sample size estimates we would have an 80% power, with a type 1 error rate of 5%, to detect an odds ratio of association of 1.2 between two disease classes with 55 patients per class. Given the fact that study seeks to discover and replicate breath VOC amongst five adult disease classes (community acquired pneumonia, heart failure, COPD, asthma and healthy aged matched subjects) we would require 110 adult patients per class – 550 patients across the program to achieve these aims.

The closed formulae by Tihaki *et al*,[42] were also utilised to understand the discriminatory power that the samples sizes above would provide with respect to biomarker sensitivity and specificity; The following assumptions were made:

- That a sensitivity of 80 % with a precision of 5% would provide a useful biomarker capable of ‘ruling out’ an acute class. The same target was applied to specificity.
- We assume a prevalence of acute breathlessness of 80% as the recruitment campaign uses acute breathlessness as the initial stratification tool for recruitment and 1:5 patients recruited will be non-breathless healthy controls

For peer review only - http://bmjopen.bmj.com/site/about/guidelines.xhtml

- We aim to balance group sizes across classes equally

For a type 1 error rate of 0.05 and a 95% confidence interval

$N_{sensitivity}$= 307

$N_{specificity}$= 1,230

For a type 1 error rate of 0.05 and a 90% confidence interval

$N_{sensitivity}$= 218

$N_{specificity}$ = 871

For a type 1 error rate of 0.05 and an 85% confidence interval

$N_{sensitivity}$ = 166

$N_{specificity}$ = 664

For a type 1 error rate of 0.05 and an 80% confidence interval

$N_{sensitivity}$ = 131

$N_{specificity}$ = 524

Therefore, we are powered to identify sensitive biomarkers (≥ 80%) of acute breathlessness with a maximum marginal error in the estimate for sensitivity not exceeding 5% with 95% confidence. Similarly, we are powered to identify specific biomarkers (≥ 80%) of acute breathlessness with a maximum marginal error in the estimate for specificity not exceeding 5% with 80% confidence.

For the primary analysis the outcome will be treated as a nominal variable with levels (i) acute heart failure (ii) community acquired pneumonia (iii) adult exacerbations of asthma and COPD and (iv) acute exacerbations in school age children treated in hospital for severe asthma attacks.

The relationship between the primary outcome and the exhaled breath VOC biomarkers will be modelled using multinomial logistic regression. In addition to metabolomics markers the following independent variables will be included in the model: clinical uncertainty score on a 100 mm VAS scale, age, and a

For peer review only

For peer review only - http://bmjopen.bmj.com/site/about/guidelines.xhtml

validated co morbidity score (the Charlson comorbidity score)[43, 44].

Receiver operator analyses will be utilised to generate ROC curves for individual and multiple panels VOC predictors in the primary analysis.

To understand the dynamic profile of breath biomarkers during (i) the acute state and (ii) in the chronic state up to 6 months post exacerbation, a repeated measures model with a random intercept and random effect for time will be fitted, the random effects will be fitted for each patient. For the repeated measures mixed model an unstructured covariance will be assumed. To evaluate the relationship between breath biomarkers and hospital readmission at 30 and 60 days Cox proportional hazards and frailty models will be utilised [45]. Analysis of Multivariate Survival Data, [CITE] competing risk models and joint models will be fitted [46]. Relationship between death and breath biomarkers will be evaluated using a logistic regression model. Changes in outcome measures will be measured appropriately for each variable (e.g. paired t-test, Mann-Whitney, repeated measures analysis).Tables of descriptive statistics will be compiled for all key variables

All analysis will be performed using R 3.5.0 https://www.r-project.org/.

2.4. Discovery and Replication studies

Specific indicator conditions have been selected for targeted recruitment according to their high prevalence and unmet need, their high morbidity and mortality and the need to develop better diagnostic and prognostic algorithms in acute care pathways.

The indicator diagnoses of interest are (i) exacerbations of adult asthma and COPD, (ii) community acquired pneumonia (iii) acute heart Failure and (iv) exacerbation in school age children treated in hospital for severe asthma attacks.

For peer review only - http://bmjopen.bmj.com/site/about/guidelines.xhtml

Patient level clinico-pathological and outcome data (spanning the entire acute pathway) will be collected in parallel to breath sampling. In addition, breath samples will be acquired in the stable state post exacerbation (**Figure 3**).

Age matched healthy volunteers will be recruited where possible at separate visits. For the purposes of this study, healthy volunteers will be defined as participants who have no prior history of asthma, COPD, heart failure and have not been admitted to hospital with community acquired pneumonia within 6 weeks of the baseline study visit. For acute admission the study team will approach the spouse, parent or sibling of the index case and seek informed consent for study assessments. All healthy subjects will undergo two assessments separated by a duration of 8-16 weeks to match the acute and recovery time points elapsed in their index case/partner/spouse/sibling/child. Additional healthy volunteers will be identified from local recruitment databases and via advertising

2.4.1. Discovery Phase (Project months 1-24):

The aim of the discovery phase is to discover putative discriminatory breath VOC, using both offline and online technologies.

Pre-planned recruitment of acutely breathless patients will be enriched into the following disease strata following senior clinical decision maker assessment and within 24 hours of acute admission.

Acute adult heart failure (n=55), adult community acquired pneumonia (n=55), adult exacerbations of asthma (n=55) and COPD (n=55), acute severe asthma attacks in school age children (n=50).

Additional age matched healthy volunteers (n=55 adults and 50 children) will be identified as a non-disease reference group **(Table 1).**

2.4.2. Replication Phase (years 3-4)

For peer review only

For peer review only - http://bmjopen.bmj.com/site/about/guidelines.xhtml

The aim of the replication phase is to replicate putative discriminatory breath VOC /VOC signatures identified in the discovery phase.

Similar to the discovery phase, recruitment of acutely breathless patients will be enriched into the following disease strata following senior clinical decision maker assessment and within 24 hours of acute admission.

Acute adult heart failure (n=55), adult community acquired pneumonia (n=55), adult exacerbations of asthma (n=55) and COPD (n=55), acute severe asthma attacks in school age children (n=25) **(Table 1)**. Additional age matched healthy volunteers (n=55 adults and 25 children) will be identified as a non-disease reference group.

Total combined sample size of the discovery and replication phases = 700 participants

**Disease Category**	**Discovery**	**Replication**
Acute Adult Asthma	**55**	**55**
Acute COPD	**55**	**55**
Acute Heart Failure	**55**	**55**
Community Acquired Pneumonia	**55**	**55**
Adult healthy volunteers	**55**	**55**
Acute paediatrics Asthma	**50**	**25**
Paediatrics healthy volunteers	**50**	**25**
**Total sample**	**375**	**325**

Table (1): Table summarising recruitment targets for both adult and paediatric groups.

2.5. Schedule of assessments

A schedule of acute assessments is outlined below and aligns to the movement of acute patients through the clinical care pathway and the overall aim of developing a complete phenotypic picture of acutely breathless patients.

2.5.1. Defining acute breathlessness

At presentation (within 24 hours of admission) to one of three acute admissions units potentially eligible patients will be identified following confirmation of acute breathlessness, identified as (i) patient defined acute breathlessness and/or (ii) 1 unit increase above patient reported baseline in the extended medical

For peer review only - http://bmjopen.bmj.com/site/about/guidelines.xhtml

research council (eMRC) dyspnoea score [47, 48] and at least one of the indicator diagnoses identified as the primary clinical diagnosis by a senior clinical decision maker.

2.5.2 Informed consent

Patients meeting the pre-specified definition of acute breathlessness will be approached for informed consent in to the breath VOC biomarker study. Only patients that are eligible to give full written informed consent will be recruited.

2.5.3 Collection of blood based pathology markers

Collection of the blood biomarkers CRP, BNP, Troponin-I and blood eosinophil count will be performed both acutely and following recovery, when not taken as part of clinical care pathway. These are currently used in profiling acutely breathless patients in clinical practice (**Table2**).

Test	ANALYSER/METHOD	LOWER LIMIT OF DETECTION	UPPER LIMIT OF DETECTION
C-Reactive protein (CRP)	Siemens Advia Chemistry XPT, PEG enhanced immunoturbidimetric. Siemens Advia 1800, PEG enhanced immunoturbidimetric	5 mg/L	Diluted to result
B-type natriuretic peptide (BNP)	Siemens Advia Centaur XPT, two-site sandwich immunoassay using direct chemiluminescent technology	2.0 pg/mL	1445 pg/mL
Troponin-I	Abbott Architect i2000SR, three-site sandwich immunoassay using direct chemiluminescent technology (CMIA).	5.0 ng/L	50,000 ng/L

Table (2): Type of analyser and methodology used for blood biomarker calculation. The table outlines analyser make, methodology, upper and lower limits of detection as per the University Hospitals of Leicester NHS Foundation trust laboratory guidelines.

2.5.4 Breath VOC sampling

Offline breath sampling using GC-MS coupled with a standardised and CE marked breath sampler- ReCIVA®[49] and comprehensive two-dimensional gas chromatography-mass spectrometry, coupled with a standardised and CE marked breath sampler (ReCIVA® GCxGC-MS) will be performed. Gas chromatography is considered a gold standard technique in detecting volatile organic compounds and as

For peer review only - http://bmjopen.bmj.com/site/about/guidelines.xhtml

such its sampling will be prioritised. Additionally the following online technologies, proton transfer mass spectroscopy (PTR-MS), gas chromatography - ion mobility spectroscopy (GC-IMS) and atmospheric pressure chemical ionisation- mass spectrometry (APCI-MS) will be evaluated according to the sampling strategy outlined in section 3, **Figure 3 and Table 3**.

	COPD		Asthma		Pneumonia		Heart Failure		Healthy		Paediatrics	
Time point	1	2	1	2	1	2	1	2	1	2	1	2
Written informed consent	x		x		x		x		x		x	
**Volatile organic compound (VOC) sampling**												
ReCIVA- gas chromatography and mass spectrometry (GC-MS)	x	x	x	x	x	x	x	x	x	x	x	x
ReCIVA- comprehensive two-dimensional gas chromatography (GCxGC-MS)	x	x	x	x	x	x	x	x	x	x	x	x
Atmospheric pressure chemical ionisation- mass spectrometry (APCI-MS)	x	x	x	x	x	x	x	x	x	x	x	x
Proton transfer reaction mass spectrometry (PTRMS)		x		x		x		x	x	x	x	x
Gas chromatography - ion mobility spectrometry (GC-IMS)	x		x		x		x				x	x
**Pathology blood tests**												
Full blood count (including differential cell count)	x	x	x	x	x	x	x	x	x	x	x	x
Brain natriuretic peptide (BNP) [pg/mL]	x	x	x		x		x	x	x			
Troponin-I]ng/L]	x		x		x		x		x			
C-Reactive protein (CRP) [mg/L]	x	x	x	x	x	x	x	x	x	x	x	x
**Lung function tests**											x	x
Hand held forced oscillation technique (FOT)	x	x	x	x	x	x	x	x	x	x		
Fractional exhaled nitric oxide (FeNO) - Flow rate 50 [ml/s]			x	x							x	x
Spontaneous sputum sample	x	x	x	x	x	x	x	x	x	x		
Bio-banking (urine, serum, plasma. sputum supernatants & plugs)	x	x	x	x	x	x	x	x	x	x	x	x

Transthoracic echocardiography	x						x					

Table (3): Summary of baseline and follow up assessments. The table summarises key assessments carried out at different time points during the study. The participants may undertake any combination of the investigations listed at any of these time points.

2.5.5. Collection of additional samples for future biomarker campaigns

Collection of additional biomarkers for future biomarker discovery campaigns including (i) a urine sample, (ii) blood samples, up to 85mls for DNA, RNA, plasma and serum and peripheral blood cell flow cytometry in selected subjects and (iii) spontaneous or induced sputum samples (plugs and supernatants) will be carried out (**Table 3**).

All samples will be collected at time point 1 and at 2 (**Figure 3**). The additional samples will be used for future omics analyses, these may include detailed analysis of the metagenome in sputum and proteomics applied to urine and serum samples.

2.5.6. Physiological characterisation

Physiological measures of lung function will be performed in acutely ill participants and at recovery including, (i) Hand held forced oscillation technique (FOT): an easily accessible measure of lung function. Patients favour this to spirometry as it is effort independent, unlike spirometry and requires less than a minute of quiet tidal breathing to obtain triplicate high quality measurements [50], (ii) Fractional exhaled nitric oxide (FeNO): A measure of airway inflammation in asthmatic patients[51, 52] (iii) Echocardiography: Two dimensional transthoracic echocardiography will be performed in heart failure and COPD patients using an iE 33 system with S5-1 transducer (frequency transmitted 1.7 MHz, received 3.4 MHz; Philips Medical Systems, Best, The Netherlands). Standard techniques as per American Society of

For peer review only

For peer review only - http://bmjopen.bmj.com/site/about/guidelines.xhtml

Echocardiography guidelines (ASE)[53] were used to acquire 2D, colour and Doppler images in conventional parasternal long-axis, short-axis and apical 4-, 2- and 3-chamber views. Left ventricular ejection fraction (LVEF) was calculated using the biplane method of discs formula (Simpson's rule) to derive left ventricular volume indices.

All participants are encouraged to report any testing related discomfort or concerns to the research team to terminate the sampling process.

2.5.7 Recovery follow up

- Patient recovery will be defined as:
  - (i) Patient reported recovery from the acute exacerbation spell and back to their baseline extended MRC score or clinician defined recovery from the acute exacerbation spell

  and

  - (ii) At least 6 weeks post exacerbation event (up to 6 months).

Patients that re admit to hospital between visits 1 and 2, can have additional visit 1 assessments. Visit 2 will be taken as recovery following the subsequent admission. If a patient is admitted to hospital after visit 2 then they will be eligible to be recruited as a new study participant.

The schedule of assessments at the recovery visit is outlined (**Table 3).**

2.6. Clinical Adjudication:

In an effort to reduce data variability and minimise bias, an independent panel consisting of two senior acute clinicians (SS & NG) will review all pertinent clinical and diagnostic source documentation, whilst blinded to admission blood biomarkers and clinical diagnosis.

All acutely breathless adult patients' notes will be sequentially divided for adjudication. The panel will independently determine the primary diagnosis of highest probability from a list of the four potential acute indicator diagnoses in section 2.4 and mark their level of clinical certainty on a 100 mm visual analogue scale (VAS scale). The panel members will be able to review imaging, electrocardiograms (ECGs), and other relevant information but not admission blood based pathology tests.

In a subset of patients adjudication will be validated by separate panel member to ensure between observer agreement using Bland-Altman analysis and inter-rater agreement of the primary diagnosis using Kohen's

For peer review only - http://bmjopen.bmj.com/site/about/guidelines.xhtml

kappa via repeated evaluation of a subset of cases (see statistical methods section 2.3).

2.7. Clinical Informatics:

Clinical data collection will be undertaken using a securely-hosted bespoke database system (ADD) developed within the NIHR Leicester Biomedical Research Centre – Respiratory (BRC). The system links acute admission episodes to hospital pathology records; historical respiratory physiology tests; and demographic information. The system provides functionality to validate data entry; manually verify records and highlight incomplete records. A custom VOC 'module' has been be created to support data collection within the study visits (1 and 2), and standardise diagnoses and medications through the use of clinical ontologies as well as linking hospital records/tests to patient visits.

Non-ADD based clinical data (e.g. hospital admissions, re-admissions and mortality) will be extracted from the hospital data warehouse using identifiable patient identifiers, and subsequently pseudonymised prior to integration.

An informatics pipeline will be created to facilitate the transfer of chemo-metric data from remote computers to the data repository. This will include tools to (i) enforce the correct labelling of data sets (e.g. study number, visit, type/source of sample) prior to automated validated transfer to the repository; (ii) record information about the sample process; (iii) search and extract data sets from the repository for subsequent analysis. Prior to analysis, clinical and chemo-metric data will be integrated using the study number and any potentially identifiable information will be removed.

3. Breath profiling

The technologies utilised in the VOC study during discovery and replication phases are:

For peer review only - http://bmjopen.bmj.com/site/about/guidelines.xhtml

Offline technologies

- ReCIVA+ GC-MS
- ReCIVA + GC x GC-MS
-

Online technologies

- GC-IMS
- PTR-MS
- APCI-MS

Offline technologies will underpin the discovery analyses owing to their ability to identify chemical identity and their recognition as the analytical gold standard in exhaled breath VOC analysis [54]. In contrast online technologies will be utilised for VOC biomarker replication and at the recovery visits owing to their portability and potential for future point of care testing. **(Figure 4)**. A brief description of the core VOC platforms is provided below

A CE marked breath sampling device (ReCIVA®) developed by Owlstone Medical, will be used to sample breath onto two adsorbent Tenax tubes. Participants will be asked to breathe through the ReCIVA face mask for a maximum of 300 seconds, aiming for collection of $\geq$ 80% of the target sample volume of 1 litre, after which the Tenax tubes will be transferred to the laboratory for analysis. This effectively allows de-coupling of the breath sampling from the breath sensor and analysis platforms in selected patients that are not able to mobilise to a real time breath sampling device. The Owlstone ReCIVA sampler will be utilised in breath collection for offline technologies namely GC-MS and GCxGC-MS. The ReCIVA sampler is capable of entraining oxygen and is therefore suitable for patients with mild respiratory failure requiring low flow rates of oxygen to maintain target oxygen saturations [49].

3.1. Gas chromatography and mass spectroscopy (GC-MS): is a commonly applied methodology used to accurately measure trace gases in complex mixtures such as exhaled air [54]. Pre-concentrating breath volatiles by various means and subsequent analysis constitute a reliable and sensitive method for VOC

For peer review only

analysis [55]. Despite its high sensitivity, it is however, a time consuming technique and carries a risk of contamination at the pre-concentration step. It is also not suitable for online and multiple measurements limiting its use as a point-of-care testing technology for VOC [56].

3.2. Comprehensive two-dimensional gas chromatography-mass spectrometry (GCxGC-MS): an advanced analytical technique for the analysis of complex organic matrices; its main advantage is the unparalleled separation power it affords over conventional one-dimensional chromatographic techniques [57]. Previous research, albeit sparse, has demonstrated the potential of GCxGC-MS for breath analysis with the number of VOC detected exceeding those detected by conventional GC-MS [58, 59]. GCxGC-MS of breath metabolites has been used for the identification of biomarkers related to glucose metabolism [60, 61], tuberculosis [62] and radiation response [63]. This has generated interest within the breath research community, however, such studies were conducted on a small scale (<50 patients) and involved the use of expensive detectors and modulators. Method development and analysis of the data-rich GCxGC chromatograms, however, can be time-consuming and require specialist knowledge.

3.3. Proton-transfer-reaction mass spectrometry (PTRMS): a real time technique, capable of simultaneously measuring the evolution of multiple gas metabolites from a single breath. It has been used for the identification of potential useful VOC biomarkers for diagnosis of a variety of diseases: including various cancers[64-66], liver disease[67, 68] and respiratory disease[69]. It has several advantages in clinical settings, such as the speed of sampling, the instant result achieved and the lack of need for sample storage or shipping. However, owing to the lack of pre-concentration or chromatographic separation, sensitivity and definitive compound identification can be somewhat limited when compared to GC-MS.

Two breath sampling devices will be used. The first device is a Loccioni SOFIA GSI-S; the subject is required to exhale a single breath, five times (three if providing five samples proves too difficult) into a sterile mouthpiece connected to an electrostatic bacterial/viral filter whilst wearing a nose clip (all CE marked). Flow from the mouthpiece passes into a gas sampling interface capnograph (Loccioni GSI-S – CE marked) and real-time user feedback of flow is provided on screen, allowing the regulation of the

For peer review only - http://bmjopen.bmj.com/site/about/guidelines.xhtml

breath sampling rate. The gas sampling interface acts to simultaneously trigger the acquisition of the PTR-ToF-MS data and the exhaled breath travels through the capnograph down a heated sample line into the ion source of the PTR-ToF-MS

The second breath sampling device is a ReCIVA breath sampler (Owlstone) with one of the adsorbent Tenax tubes replaced with an outlet tube adapted for online sampling. The exhaled breath is transferred to the PTR-ToF-MS via a heated transfer line connected to the outlet tube, continuously drawn at a constant flow rate by the PTR-ToF-MS. The online adaptation of the consumable adsorbent tube does not affect the CE mark of the ReCIVA sampling device.

Once the breath sample reaches the PTR-ToF-MS, via either breath sampler, the breath mixes with protonated water (H_3O^+) inducing proton transfer to the target volatile organic compounds (VOCs) present, resulting in their ionisation. Sample ions are then guided into the time of flight mass spectrometer and mass spectra, showing the abundance and mass of the VOCs present, are collected throughout the exhalation. Following sampling, mouthpieces, filters and nose clips are disposed of and all patient contacted surfaces wiped down with antiseptic cleaning wipes in preparation for the next patient.

3.4. Gas chromatography- ion mobility spectrometry (GC-IMS) (B&S Analytiks): Allows the detection of volatile organic compounds down to ultra-trace level (ng/L - to pg/L - range). For years, IMS has been used to discover potential discriminatory breath VOC in lung cancer [70, 71], COPD[72, 73] and asthma[73]. Sampling takes place using a Spiroscout spirometer. The patients exhale through a disposable mouth piece connected to a Teflon tube. A piezoelectric pressure sensor is used to monitor the breathing profile, this opens the sampling valve at the appropriate point in the breath profile to collect end-tidal breath in a sample loop of 10 mL volume. After filling this loop, the collected sample air is then transferred to a multicapillary column for a chromatographic separation, which is achieved in 12 min. The

For peer review only - http://bmjopen.bmj.com/site/about/guidelines.xhtml

separated molecules are then transferred into the IMS, ionised and then separated according to their mobility in a weak electric field.

The technology's multiple advantages of ultra-sensitivity, portability, online sampling and short analysis time (typical analysis time of 10 minutes) with real time detection, brings a promise to provide immediate and potentially reliable results for point of care breath diagnostics. Another concept with IMS devices is that once the required breath signatures have been discovered using GC-MS, IMS offers the potential to be 'tuned' for selective detection of VOC.

3.5. Atmospheric pressure chemical ionisation- mass spectrometry (APCI-MS) semiportable compact version (Advion): is one of less sensitive but more affordable versions of mass spectrometers released to the commercial market in recent years. The device uses APCI to produce ions. Although the most common use of APCI-MS systems is the detection in liquid chromatography applications, the technique has proven to be a valuable tool for direct measurement of VOC in air[74, 75] food[76, 77] and breath[78, 79]. Recently, the technique has shown potential for online, real time profiling of pseudo-metabolites in exhaled breath [80] with sensitivity comparable with other techniques. By combining miniaturised MS technology with APCI techniques, adequate quality of on-site, real time measurements with minimal or no sample preparation requirement can be provided. This is a desirable outcome as it overcomes main limitation of using standard breath analysis method in clinical setting, which is a need for breath sample [illegible]

There remains an overall lack of standardisation and rigour across these technologies which hindered previous advancements in breath discovery; something we intend to minimize.

4. Chemometric processing and data analysis:

GC-MS breath data will be aligned, deconvoluted and the features for each participant will be extracted. The extracted features will be grouped and classified by retention index and mass spectrum. The

For peer review only - http://bmjopen.bmj.com/site/about/guidelines.xhtml

registered and aligned data will be linked to participant meta-data to generate a breath matrix. Data handling and analysis will be performed by a senior statistician.

The breath matrix is a $n \times p$ matrix where n is the number of subjects and p is the number of VOC. The breath matrix is high dimensional with $p \gg n$ and many potentially correlated VOC. In view of this, we will employ sparse partial least squares discriminant analysis (sPLS-DA)[81] to investigate which of the VOC can identify breathlessness. We will also investigate which of the VOC can discriminate between the different disease states including acute exacerbations of asthma and COPD and Pneumonia. In addition to the supervised methods, unsupervised methods will be explored, specifically sparse principle component analysis (sPCA)[82].

Extracted VOC will also be investigated. Relationships between VOC and patient reported acute breathlessness will be analysed using logistic regression model. VOC associated with patient associated acute breathlessness will be incorporated into multinomial logistic regression models in conjunction with CRP, BNP, blood eosinophils and Troponin-I, pathology biomarkers currently in use for diagnosing undifferentiated breathlessness. In addition to the conventional binary and multinomial logistic regression models, regression models [83]..

For peer review only

5. Ethics and dissemination:

The study has obtained full ethical approval from the London South East Research ethics Committee, REC reference 16/LO/1747. IRAS project ID 198921. Publications will be prepared according to the MRC-EMBER consortium agreement and the University of Leicester publications policy. All intended publications will be submitted to the EMBER executive board for review and comments within 60 days of journal submission. Authorship will be according to contribution and internationally recognised guidance on journal authorship.

6. Study dates: 01/2/2017 – 30/10/2020

For peer review only - http://bmjopen.bmj.com/site/about/guidelines.xhtml

7. Authors' contributions:

S.S, C.E.B, N.Gr, P.Th and P.Mo conceived the study, obtained funding, wrote the study protocol, obtained ethical and MHRA approvals for the study and coordinated the deployment of analytical testing methods for breath analysis. W.I took the lead in writing the manuscript with support from S.S. Planning and recruitment of adult participants was carried out by W.I, S.Jo, B.Pa, A.Aw, R.Ph, G.Fo, A.Yo, R. J. R and C.Wh. Paediatrics study design was conceived by E.Ga and C.Be and participants recruited by T. Mc and C. Fo. Analytical chemistry team formed of M.Wi, R.Co, D.Sa, D.Ru and L.Br expertly handled all the breath samples and planned an analysis structure. M.Ri, a senior statistician, constructed a statistics and data analysis plan in conjunction with SS. Bioinformatics pipeline and electronic CRFs developed by R.Fr and B.Zh. All authors, including R.Pe, H. Bh, B.Ha, A. Si, K. Ry, H. Pa, T. Su, L. L Ng, contributed to the study design and study protocol.

8. Protocol version: Version 4, 1st April 2018

9. Public and patient involvement:

A series of consultations have taken place with our patient involvement team within the NIHR Biomedical Research Centre (Respiratory Theme) and across the wider BRC PPI group. Representations from the paediatrics team were also present. This group was sent copies of the participant documentation for review and discussion. Various revisions have been made following on from these discussions.

10. Funding statement:

Research funding was provided by the MRC and EPSRC Stratified Medicine Grant for Molecular Pathology Nodes (Grant No. MR/N005880/1). The research was supported by the National Institute for Health Research (NIHR) Leicester Biomedical Research Centre and NIHR, Leicester Clinical Research

For peer review only

For peer review only - http://bmjopen.bmj.com/site/about/guidelines.xhtml

Facility and the Midlands Asthma and Allergy Research Association (MAARA) to whom we are extremely grateful. The authors would like to acknowledge the invaluable efforts of the research nurses responsible for the in-clinic sample collection as well as the input from the wider EMBER consortium (Members list can be found at: https://ember.le.ac.uk/web). The views expressed are those of the author(s) and not necessarily those of the NHS and NIHR or the Department of Health

11. Competing interests: SS has performed advisory services for Owlstone Medical.

Figure legends:

Figure (1) Relationship between lung proximity and degree of invasiveness of different lung matrices. The figure plots the level of invasiveness of various lung matrices in relation to their proximity to the lung. Given their pathological relevance, the degree of invasiveness of bronchoalveolar lavage (BAL) and lung biopsy makes them less favourable in diagnosing respiratory diseases.

For peer review only

For peer review only - http://bmjopen.bmj.com/site/about/guidelines.xhtml

Figure (2) Multi-instrument use in breath sampling. Figure illustrates the various combinations of offline and online devices used in breath sampling and the relevant pros and cons. Offline and online technologies are used for the discovery and validation phases of the study respectively.

Figure (3) Study flow chart. Figure outlines the patient journey from admission through to discharge and follow up. Participants with self-reported acute breathlessness presenting to University Hospitals of Leicester are recruited within 24 hours following a senior decision maker review. Breath sampling is carried out on the first visit, at recruitment, and the second visit, up to 6 months after discharge. Patients are admitted through the standard operational emergency medical streaming and care pathways at the University Hospitals of Leicester NHS Trust. Early outcomes (hospital readmission) are measured at 30 and 60 days and late outcomes (mortality) including respiratory and all-cause mortality are measured at 2 years. Assessments carried out at each time point are summarised in **Table 1**.

Figure (4) Multi-instrument use in breath sampling. Operational space of the analytical technologies used in EMBER for the analysis of volatile organic compounds (VOC) in exhaled breath, including proton transfer reaction-mass spectrometry (PTR-MS), atmospheric pressure chemical ionisation-mass spectrometry (CMS), gas chromatography-ion mobility spectrometry (GC-IMS), gas chromatography-mass spectrometry (GC-MS) and two-dimensional gas chromatography-mass spectrometry (GCxGC). Comparing the typical molar mass range detectable; selectivity in detection owing to the type of ionisation involved and the proton affinity of analytes; and the inclusion of a chromatographic separation affecting total time of analysis. The online technologies involving chemical ionisation (PTR-MS, CMS and GC-IMS) can be used in-clinic owing to short analysis times, but only detect lower molar mass molecules with a proton affinity higher than 697 KJ mol-1. Offline chromatographic techniques (GC-MS and GCxGC) detect a wider range of compounds independent of proton affinity; however, the techniques have longer analysis times and involve sample transportation and storage.

For peer review only - http://bmjopen.bmj.com/site/about/guidelines.xhtml

8. References

1. Stephenson, J.J., et al., *Clinical and economic burden of dyspnea and other COPD symptoms in a managed care setting.* Int J Chron Obstruct Pulmon Dis, 2017. **12**: p. 1947-1959.
2. David Ponka, M.K., *Top differential diagnoses in family medicine Dyspnoea Can Fam Physician.* Canadian Family Physician, 2008.

For peer review only - http://bmjopen.bmj.com/site/about/guidelines.xhtml

3. Figarska, S.M., H.M. Boezen, and J.M. Vonk, *Dyspnea severity, changes in dyspnea status and mortality in the general population: the Vlagtwedde/Vlaardingen study.* European Journal of Epidemiology, 2012. **27**(11): p. 867-876.

4. Parshall, M.B., et al., *An Official American Thoracic Society Statement: Update on the Mechanisms, Assessment, and Management of Dyspnea.* American Journal of Respiratory and Critical Care Medicine, 2012. **185**(4): p. 435-452.

5. Lim, W.S., et al., *BTS guidelines for the management of community acquired pneumonia in adults: update 2009.* Thorax, 2009. **64 Suppl 3**: p. iii1-55.

6. Leuzzi, G., et al., *C-reactive protein level predicts mortality in COPD: a systematic review and meta-analysis.* Eur Respir Rev, 2017. **26**(143).

7. Moe, G.W., *B-type natriuretic peptide in heart failure.* Curr Opin Cardiol, 2006. **21**(3): p. 208-14.

8. Ponikowski, P., et al., *2016 ESC Guidelines for the diagnosis and treatment of acute and chronic heart failure: The Task Force for the diagnosis and treatment of acute and chronic heart failure of the European Society of Cardiology (ESC)Developed with the special contribution of the Heart Failure Association (HFA) of the ESC.* Eur Heart J, 2016. **37**(27): p. 2129-2200.

9. Alex Sakula, *Charcot leyden crystals and crushmann spirals.* Thorax, 1986.

10. Calhoun, W.J., J. Sedgwick, and W.W. Busse, *The role of eosinophils in the pathophysiology of asthma.* Ann N Y Acad Sci, 1991. **629**: p. 62-72.

11. Pavord, I.D., *Eosinophilic phenotypes of airway disease.* Ann Am Thorac Soc, 2013. **10 Suppl**: p. S143-9.

12. Tashkin, D.P. and M.E. Wechsler, *Role of eosinophils in airway inflammation of chronic obstructive pulmonary disease.* International Journal of Chronic Obstructive Pulmonary Disease, 2018. **13**: p. 335-349.

13. Bafadhel, M., et al., *Blood Eosinophils to Direct Corticosteroid Treatment of Exacerbations of Chronic Obstructive Pulmonary Disease: A Randomized Placebo-Controlled Trial.* American Journal of Respiratory and Critical Care Medicine, 2012. **186**(1): p. 48-55.

14. Bedossa, P. and K. Patel, *Biopsy and Noninvasive Methods to Assess Progression of Nonalcoholic Fatty Liver Disease.* Gastroenterology, 2016. **150**(8): p. 1811-1822 e4.

15. Fens, N., et al., *Exhaled breath analysis by electronic nose in airways disease. Established issues and key questions.* Clin Exp Allergy, 2013. **43**(7): p. 705-15.

16. Probert, C.S., et al., *Volatile organic compounds as diagnostic biomarkers in gastrointestinal and liver diseases.* J Gastrointestin Liver Dis, 2009. **18**(3): p. 337-43.

17. Schubert, J.K., et al., *Breath analysis in critically ill patients: potential and limitations.* Expert Rev Mol Diagn, 2004. **4**(5): p. 619-29.

18. Fowler, S.J., et al., *Surveillance for lower airway pathogens in mechanically ventilated patients by metabolomic analysis of exhaled breath: a case-control study.* Thorax, 2015. **70**(4): p. 320-5.

19. Florian Gahleitner, C.G.-H., Caroline S Beardsmore, Hitesh C Pandya, CL Paul Thomas, *Metabolomics pilot study to identify VOC of childhood asthma in exhaled breath.* Bioanalysis, 2013.

20. Van Berkel, J.J., et al., *A profile of volatile organic compounds in breath discriminates COPD patients from controls.* Respir Med, 2010. **104**(4): p. 557-63.

21. Basanta, M., et al., *Exhaled volatile organic compounds for phenotyping chronic obstructive pulmonary disease: a cross-sectional study.* Respir Res, 2012. **13**: p. 72.

22. Besa, V., et al., *Exhaled volatile organic compounds discriminate patients with chronic obstructive pulmonary disease from healthy subjects.* International Journal of Chronic Obstructive Pulmonary Disease, 2015. **10**: p. 399-406.

For peer review only

For peer review only - http://bmjopen.bmj.com/site/about/guidelines.xhtml

23. Hauschild, A.C., J.I. Baumbach, and J. Baumbach, *Integrated statistical learning of metabolic ion mobility spectrometry profiles for pulmonary disease identification.* Genet Mol Res, 2012. **11**(3): p. 2733-44.
24. Jareno-Esteban, J.J., et al., *Study of 5 Volatile Organic Compounds in Exhaled Breath in Chronic Obstructive Pulmonary Disease.* Archivos De Bronconeumologia, 2017. **53**(5): p. 251-256.
25. Paredi, P., et al., *Exhaled ethane, a marker of lipid peroxidation, is elevated in chronic obstructive pulmonary disease.* Am J Respir Crit Care Med, 2000. **162**(2 Pt 1): p. 369-73.
26. Caldeira, M., et al., *Profiling allergic asthma volatile metabolic patterns using a headspace-solid phase microextraction/gas chromatography based methodology.* J Chromatogr A, 2011. **1218**(24): p. 3771-80.
27. Caldeira, M., et al., *Allergic asthma exhaled breath metabolome: a challenge for comprehensive two-dimensional gas chromatography.* J Chromatogr A, 2012. **1254**: p. 87-97.
28. Dallinga, J.W., et al., *Volatile organic compounds in exhaled breath as a diagnostic tool for asthma in children.* Clin Exp Allergy, 2010. **40**(1): p. 68-76.
29. Dragonieri, S., et al., *An electronic nose in the discrimination of patients with asthma and controls.* J Allergy Clin Immunol, 2007. **120**(4): p. 856-62.
30. Fens, N., et al., *Exhaled breath profiling enables discrimination of chronic obstructive pulmonary disease and asthma.* Am J Respir Crit Care Med, 2009. **180**(11): p. 1076-82.
31. Ibrahim, B., et al., *Non-invasive phenotyping using exhaled volatile organic compounds in asthma.* Thorax, 2011. **66**(9): p. 804-9.
32. Montuschi, P., et al., *Diagnostic performance of an electronic nose, fractional exhaled nitric oxide, and lung function testing in asthma.* Chest, 2010. **137**(4): p. 790-6.
33. Christiansen, A., et al., *A systematic review of breath analysis and detection of volatile organic compounds in COPD.* J Breath Res, 2016. **10**(3): p. 034002.
34. de Vries, R., et al., *Integration of electronic nose technology with spirometry: validation of a new approach for exhaled breath analysis.* J Breath Res, 2015. **9**(4): p. 046001.
35. Shafiek, H., et al., *Using the Electronic Nose to Identify Airway Infection during COPD Exacerbations.* PLoS One, 2015. **10**(9): p. e0135199.
36. Finamore, P., et al., *Analysis of volatile organic compounds: an innovative approach to heart failure characterization in older patients.* J Breath Res, 2018. **12**(2): p. 026007.
37. *MRC Molecular Pathology, Review 20 Aug.*
38. Medical Research Council, *Molecular Pathology Nodes*. 2014: MRC website.
39. Corradi, M., et al., *Aldehydes in exhaled breath condensate of patients with chronic obstructive pulmonary disease.* Am J Respir Crit Care Med, 2003. **167**(10): p. 1380-6.
40. Larstad, M., et al., *Determination of malondialdehyde in breath condensate by high-performance liquid chromatography with fluorescence detection.* J Chromatogr B Analyt Technol Biomed Life Sci, 2002. **766**(1): p. 107-14.
41. F. Y. Hsieh, D.A.B., Michael D. Larsen,, *A simple method of sample size calculation for linear and logistic regression* Statis Med, 1998.
42. Hajian-Tilaki, K., *Sample size estimation in diagnostic test studies of biomedical informatics.* J Biomed Inform, 2014. **48**: p. 193-204.
43. Quan, H., et al., *Updating and validating the Charlson comorbidity index and score for risk adjustment in hospital discharge abstracts using data from 6 countries.* Am J Epidemiol, 2011. **173**(6): p. 676-82.
44. Roffman, C.E., J. Buchanan, and G.T. Allison, *Charlson Comorbidities Index.* J Physiother, 2016. **62**(3): p. 171.
45. Bjarnason, H. and P. Hougaard, *Fisher information for two gamma frailty bivariate Weibull models.* Lifetime Data Anal, 2000. **6**(1): p. 59-71.

For peer review only

For peer review only - http://bmjopen.bmj.com/site/about/guidelines.xhtml

46. Lawrence Gould, A., et al., *Joint modeling of survival and longitudinal non-survival data: current methods and issues. Report of the DIA Bayesian joint modeling working group.* Stat Med, 2015. **34**(14): p. 2181-95.
47. Steer, J., et al., *Dyspnoea severity and pneumonia as predictors of in-hospital mortality and early readmission in acute exacerbations of COPD.* Thorax, 2012. **67**(2): p. 117-21.
48. Tsimogianni, A.M., et al., *Predictors of Outcome After Exacerbation of Chronic Obstructive Pulmonary Disease.* Journal of General Internal Medicine, 2009. **24**(9): p. 1043-1048.
49. Kitchen, S., et al., *LATE-BREAKING ABSTRACT: Breathe free: Open source development of a breath sampler by a consortium of breath researchers.* European Respiratory Journal, 2015. **46**(suppl 59).
50. Oostveen, E., et al., *The forced oscillation technique in clinical practice: methodology, recommendations and future developments.* Eur Respir J, 2003. **22**(6): p. 1026-41.
51. Pendharkar, S. and S. Mehta, *The clinical significance of exhaled nitric oxide in asthma.* Canadian Respiratory Journal : Journal of the Canadian Thoracic Society, 2008. **15**(2): p. 99-106.
52. Yates, D.H., *Role of exhaled nitric oxide in asthma.* Immunol Cell Biol, 2001. **79**(2): p. 178-90.
53. Lang, R.M., et al., *Recommendations for chamber quantification: a report from the American Society of Echocardiography's Guidelines and Standards Committee and the Chamber Quantification Writing Group, developed in conjunction with the European Association of Echocardiography, a branch of the European Society of Cardiology.* J Am Soc Echocardiogr, 2005. **18**(12): p. 1440-63.
54. J. Throck Watson, O.D.S., *Introduction to Mass Spectrometry: Instrumentation, Applications, and Strategies for Data Interpretation, 4th Edition*. 2007: Wiley. 862.
55. Buszewski, B., et al., *Human exhaled air analytics: biomarkers of diseases.* Biomed Chromatogr, 2007. **21**(6): p. 553-66.
56. Amann, A., et al., *Applications of breath gas analysis in medicine.* International Journal of Mass Spectrometry, 2004. **239**(2-3): p. 227-233.
57. Almstetter, M.F., P.J. Oefner, and K. Dettmer, *Comprehensive two-dimensional gas chromatography in metabolomics.* Anal Bioanal Chem, 2012. **402**(6): p. 1993-2013.
58. Das, M.K., et al., *Investigation of Gender-Specific Exhaled Breath Volatome in Humans by GCxGC-TOF-MS.* Analytical Chemistry, 2014. **86**(2): p. 1229-1237.
59. Phillips, M., et al., *Detection of an extended human volatome with comprehensive two-dimensional gas chromatography time-of-flight mass spectrometry.* PLoS One, 2013. **8**(9): p. e75274.
60. Gruber, B., et al., *Vacuum ultraviolet absorption spectroscopy in combination with comprehensive two-dimensional gas chromatography for the monitoring of volatile organic compounds in breath gas: A feasibility study.* J Chromatogr A, 2016. **1464**: p. 141-6.
61. Gruber, B., et al., *Breath gas monitoring during a glucose challenge by a combined PTR-QMS/GCxGC-TOFMS approach for the verification of potential volatile biomarkers.* J Breath Res, 2016. **10**(3): p. 036003.
62. Mellors, T.R., et al., *A new method to evaluate macaque health using exhaled breath: A case study of M. tuberculosis in a BSL-3 setting.* J Appl Physiol (1985), 2017. **122**(3): p. 695-701.
63. Phillips, M., et al., *Breath biomarkers of whole-body gamma irradiation in the Gottingen minipig.* Health Phys, 2015. **108**(5): p. 538-46.
64. Bajtarevic, A., et al., *Noninvasive detection of lung cancer by analysis of exhaled breath.* BMC Cancer, 2009. **9**: p. 348.
65. Lavra, L., et al., *Investigation of VOCs associated with different characteristics of breast cancer cells.* Sci Rep, 2015. **5**: p. 13246.

For peer review only

For peer review only - http://bmjopen.bmj.com/site/about/guidelines.xhtml

66. Zhou, W., et al., *Exhaled breath online measurement for cervical cancer patients and healthy subjects by proton transfer reaction mass spectrometry.* Anal Bioanal Chem, 2017. **409**(23): p. 5603-5612.
67. Fernández del Río, R., et al., *Volatile Biomarkers in Breath Associated With Liver Cirrhosis — Comparisons of Pre- and Post-liver Transplant Breath Samples.* EBioMedicine, 2015. **2**(9): p. 1243-1250.
68. O'Hara, M.E., et al., *Limonene in exhaled breath is elevated in hepatic encephalopathy.* J Breath Res, 2016. **10**(4): p. 046010.
69. Tomasz Ligor, C.A., Anton Amann, Konrad Schwarz, Bogusaw Buszewski, Wojciech ebrowski,, *<Comparison of Proton Transfer Reaction-Mass Spectrometry and Gas Chromatography-Mass Spectrometry in Analysis of Breath Samples.pdf>.* Chem. Anal., 2009.
70. Darwiche, K., et al., *Bronchoscopically obtained volatile biomarkers in lung cancer.* Lung, 2011. **189**(6): p. 445-52.
71. Handa, H., et al., *Exhaled Breath Analysis for Lung Cancer Detection Using Ion Mobility Spectrometry.* PLoS ONE, 2014. **9**(12): p. e114555.
72. Bessa, V., et al., *Detection of volatile organic compounds (VOCs) in exhaled breath of patients with chronic obstructive pulmonary disease (COPD) by ion mobility spectrometry.* International Journal for Ion Mobility Spectrometry, 2011. **14**(1): p. 7-13.
73. Schivo, M., et al., *Mobile instrumentation platform to distinguish airway disorders.* Journal of breath research, 2013. **7**(1): p. 017113-017113.
74. Badjagbo, K., et al., *Direct atmospheric pressure chemical ionization-tandem mass spectrometry for the continuous real-time trace analysis of benzene, toluene, ethylbenzene, and xylenes in ambient air.* J Am Soc Mass Spectrom, 2009. **20**(5): p. 829-36.
75. Healy, R.M., et al., *A multi-year study of VOC emissions at a chemical waste disposal facility using mobile APCI-MS and LPCI-MS instruments.* Environ Pollut, 2018. **232**: p. 220-228.
76. Linforth, R. and A.J. Taylor, *Persistence of Volatile Compounds in the Breath after Their Consumption in Aqueous Solutions.* Journal of Agricultural and Food Chemistry, 2000. **48**(11): p. 5419-5423.
77. Perez-Hurtado, P., et al., *Direct analysis of volatile organic compounds in foods by headspace extraction atmospheric pressure chemical ionisation mass spectrometry.* Rapid Commun Mass Spectrom, 2017. **31**(22): p. 1947-1956.
78. Berchtold, C., L. Meier, and R. Zenobi, *Evaluation of extractive electrospray ionization and atmospheric pressure chemical ionization for the detection of narcotics in breath.* International Journal of Mass Spectrometry, 2011. **299**(2-3): p. 145-150.
79. Kapishon, V., et al., *Atmospheric pressure chemical ionization mass spectrometry of pyridine and isoprene: potential breath exposure and disease biomarkers.* J Breath Res, 2013. **7**(2): p. 026005.
80. Heaney, L.M., et al., *Real-time monitoring of exhaled volatiles using atmospheric pressure chemical ionization on a compact mass spectrometer.* Bioanalysis, 2016. **8**(13): p. 1325-36.
81. Lê Cao, K.-A., S. Boitard, and P. Besse, *Sparse PLS discriminant analysis: biologically relevant feature selection and graphical displays for multiclass problems.* BMC Bioinformatics, 2011. **12**: p. 253-253.
82. Zou, H., T. Hastie, and R. Tibshirani, *Sparse Principal Component Analysis.* Journal of Computational and Graphical Statistics, 2006. **15**(2): p. 265-286.
83. Zou H, H.T., *Regularization and Variable Selection via the Elastic Net.* Journal of the Royal Statistical Society, 2005.

For peer review only

For peer review only - http://bmjopen.bmj.com/site/about/guidelines.xhtml

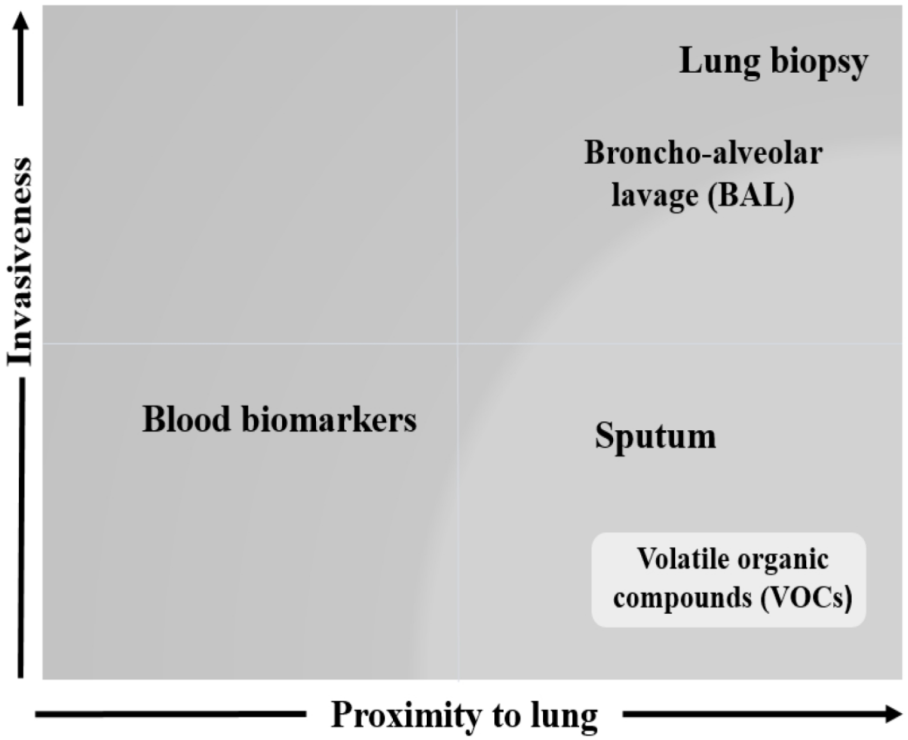

Figure (1): Relationship between lung proximity and degree of invasiveness of different lung matrices. The figure plots the level of invasiveness of various lung matrices in relation to their proximity to the lung. Given their pathological relevance, the degree of invasiveness of bronchoalveolar lavage (BAL) and lung biopsy makes them less favourable in diagnosing respiratory diseases.

144x115mm (300 x 300 DPI)

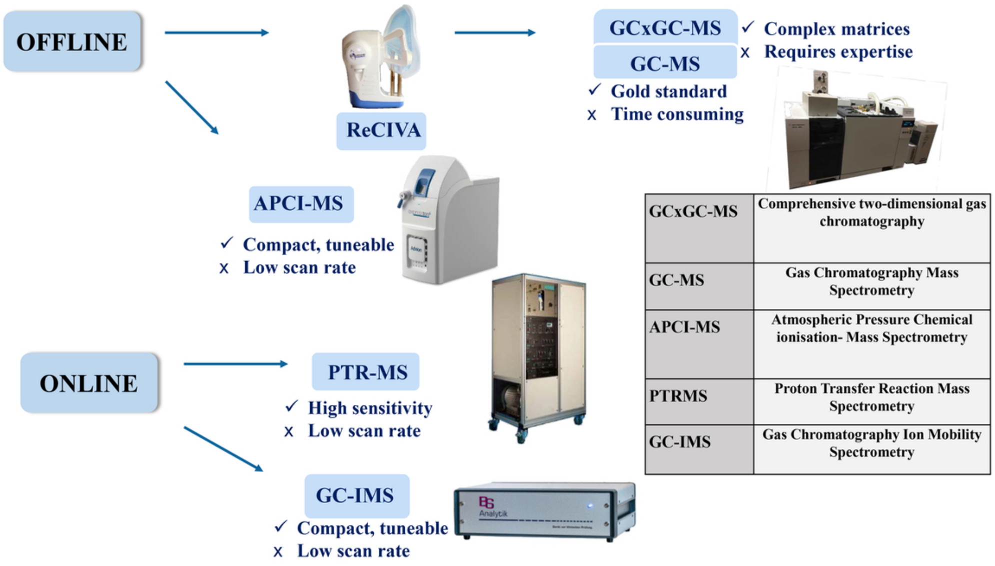

Figure (2): Multi-instrument use in breath sampling. Figure illustrates the various combinations of offline and online devices used in breath sampling and the relevant pros and cons. Offline and online technologies are used for the discovery and validation phases of the study respectively.

97x55mm (300 x 300 DPI)

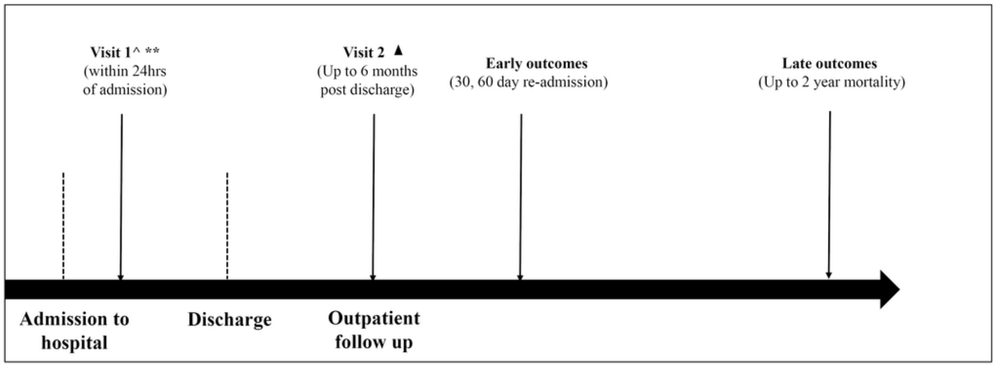

^ Following senior decision maker review
** Breath sampling (ReCIVA-GC-MS, ReCIVA- GCxGC-MS, GC-IMS, APCI-MS)
▲ Breath sampling (ReCIVA-GC-MS, ReCIVA- GCxGC-MS, PTR-MS, APCI-MS)
---- Following University Hospitals of Leicester streaming and clinical care pathways

Figure (3): Study flow chart. Figure outlines the patient journey from admission through to discharge and follow up. Participants with self-reported acute breathlessness presenting to University Hospitals of Leicester are recruited within 24 hours following a senior decision maker review. Breath sampling is carried out on the first visit, at recruitment, and the second visit, up to 6 months after discharge. Patients are admitted through the standard operational emergency medical streaming and care pathways at the University Hospitals of Leicester NHS Trust. Early outcomes (hospital readmission) are measured at 30 and 60 days and late outcomes (mortality) including respiratory and all-cause mortality are measured at 2 years. Assessments carried out at each time point are summarised in Table 1.

86x43mm (300 x 300 DPI)

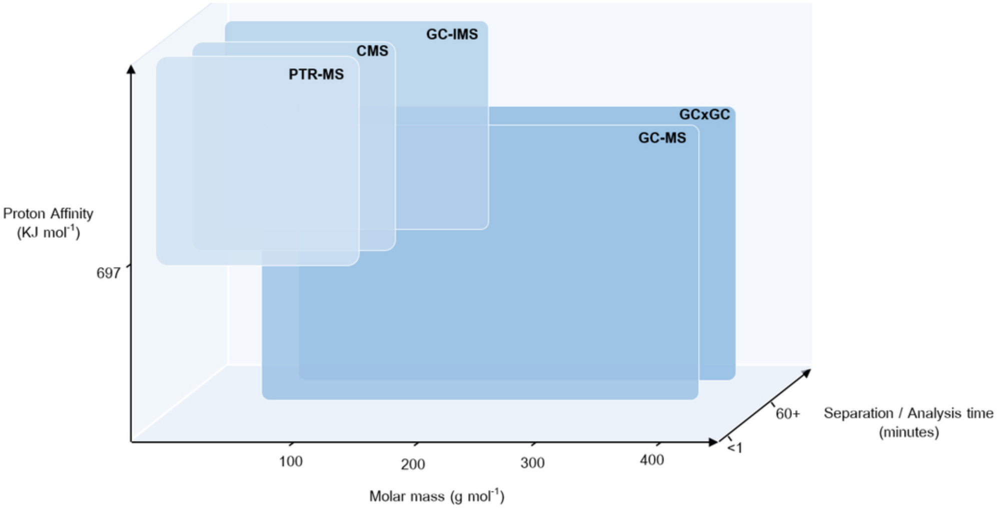

Figure (4): Multi-instrument use in breath sampling. Operational space of the analytical technologies used in EMBER for the analysis of volatile organic compounds (VOC) in exhaled breath, including proton transfer reaction-mass spectrometry (PTR-MS), atmospheric pressure chemical ionisation-mass spectrometry (CMS), gas chromatography-ion mobility spectrometry (GC-IMS), gas chromatography-mass spectrometry (GC-MS) and two-dimensional gas chromatography-mass spectrometry (GCxGC). Comparing the typical molar mass range detectable; selectivity in detection owing to the type of ionisation involved and the proton affinity of analytes; and the inclusion of a chromatographic separation affecting total time of analysis. The online technologies involving chemical ionisation (PTR-MS, CMS and GC-IMS) can be used in-clinic owing to short analysis times, but only detect lower molar mass molecules with a proton affinity higher than 697 KJ mol-1. Offline chromatographic techniques (GC-MS and GCxGC) detect a wider range of compounds independent of proton affinity; however, the techniques have longer analysis times and involve sample transportation and storage.

88x44mm (300 x 300 DPI)

BMJ Open

Assessment of Breath Volatile Organic Compounds in Acute Cardio-respiratory Breathlessness: A protocol describing a Prospective Real World Observational Study

Journal:	*BMJ Open*
Manuscript ID	bmjopen-2018-025486.R2
Article Type:	Protocol
Date Submitted by the Author:	23-Nov-2018
Complete List of Authors:	Ibrahim, Wadah; Leicester NIHR Biomedical Research Centre (Respiratory theme); College of Life Sciences, Department of Infection, Immunity and Inflammation, University of Leicester<br>Wilde, Michael; University of Leicester, Department of Chemistry<br>Cordell, Rebecca; University of Leicester, Department of Chemistry<br>Salman, Dahlia; Loughborough University, Department of Chemistry<br>Ruszkiewicz, Dorota; Loughborough University, Department of Chemistry<br>Bryant, Luke; University of Leicester, Department of Chemistry<br>Richardson, Matthew; Leicester NIHR Biomedical Research Centre (Respiratory theme); University of Leicester, Infection, Inflammation and Immunity<br>Free, Robert; Leicester NIHR Biomedical Research Centre (Respiratory theme); University of Leicester, Infection, Inflammation and Immunity<br>Zhao, Bo; Leicester NIHR Biomedical Research Centre (Respiratory theme); University of Leicester, Infection, Inflammation and Immunity<br>Yousuf, Ahmed; University of Leicester, Infection, Inflammation and Immunity; Leicester NIHR Biomedical Research Centre (Respiratory theme)<br>White, Christobelle; University of Leicester, Infection, Inflammation and Immunity; Leicester NIHR Biomedical Research Centre (Respiratory theme)<br>Russell, Richard; University of Leicester, Infection, Inflammation and Immunity; Leicester NIHR Biomedical Research Centre (Respiratory theme)<br>Jones, Sheila; University Hospitals of Leicester NHS Trust<br>Patel, Bharti; University Hospitals of Leicester NHS Trust<br>Awal, Asia; University Hospitals of Leicester NHS Trust<br>Phillips, Rachael; NIHR Leicester Clinical Research Facility<br>Fowkes, Graham; NIHR Leicester Clinical Research Facility<br>McNally, Teresa; Paediatric Clinical Investigation Centre<br>Foxon, Clare; Paediatric Clinical Investigation Centre<br>Bhatt, Hetan; Leicester NIHR Biomedical Research Centre (Respiratory theme)<br>Peltrini, Rosa; Leicester NIHR Biomedical Research Centre (Respiratory theme); University of Leicester, Department Infection, Inflammation and Immunity<br>Singapuri, Amisha; Leicester NIHR Biomedical Research Centre (Respiratory theme)<br>Hargadon, Beverley; Leicester NIHR Biomedical Research Centre

(Respiratory theme); University of Leicester, Infection, Inflammation and Immunity
Suzuki, T; Department of Cardiovascular Sciences, University of Leicester, , Cardiovascular Research Centre; Leicester NIHR Biomedical Research Centre (Cardiovascular theme)
Ng, Leong; Department of Cardiovascular Sciences, University of Leicester, Cardiovascular Research Centre; Leicester NIHR Biomedical Research Centre (Cardiovascular theme)
Gaillard, Erol; Leicester NIHR Biomedical Research Centre (Respiratory theme); Paediatric Clinical Investigation Centre
Beardsmore, Caroline; Leicester NIHR Biomedical Research Centre (Respiratory theme); Paediatric Clinical Investigation Centre
Ryanna, Kimuli; University Hospitals of Leicester NHS Trust
Pandya, Hitesh; Discovery Medicine, Respiratory Therapeutic Area, GlaxoSmithKline PLC
Coates, Tim; University of Leicester, Infection, Inflammation and Immunity; Department of Cardiovascular Sciences, University of Leicester
Monks, Paul; University of Leicester, Infection, Inflammation and Immunity; University of Leicester, Department of Chemistry
Greening, Neil; Leicester NIHR Biomedical Research Centre (Respiratory theme); University of Leicester, Infection, Inflammation and Immunity
Brightling, Christopher; Leicester NIHR Biomedical Research Centre (Respiratory theme); University of Leicester, Infection, Inflammation and Immunity
Thomas, Paul; Loughborough University, Department of Chemistry
Siddiqui, Salman; Leicester NIHR Biomedical Research Centre (Respiratory theme); University of Leicester, Infection, Inflammation and Immunity

**Primary Subject Heading**:	Respiratory medicine
Secondary Subject Heading:	Research methods, Respiratory medicine
Keywords:	Breathlessness, Observational study, Volatile Organic Compound, Breath analysis

SCHOLARONE™
Manuscripts

Assessment of Breath Volatile Organic Compounds in Acute Cardio-respiratory Breathlessness: A protocol describing a Prospective Real World Observational Study

Wadah Ibrahim[1,2], Michael Wilde[3], Rebecca Cordell[3], Dahlia Salman[4], Dorota Ruszkiewicz[4], Luke Bryant[3], Matthew Richardson[1,2], Robert C. Free[1,2], Bo Zhao[1,2], Ahmed Yousuf[1,2], Christobelle White[1,2], Richard J Russell[1,2], Sheila Jones[5], Bharti Patel[5], Asia Awal[5], Rachael Phillips[6], Graham Fowkes[6], Teresa McNally[7], Clare Foxon[7], Hetan Bhatt[1,2], Rosa Peltrini[1,2], Amisha Singapuri[1,2], Beverley Hargadon[1,2], Toru Suzuki[8,9], Leong L. Ng[8,9], Erol A Gaillard[2,7], Caroline Beardsmore[2,7], Kimuli Ryanna[5], Hitesh Pandya[11], Tim Coates[2,10], Paul S. Monks[2,3], Neil Greening[1,2], Chris E Brightling[1,2], Paul Thomas[4], Salman Siddiqui[1,2]

wadah.ibrahim@leicester.ac.uk; mjw77@leicester.ac.uk; rc145@leicester.ac.uk; d.salman@lboro.ac.uk; d.m.ruszkiewicz2@lboro.ac.uk; lb368@leicester.ac.uk; mr251@leicester.ac.uk; rcf8@leicester.ac.uk; bo.zhao@leicester.ac.uk; ajy7@leicester.ac.uk; cmjw3@leicester.ac.uk; rjr22@leicester.ac.uk; rjr22@leicester.ac.uk; sheila.jones@uhl-tr.nhs.uk; bharti.m.patel@uhl-tr.nhs.uk; asia.awal@uhl-tr.nhs.uk; rachael.phillips@uhl-tr.nhs.uk; graham.fowkes@uhl-tr.nhs.uk; tm23@leicester.ac.uk; clare.foxon@uhl-tr.nhs.uk; hetan.bhatt@uhl-tr.nhs.uk; rp417@leicester.ac.uk; amisha.singapuri@uhl-tr.nhs.uk; beverley.hargadon@uhl-tr.nhs.uk; ts263@le.ac.uk; lln1@le.ac.uk; eag15@leicester.ac.uk; csb@leicester.ac.uk; kimuli.ryanna@uhl-tr.nhs.uk; hitesh.x.pandya@gsk.com; tim.coates@leicester.ac.uk; paul.monks@leicester.ac.uk; neil.greening@leicester.ac.uk; ceb17@leicester.ac.uk; C.L.P.Thomas@lboro.ac.uk; ss338@leicester.ac.uk

Corresponding author: Professor Salman Siddiqui, ss338@leicester.ac.uk

NIHR Leicester Biomedical Research Centre (Respiratory Theme)

Glenfield Hospital,

Groby Road

Leicester

LE3 9QP

t: +44 116 256 3841

e: ss338@ leicester.ac.uk

Authors' affiliations:

[1]Leicester NIHR Biomedical Research Centre (Respiratory theme), Glenfield Hospital, Groby Road, Leicester LE3 9QP

[2] College of Life Sciences, Department of Infection, Immunity and Inflammation, University of Leicester, University Road, Leicester LE1 7RH

For peer review only - http://bmjopen.bmj.com/site/about/guidelines.xhtml

[3] Department of Chemistry, University of Leicester, University Road, Leicester, LE1 7RH

[4]Department of Chemistry, Loughborough University, Epinal Way, Loughborough, LE11 3TU

[5] University Hospitals of Leicester NHS Trust, Glenfield Hospital, Groby Road, Leicester LE3 9QP

[6] NIHR Leicester Clinical Research Facility, Research Space, Level 0, Balmoral Building, Leicester Royal Infirmary, Infirmary Square, Leicester, LE1 5WW

[7] Paediatric Clinical Investigation Centre, Leicester Respiratory Biomedical Research Centre, University of Leicester, Leicester Royal Infirmary, Leicester, LE2 7LX

[8] Department of Cardiovascular Sciences, University of Leicester, Cardiovascular Research Centre, Glenfield General Hospital, Leicester LE3 9QP.

[9] Leicester NIHR Biomedical Research Centre (Cardiovascular theme), Glenfield Hospital, Groby Road, Leicester LE3 9QP

[10]Department of Cardiovascular Sciences, University of Leicester, Level G, Jarvis building RMO, Infirmary Square, Leicester LE1 5WW

[11] Discovery Medicine, Respiratory Therapeutic Area, GlaxoSmithKline PLC, Gunnels Wood Road, Stevenage, Hertfordshire, SG1 2NY

For peer review only - http://bmjopen.bmj.com/site/about/guidelines.xhtml

Abstract

Introduction

Patients presenting with acute undifferentiated breathlessness are commonly encountered in admissions units across the United Kingdom. Existing blood biomarkers have clinical utility in distinguishing patients with single organ pathologies but have poor discriminatory power in multi factorial presentations. Evaluation of volatile organic compounds (VOC) in exhaled breath offers the potential to develop biomarkers of disease states that underpin acute cardio-respiratory breathlessness, owing to their proximity to the cardio-respiratory system. To date there has been no systematic evaluation of VOC in acute cardio-respiratory breathlessness. The proposed study will seek to use both offline and online VOC technologies to evaluate the predictive value of VOC in identifying common conditions that present with acute cardio-respiratory breathlessness.

Methods and analysis

A prospective real world observational study carried out across three acute admissions units within Leicestershire. Participants with self-reported acute breathlessness, with a confirmed primary diagnosis of either acute heart failure, community acquired pneumonia and acute exacerbation of asthma or COPD will be recruited within 24 hours of admission. Additionally, school age children admitted with severe asthma will be evaluated. All participants will undergo breath sampling on admission and upon recovery following discharge. A range of online technologies including: proton-transfer-reaction mass spectrometry (PTR-MS), gas chromatography ion mobility spectrometry (GC-IMS), atmospheric pressure chemical ionisation- mass spectrometry (APCI-MS) and offline technologies including gas chromatography mass spectroscopy (GC-MS) and comprehensive two-dimensional gas chromatography-mass spectrometry (GCxGC-MS) will be utilised for VOC discovery and replication. For offline technologies a standardised CE marked breath sampling device (ReCIVA®) will be used. All recruited participants will be characterised using existing blood biomarkers including C - reactive protein (CRP), brain derived natriuretic peptide (BNP), Troponin-I

For peer review only - http://bmjopen.bmj.com/site/about/guidelines.xhtml

and blood eosinophil levels and further evaluated using a range of standardised questionnaires, lung function testing, sputum cell counts and other diagnostic tests pertinent to acute disease.

Ethics and dissemination

The National Research Ethics Service Committee East Midlands has approved the study protocol (REC number: 16/LO/1747). IRAS 198921. Findings will be presented at academic conferences and published in peer-reviewed scientific journals. Dissemination will be facilitated via a partnership with the East Midlands Academic Health Sciences Network and via interaction with all UK funded MRC/EPSRC molecular pathology nodes.

Key words: Breathlessness; Breath analysis; Volatile Organic Compound, Observational study

For peer review only - http://bmjopen.bmj.com/site/about/guidelines.xhtml

Strengths and Limitations of this Study

- A pragmatic real world, prospective, observational study across three admission units that focuses on the systematic discovery and replication of VOC in acutely breathless patients using both online and offline technologies
- The proposed study is the largest of its kind in acute disease to characterise VOC with a range of additional assessments that will build a comprehensive phenotype of acute cardio-respiratory exacerbations
- The proposed study will build an infrastructure for research and subsequent evaluation of VOC in interventional trials within acute cardio-respiratory exacerbations
- Prior acute treatment exposure will need to be accounted for when evaluating potential discriminative biomarkers
- VOC technologies are not currently suited for deployment in patients that are of high clinical acuity

1. Introduction:

Breathlessness is a common symptom of cardio-respiratory illnesses that has a significant direct impact on patients' wellbeing as well as a substantial economic burden on healthcare systems [1]. Although its etiologies can be variable, exacerbations of common complex chronic cardio-respiratory conditions account for approximately 70% of acute presentations with breathlessness, namely exacerbations of asthma and COPD, acute heart failure and community acquired pneumonia [2]. Moreover, moderate and severe breathlessness is significantly associated with all-cause, cardiovascular and COPD mortality[3]. As a consequence symptomatic breathlessness warrants rapid evaluation and targeted diagnostics at presentation.

Diagnostic evaluation of acute breathlessness is heavily reliant on blood based biomarkers e.g. CRP, BNP, Troponin and on occasions blood eosinophil levels. These biomarkers have clinical utility primarily in patients with single pathologies, but have poor discriminatory power in patients with

For peer review only

multifactorial presentations of acute breathlessness[4]. There is therefore an unmet need for the development of sensitive and specific biomarkers that differentiate acute breathlessness from its recovery and the common cardio-respiratory conditions that present with acute breathlessness.

CRP plays an important role in diagnosing breathlessness caused by an underlying bacterial pneumonia[5], as well as predicting mortality in patients with COPD [6]. BNP is routinely utilised in acute settings to support the diagnosis of acute heart failure [7].The European Society of Cardiology (ESC) recommends BNP threshold values of <100 pg/mL to rule out acute congestive cardiac failure and values > 500 pg/ml as diagnostic of acute exacerbations of heart failure [8].

The role of peripheral blood eosinophil count in airway inflammation was poorly understood up until the second half of the 19th century when Paul Ehrlich, a German physician and Nobel prize winner, introduced eosin in his technique for white cell differentiation in 1879[9] . Considerable advances in the field of airway inflammation and the role of eosinophils have taken place since [10-12]. More recently Bafadhel *et al* suggested that peripheral blood eosinophil count can be used to direct corticosteroid therapy during COPD exacerbations in single centre study [13].

Currently, blood biomarkers together with clinical, physiological and imaging parameters are used in diagnosing the cause of acute breathlessness. Blood biomarkers may be less specific as they originate far from the target organs of interest (the heart and the lungs in cardio-respiratory disease). Sputum, although potentially a more definitive lung specific matrix, is comparatively difficult to obtain particularly in acutely unwell patients, limiting its use in acute disease and highlighting the need for better biomarkers. Ideally these biomarkers would have the following characteristics, (i) they would originate from the target organ of interest, (ii) they would significantly add value to conventional risk scoring and diagnostic algorithms in acute breathlessness, (iii) they would be minimally invasive and suitable for rapid point of care diagnosis in emergency rooms and acute admissions units (iv) they would have diagnostic value in patients with multifactorial acute breathlessness.

For peer review only - http://bmjopen.bmj.com/site/about/guidelines.xhtml

Exhaled breath contains thousands of volatile organic compounds (VOC) that reflect biological processes occurring in the host both locally in the airways and systematically offering the potential to develop more effective biomarkers in acutely breathless patients (**Figure 1**).

The proposed program of research will use a combination of offline and online technologies to identify and evaluate the diagnostic and prognostic value of VOC in patients with acute cardio-respiratory related breathlessness (**Figure 2**).

Exhaled breath analysis of volatile chemicals offers the potential to develop more effective biomarkers in acutely breathless patients. The use of breath analysis for disease diagnostics dates back to ancient Greeks where physicians used exhaled breath to diagnose different diseases. Breath odours allow correct associations to certain diseases. For example, the sweet smell of diabetic ketoacidosis, the fishy smell of breath associated to liver illness, the urine-like odour of kidney disease and the smell of the breath of patients with lung abscesses, caused by the proliferation of anaerobic bacteria [14-17]. More recently, exhaled breath analysis has demonstrated early proof of concept in the diagnosis of acute heart failure, and ventilator associated pneumonia[18] . The validity of breath analysis has also been demonstrated in breathless children[19]. This population is likely to prefer breath-based tests, as these are minimally invasive. Importantly, a variety of point of care sensors are now available to evaluate potential exhaled breath biomarkers in emergency care settings.

A study by Van Berkel et al demonstrated the ability to distinguish COPD subjects from controls solely based on the presence of VOCs in breath, suggesting that analysis of VOC might be highly relevant for diagnosis of COPD [20]. This established the basis of further studies of VOC in COPD [21-25].recommending larger studies for validation.

Several other studies found that VOC profiling in diagnosing asthma is potentially feasible [26-32].

This however has been done in relatively small numbers in stable disease.

Despite the novelty of non-invasive sampling technology and the growing interest in exhaled breath analysis there remains a disappointing level of comparability across studies due to the lack of

For peer review only - http://bmjopen.bmj.com/site/about/guidelines.xhtml

standardisation and appropriate data analysis methods. A recent systemic review by Anders Christiansen *et al* compared eleven publications reporting very heterogeneous designs, methods, patient group sizes, data analytics and, consequently, quite varying results [33].

To our knowledge, no other large studies exploring the use of breath biomarkers in profiling acute breathlessness have been completed. Several studies have explored the use of electronic nose (eNose) in stable disease with good discriminatory power in COPD [34], Pneumonia [35] and heart failure[36] with relatively small sample size. While eNose has now been widely used in detecting various VOC patterns, GC-MS, a largely validated methodology, remains the gold standard technique for detecting VOCs in exhaled breath. The focus of the current research study will be to evaluate acutely breathless cardio-respiratory patients using a combination of 'discovery' and near-patient care breath sampling technologies.

Medical Research Council (MRC) and Engineering and Physical Sciences Research Council (EPSRC) have commissioned a series of molecular pathology nodes aimed at developing molecular signatures relevant to disease diagnosis and progression. This was triggered by the clear need for alliance between academic institutions, industry and NHS partners to enhance the benefits of stratified medicine for patients[37, 38].

University of Leicester and Loughborough University were awarded a joint molecular pathology node East Midlands Breathomics Pathology Node (EMBER) which this study forms a key part of.

2. Methods and Analysis

2.1. Study design

A prospective real world observational study across three acute admissions units within Leicestershire (two adult admissions units and one children's assessment unit). The acute units routinely assess and treat cardio-respiratory admissions due to breathlessness in adults and children.

For peer review only

Participants with self-reported acute breathlessness, either requiring admission or a change in baseline treatment, will be screened for the study. Informed consent will be obtained in all participants following a clinical review by a senior decision maker within 24 hours of acute admission (**Figure 3**).

2.2. Objectives

2.2.1. Primary objective

- To evaluate the sensitivity, specificity, positive and negative predictive value of exhaled breath VOC biomarkers to differentiate acute breathlessness in cardio-respiratory patients.

2.2.2. Secondary objectives:

- To replicate selected breath VOC biomarkers identified in acute breathlessness.
- To discover and replicate breath VOC biomarkers that differentiate the common cardio-respiratory conditions that cause acute breathlessness, specifically (i) acute heart failure, (ii) community acquired pneumonia, (iii) adult exacerbations of asthma and COPD and age-matched adults that do not have cardio-respiratory disease or breathlessness.
- To quantify the level of clinical uncertainty in the primary diagnosis using a 100 mm visual analogue scale and independent clinical adjudication of case notes blinded to the following blood biomarkers (i) CRP (ii) BNP (iii) Troponin-I (iv) blood eosinophils, but not clinical history and acute presentation nor chest x-ray imaging. Potential discriminatory breath VOC biomarkers will be adjusted for clinical uncertainly in statistical models.
- To identify and replicate exhaled breath VOC biomarkers in school age children treated in hospital for severe asthma attacks and compare these to age-matched healthy controls.

2.2.3. Exploratory end points (where applicable):

- To evaluate the dynamic profile of selected breath VOC between the acute state and the recovery state post exacerbation.

For peer review only - http://bmjopen.bmj.com/site/about/guidelines.xhtml

- To evaluate the relationship between exhaled VOC biomarkers and clinical outcomes including (i) hospital readmission at 30 and 60 days post event (ii) all-cause mortality over a 2 year period post admission.
- To evaluate the relationship between breath VOC biomarkers and functional measures e.g. physical performance and activity
- To explore potential breath VOC biomarkers of multifactorial acute breathlessness
- To evaluate the relationship between diet, lifestyle and environment upon breath VOC biomarkers

2.3. Sample size estimation

Preliminary data was utilised to conduct sample size estimates from a cohort of acutely breathless patients admitted to acute admissions units over a 6 month period (February 2017 to August 2017). Hundred and twelve adult participants (asthma 46, community acquired pneumonia 26, COPD 22) and eighteen healthy controls were utilised for the analysis.

A panel of ten pre-specified aldehydes, based on literature search [31, 39, 40], were extracted from breath using gas chromatography-mass spectrometry (GC-MS). The aldehydes were normalised to a common internal standard and were not background-subtracted.

A closed formula from Hsieh *et al*[41], relating sample size to observable effect size, was used to calculate sample size from logistic regression models of the ten aldehydes with acute breathlessness as the outcome measure. The sample size estimates are also relevant to acute class comparisons vs. the sum of other acute classes.

Based upon the sample size estimates we would have an 80% power, with a type 1 error rate of 5%, to detect an odds ratio of association of 1.2 between two disease classes with 55 patients per class. Given the fact that study seeks to discover and replicate breath VOC amongst five adult disease classes (community acquired pneumonia, heart failure, COPD, asthma and healthy aged matched subjects) we would require 110 adult patients per class – 550 patients across the program to achieve these aims.

For peer review only - http://bmjopen.bmj.com/site/about/guidelines.xhtml

The closed formulae by Tihaki *et al*,[42] were also utilised to understand the discriminatory power that the samples sizes above would provide with respect to biomarker sensitivity and specificity; The following assumptions were made:

- That a sensitivity of 80 % with a precision of 5% would provide a useful biomarker capable of ‘ruling out’ an acute class. The same target was applied to specificity.
- We assume a prevalence of acute breathlessness of 80% as the recruitment campaign uses acute breathlessness as the initial stratification tool for recruitment and 1:5 patients recruited will be non-breathless healthy controls
- We aim to balance group sizes across classes equally

For a type 1 error rate of 0.05 and a 95% confidence interval

$N_{sensitivity}$= 307

$N_{specificity}$= 1,230

For a type 1 error rate of 0.05 and a 90% confidence interval

$N_{sensitivity}$= 218

$N_{specificity}$ = 871

For a type 1 error rate of 0.05 and an 85% confidence interval

$N_{sensitivity}$ = 166

$N_{specificity}$ = 664

For a type 1 error rate of 0.05 and an 80% confidence interval

$N_{sensitivity}$ = 131

$N_{specificity}$ = 524

Therefore, we are powered to identify sensitive biomarkers (≥ 80%) of acute breathlessness with a maximum marginal error in the estimate for sensitivity not exceeding 5% with 95% confidence. Similarly, we are powered to identify specific biomarkers (≥ 80%) of acute breathlessness with a maximum marginal error in the estimate for specificity not exceeding 5% with 80% confidence.

For peer review only

For peer review only - http://bmjopen.bmj.com/site/about/guidelines.xhtml

For the primary analysis the outcome will be treated as a nominal variable with levels (i) acute heart failure (ii) community acquired pneumonia (iii) adult exacerbations of asthma and COPD and (iv) acute exacerbations in school age children treated in hospital for severe asthma attacks.

The relationship between the primary outcome and the exhaled breath VOC biomarkers will be modelled using multinomial logistic regression. In addition to metabolomics markers the following independent variables will be included in the model: clinical uncertainty score on a 100 mm VAS scale, age, and a validated co morbidity score (the Charlson comorbidity score)[43, 44].

Receiver operator analyses will be utilised to generate ROC curves for individual and multiple panels VOC predictors in the primary analysis.

To understand the dynamic profile of breath biomarkers during (i) the acute state and (ii) in the chronic state up to 6 months post exacerbation, a repeated measures model with a random intercept and random effect for time will be fitted, the random effects will be fitted for each patient. For the repeated measures mixed model an unstructured covariance will be assumed. To evaluate the relationship between breath biomarkers and hospital readmission at 30 and 60 days Cox proportional hazards and frailty models will be utilised [45]. Analysis of Multivariate Survival Data, [CITE] competing risk models and joint models will be fitted [46]. Relationship between death and breath biomarkers will be evaluated using a logistic regression model. Changes in outcome measures will be measured appropriately for each variable (e.g. paired t-test, Mann-Whitney, repeated measures analysis).Tables of descriptive statistics will be compiled for all key variables

All analysis will be performed using R 3.5.0 https://www.r-project.org/.

2.4. Discovery and Replication studies

Specific indicator conditions have been selected for targeted recruitment according to their high prevalence and unmet need, their high morbidity and mortality and the need to develop better diagnostic and prognostic algorithms in acute care pathways.

For peer review only - http://bmjopen.bmj.com/site/about/guidelines.xhtml

The indicator diagnoses of interest are (i) exacerbations of adult asthma and COPD, (ii) community acquired pneumonia (iii) acute heart Failure and (iv) exacerbation in school age children treated in hospital for severe asthma attacks.

Patient level clinico-pathological and outcome data (spanning the entire acute pathway) will be collected in parallel to breath sampling. In addition, breath samples will be acquired in the stable state post exacerbation (**Figure 3**).

Age matched healthy volunteers will be recruited where possible at separate visits. For the purposes of this study, healthy volunteers will be defined as participants who have no prior history of asthma, COPD, heart failure and have not been admitted to hospital with community acquired pneumonia within 6 weeks of the baseline study visit. For acute admission the study team will approach the spouse, parent or sibling of the index case and seek informed consent for study assessments. All healthy subjects will undergo two assessments separated by a duration of 8-16 weeks to match the acute and recovery time points elapsed in their index case/partner/spouse/sibling/child. Additional healthy volunteers will be identified from local recruitment databases and via advertising

2.4.1. Discovery Phase (Project months 1-24):

The aim of the discovery phase is to discover putative discriminatory breath VOC, using both offline and online technologies.

Pre-planned recruitment of acutely breathless patients will be enriched into the following disease strata following senior clinical decision maker assessment and within 24 hours of acute admission. Acute adult heart failure (n=55), adult community acquired pneumonia (n=55), adult exacerbations of asthma (n=55) and COPD (n=55), acute severe asthma attacks in school age children (n=50). Additional age matched healthy volunteers (n=55 adults and 50 children) will be identified as a non-disease reference group **(Table 1).**

For peer review only - http://bmjopen.bmj.com/site/about/guidelines.xhtml

2.4.2. Replication Phase (years 3-4)

The aim of the replication phase is to replicate putative discriminatory breath VOC /VOC signatures identified in the discovery phase.

Similar to the discovery phase, recruitment of acutely breathless patients will be enriched into the following disease strata following senior clinical decision maker assessment and within 24 hours of acute admission.

Acute adult heart failure (n=55), adult community acquired pneumonia (n=55), adult exacerbations of asthma (n=55) and COPD (n=55), acute severe asthma attacks in school age children (n=25) **(Table 1)**.

Additional age matched healthy volunteers (n=55 adults and 25 children) will be identified as a non-disease reference group.

Total combined sample size of the discovery and replication phases = 700 participants

**Disease Category**	**Discovery**	**Replication**
Acute Adult Asthma	**55**	**55**
Acute COPD	**55**	**55**
Acute Heart Failure	**55**	**55**
Community Acquired Pneumonia	**55**	**55**
Adult healthy volunteers	**55**	**55**
Acute paediatrics Asthma	**50**	**25**
Paediatrics healthy volunteers	**50**	**25**
**Total sample**	**375**	**325**

Table (1): Table summarising recruitment targets for both adult and paediatric groups.

2.5. Schedule of assessments

A schedule of acute assessments is outlined below and aligns to the movement of acute patients through the clinical care pathway and the overall aim of developing a complete phenotypic picture of acutely breathless patients.

2.5.1. Defining acute breathlessness

At presentation (within 24 hours of admission) to one of three acute admissions units potentially eligible patients will be identified following confirmation of acute breathlessness, identified as (i) patient defined acute breathlessness and/or (ii) 1 unit increase above patient reported baseline in the extended medical research council (eMRC) dyspnoea score [47, 48] and at least one of the indicator

For peer review only - http://bmjopen.bmj.com/site/about/guidelines.xhtml

diagnoses identified as the primary clinical diagnosis by a senior clinical decision maker. eMRC will be completed by all patients and healthy volunteers at each research visit.

2.5.2 Informed consent

Patients meeting the pre-specified definition of acute breathlessness will be approached for informed consent in to the breath VOC biomarker study. Only patients that are eligible to give full written informed consent will be recruited.

2.5.3 Collection of blood based pathology markers

Collection of the blood biomarkers CRP, BNP, Troponin-I and blood eosinophil count will be performed both acutely and following recovery, when not taken as part of clinical care pathway. These are currently used in profiling acutely breathless patients in clinical practice (**Table2**).

Test	ANALYSER/METHOD	LOWER LIMIT OF DETECTION	UPPER LIMIT OF DETECTION
C-Reactive protein (CRP)	Siemens Advia Chemistry XPT, PEG enhanced immunoturbidimetric. Siemens Advia 1800, PEG enhanced immunoturbidimetric	5 mg/L	Diluted to result
B-type natriuretic peptide (BNP)	Siemens Advia Centaur XPT, two-site sandwich immunoassay using direct chemiluminescent technology	2.0 pg/mL	1445 pg/mL
Troponin-I	Abbott Architect i2000SR, three-site sandwich immunoassay using direct chemiluminescent technology (CMIA).	5.0 ng/L	50,000 ng/L

Table (2): Type of analyser and methodology used for blood biomarker calculation. The table outlines analyser make, methodology, upper and lower limits of detection as per the University Hospitals of Leicester NHS Foundation trust laboratory guidelines.

2.5.4 Breath VOC sampling

Offline breath sampling using GC-MS and comprehensive two-dimensional gas chromatography-mass spectrometry coupled with a standardised and CE marked breath sampler-ReCIVA®[49], will be performed. Gas chromatography is considered a gold standard technique in detecting volatile organic compounds and as such its sampling will be prioritised. Additionally the following online technologies, proton transfer mass spectroscopy (PTR-MS), gas chromatography - ion mobility

For peer review only

For peer review only - http://bmjopen.bmj.com/site/about/guidelines.xhtml

spectroscopy (GC-IMS) and atmospheric pressure chemical ionisation- mass spectrometry (APCI-MS) will be evaluated according to the sampling strategy outlined in section 3, **Figure 3 and Table 3**.

	COPD		Asthma		Pneumonia		Heart Failure		Healthy		Paediatrics	
Time point	1	2	1	2	1	2	1	2	1	2	1	2
Written informed consent	x		x		x		x		x		x	
**Volatile organic compound (VOC) sampling**												
ReCIVA- gas chromatography and mass spectrometry (GC-MS)	x	x	x	x	x	x	x	x	x	x	x	x
ReCIVA- comprehensive two-dimensional gas chromatography (GCxGC-MS)	x	x	x	x	x	x	x	x	x	x	x	x
Atmospheric pressure chemical ionisation- mass spectrometry (APCI-MS)	x	x	x	x	x	x	x	x	x	x	x	x
Proton transfer reaction mass spectrometry (PTRMS)		x		x		x		x	x	x	x	x
Gas chromatography - ion mobility spectrometry (GC-IMS)	x		x		x		x				x	x
**Pathology blood tests**												
Full blood count (including differential cell count)	x	x	x	x	x	x	x	x	x	x	x	x
Brain natriuretic peptide (BNP) [pg/mL]	x	x	x		x		x	x	x			
Troponin-I]ng/L]	x		x		x		x		x			
C-Reactive protein (CRP) [mg/L]	x	x	x	x	x	x	x	x	x	x	x	x
**Lung function tests**												
Hand held forced oscillation technique (FOT)	x	x	x	x	x	x	x	x	x	x		
Fractional exhaled nitric oxide (FeNO) - Flow rate 50 [ml/s]			x	x							x	x
Spontaneous sputum sample	x	x	x	x	x	x	x	x	x	x		
Bio-banking (urine, serum, plasma. sputum supernatants & plugs)	x	x	x	x	x	x	x	x	x	x	x	x
Transthoracic echocardiography	x						x					

Table (3): Summary of baseline and follow up assessments. The table summarises key assessments carried out at different time points during the study. The participants may undertake any combination of the investigations listed at any of these time points.

For peer review only - http://bmjopen.bmj.com/site/about/guidelines.xhtml

2.5.5. Collection of additional samples for future biomarker campaigns

Collection of additional biomarkers for future biomarker discovery campaigns including (i) a urine sample, (ii) blood samples, up to 85mls for DNA, RNA, plasma and serum and peripheral blood cell flow cytometry in selected subjects and (iii) spontaneous or induced sputum samples (plugs and supernatants) will be carried out (**Table 3**).

All samples will be collected at time point 1 and at 2 (**Figure 3**). The additional samples will be used for future omics analyses, these may include detailed analysis of the metagenome in sputum and proteomics applied to urine and serum samples.

2.5.6. Physiological characterisation

Physiological measures of lung function will be performed in acutely ill participants and at recovery including, (i) Hand held forced oscillation technique (FOT): an easily accessible measure of lung function. Patients favour this to spirometry as it is effort independent, unlike spirometry and requires less than a minute of quiet tidal breathing to obtain triplicate high quality measurements [50], This will be completed using Tremoflo®, Thorasys Thoracic Medical Systems Inc. (ii) Fractional exhaled nitric oxide (FeNO): A measure of airway inflammation in asthmatic patients[51, 52]. This instrument used for this will be NIOX VERO®, registered trademark of Circassia AB (PP-VERO-UK-0022-v1.0) (iii) Echocardiography: Two dimensional transthoracic echocardiography will be performed in heart failure and COPD patients using an iE 33 system with S5-1 transducer (frequency transmitted 1.7 MHz, received 3.4 MHz; Philips Medical Systems, Best, The Netherlands). Standard techniques as per American Society of Echocardiography guidelines (ASE)[53] will be used to acquire 2D, colour and Doppler images in conventional parasternal long-axis, short-axis and apical 4-, 2- and 3-chamber views. Left ventricular ejection fraction (LVEF)

For peer review only - http://bmjopen.bmj.com/site/about/guidelines.xhtml

will be calculated using the biplane method of discs formula (Simpson's rule) to derive left ventricular volume indices.

All participants are encouraged to report any testing related discomfort or concerns to the research team to terminate the sampling process.

2.5.7 Recovery follow up

- Patient recovery will be defined as:
  - (i) Patient reported recovery from the acute exacerbation spell and back to their baseline extended MRC score or clinician defined recovery from the acute exacerbation spell

  and

  - (ii) At least 6 weeks post exacerbation event (up to 6 months).

Patients that re admit to hospital between visits 1 and 2, can have additional visit 1 assessments. Visit 2 will be taken as recovery following the subsequent admission. If a patient is admitted to hospital after visit 2 then they will be eligible to be recruited as a new study participant.

The schedule of assessments at the recovery visit is outlined **(Table 3).**

2.6. Clinical Adjudication:

In an effort to reduce data variability and minimise bias, an independent panel consisting of two senior acute clinicians (SS & NG) will review all pertinent clinical and diagnostic source documentation, whilst blinded to admission blood biomarkers and clinical diagnosis.

All acutely breathless adult patients' notes will be sequentially divided for adjudication. The panel will independently determine the primary diagnosis of highest probability from a list of the four potential acute indicator diagnoses in section 2.4 and mark their level of clinical certainty on a 100 mm visual analogue scale (VAS scale). The panel members will be able to review imaging, electrocardiograms

For peer review only

For peer review only - http://bmjopen.bmj.com/site/about/guidelines.xhtml

(ECGs), and other relevant information but not admission blood based pathology tests. In a subset of patients adjudication will be validated by separate panel member to ensure between observer agreement using Bland-Altman analysis and inter-rater agreement of the primary diagnosis using Kohen's kappa via repeated evaluation of a subset of cases (see statistical methods section 2.3).

2.7. Clinical Informatics:

Clinical data collection will be undertaken using a securely-hosted bespoke database system (ADD) developed within the NIHR Leicester Biomedical Research Centre – Respiratory (BRC). The system links acute admission episodes to hospital pathology records; historical respiratory physiology tests; and demographic information. The system provides functionality to validate data entry; manually verify records and highlight incomplete records. A custom VOC 'module' has been be created to support data collection within the study visits (1 and 2), and standardise diagnoses and medications through the use of clinical ontologies as well as linking hospital records/tests to patient visits.

Non-ADD based clinical data (e.g. hospital admissions, re-admissions and mortality) will be extracted from the hospital data warehouse using identifiable patient identifiers, and subsequently pseudonymised prior to integration.

An informatics pipeline will be created to facilitate the transfer of chemo-metric data from remote computers to the data repository. This will include tools to (i) enforce the correct labelling of data sets (e.g. study number, visit, type/source of sample) prior to automated validated transfer to the repository; (ii) record information about the sample process; (iii) search and extract data sets from the repository for subsequent analysis. Prior to analysis, clinical and chemo-metric data will be integrated using the study number and any potentially identifiable information will be removed.

For peer review only - http://bmjopen.bmj.com/site/about/guidelines.xhtml

3. Breath profiling

The technologies utilised in the VOC study during discovery and replication phases are:

Offline technologies

- ReCIVA+ GC-MS
- ReCIVA + GC x GC-MS
-

Online technologies

- GC-IMS
- PTR-MS
- APCI-MS

Offline technologies will underpin the discovery analyses owing to their ability to identify chemical identity and their recognition as the analytical gold standard in exhaled breath VOC analysis [54]. In contrast online technologies will be utilised for VOC biomarker replication and at the recovery visits owing to their portability and potential for future point of care testing. **(Figure 4)**. A brief description of the core VOC platforms is provided below

A CE marked breath sampling device (ReCIVA®) developed by Owlstone Medical, will be used to sample breath onto two adsorbent Tenax tubes. Participants will be asked to breathe through the ReCIVA face mask for a maximum of 300 seconds, aiming for collection of ≥ 80% of the target sample volume of 1 litre, after which the Tenax tubes will be transferred to the laboratory for analysis. This effectively allows de-coupling of the breath sampling from the breath sensor and analysis platforms in selected patients that are not able to mobilise to a real time breath sampling device. The Owlstone ReCIVA sampler will be utilised in breath collection for offline technologies namely GC-MS and GCxGC-MS. The ReCIVA sampler is capable of entraining oxygen and is therefore suitable for patients with mild respiratory failure requiring low flow rates of oxygen to maintain target oxygen saturations [49].

For peer review only

3.1. Gas chromatography and mass spectroscopy (GC-MS): is a commonly applied methodology used to accurately measure trace gases in complex mixtures such as exhaled air [54]. Pre-concentrating breath volatiles by various means and subsequent analysis constitute a reliable and sensitive method for VOC analysis [55]. Despite its high sensitivity, it is however, a time consuming technique and carries a risk of contamination at the pre-concentration step. It is also not suitable for online and multiple measurements limiting its use as a point-of-care testing technology for VOC [56].

The instrument used will be an Agilent 7890A gas chromatogram with a 5977a quadrupole mass spectrometer (Agilent Technologies Ltd, Stockport, UK), interfaced with a Markes Unity 2 thermal desorptionunit (Markes International Ltd, Llantrisant, UK).

3.2. Comprehensive two-dimensional gas chromatography-mass spectrometry (GCxGC-MS): an advanced analytical technique for the analysis of complex organic matrices; its main advantage is the unparalleled separation power it affords over conventional one-dimensional chromatographic techniques [57]. Previous research, albeit sparse, has demonstrated the potential of GCxGC-MS for breath analysis with the number of VOC detected exceeding those detected by conventional GC-MS [58, 59]. GCxGC-MS of breath metabolites has been used for the identification of biomarkers related to glucose metabolism [60, 61], tuberculosis [62] and radiation response [63]. This has generated interest within the breath research community, however, such studies were conducted on a small scale (<50 patients) and involved the use of expensive detectors and modulators. Method development and analysis of the data-rich GCxGC chromatograms, however, can be time-consuming and require specialist knowledge.

The instrument used will be an Agilent 7890A gas chromatogram, fitted with a G3486A CFT flow modulator and a three-way splitter plate coupled to a flame ionisation detector and a HES 5977B quadrupole mass spectrometer (Agilent Technologies Ltd, Stockport, UK), interfaced with a Markes TD-100xr thermal desorption autosampler (Markes International Ltd, Llantrisant, UK).

3.3. Proton-transfer-reaction mass spectrometry (PTRMS): a real time technique, capable of simultaneously measuring the evolution of multiple gas metabolites from a single breath. It has been

For peer review only - http://bmjopen.bmj.com/site/about/guidelines.xhtml

used for the identification of potential useful VOC biomarkers for diagnosis of a variety of diseases: including various cancers[64-66], liver disease[67, 68] and respiratory disease[69]. It has several advantages in clinical settings, such as the speed of sampling, the instant result achieved and the lack of need for sample storage or shipping. However, owing to the lack of pre-concentration or chromatographic separation, sensitivity and definitive compound identification can be somewhat limited when compared to GC-MS.

Two breath sampling devices will be used. The first device is a Loccioni SOFIA GSI-S; the subject is required to exhale a single breath, five times (three if providing five samples proves too difficult) into a sterile mouthpiece connected to an electrostatic bacterial/viral filter whilst wearing a nose clip (all CE marked). Flow from the mouthpiece passes into a gas sampling interface capnograph (Loccioni GSI-S – CE marked) and real-time user feedback of flow is provided on screen, allowing the regulation of the breath sampling rate. The gas sampling interface acts to simultaneously trigger the acquisition of the PTR-ToF-MS data and the exhaled breath travels through the capnograph down a heated sample line into the ion source of the PTR-ToF-MS

The second breath sampling device is a ReCIVA breath sampler (Owlstone) with one of the adsorbent Tenax tubes replaced with an outlet tube adapted for online sampling. The exhaled breath is transferred to the PTR-ToF-MS via a heated transfer line connected to the outlet tube, continuously drawn at a constant flow rate by the PTR-ToF-MS. The online adaptation of the consumable adsorbent tube does not affect the CE mark of the ReCIVA sampling device.

Once the breath sample reaches the PTR-ToF-MS, via either breath sampler, the breath mixes with protonated water (H_3O^+) inducing proton transfer to the target volatile organic compounds (VOCs) present, resulting in their ionisation. Sample ions are then guided into the time of flight mass spectrometer and mass spectra, showing the abundance and mass of the VOCs present, are collected throughout the exhalation. Following sampling, mouthpieces, filters and nose clips are disposed of and all patient contacted surfaces wiped down with antiseptic cleaning wipes in preparation for the next patient.

For peer review only - http://bmjopen.bmj.com/site/about/guidelines.xhtml

The instrument used will be a Kore Series II high performance proton transfer reaction-time of flight-mass spectrometer (Kore Technology Ltd, Cambridge, UK).

3.4. Gas chromatography- ion mobility spectrometry (GC-IMS) (B&S Analytiks): Allows the detection of volatile organic compounds down to ultra-trace level (ng/L - to pg/L - range). For years, IMS has been used to discover potential discriminatory breath VOC in lung cancer [70, 71], COPD[72, 73] and asthma[73]. Sampling takes place using a Spiroscout spirometer. The patients exhale through a disposable mouth piece connected to a Teflon tube. A piezoelectric pressure sensor is used to monitor the breathing profile, this opens the sampling valve at the appropriate point in the breath profile to collect end-tidal breath in a sample loop of 10 mL volume. After filling this loop, the collected sample air is then transferred to a multicapillary column for a chromatographic separation, which is achieved in 12 min. The separated molecules are then transferred into the IMS, ionised and then separated according to their mobility in a weak electric field.

The technology's multiple advantages of ultra-sensitivity, portability, online sampling and short analysis time (typical analysis time of 10 minutes) with real time detection, brings a promise to provide immediate and potentially reliable results for point of care breath diagnostics. Another concept with IMS devices is that once the required breath signatures have been discovered using GC-MS, IMS offers the potential to be 'tuned' for selective detection of VOC.

The instrument used will be a BioScout a multi-capillary column gas chromatogram-ion mobility spectrometer, with a ^{63}Ni ion source, interfaced with a SpiroScout breath sampler (BS Analytik, Dortmund, Germany).

3.5. Atmospheric pressure chemical ionisation- mass spectrometry (APCI-MS) semiportable compact version (Advion): is one of less sensitive but more affordable versions of mass spectrometers released to the commercial market in recent years. The device uses APCI to produce ions. Although the most common use of APCI-MS systems is the detection in liquid chromatography applications,

For peer review only - http://bmjopen.bmj.com/site/about/guidelines.xhtml

the technique has proven to be a valuable tool for direct measurement of VOC in air[74, 75] food[76, 77] and breath[78, 79]. Recently, the technique has shown potential for online, real time profiling of pseudo-metabolites in exhaled breath [80] with sensitivity comparable with other techniques. By combining miniaturised MS technology with APCI techniques, adequate quality of on-site, real time measurements with minimal or no sample preparation requirement can be provided. This is a desirable outcome as it overcomes main limitation of using standard breath analysis method in clinical setting, which is a need for breath sample collection followed by desorption and time-consuming laboratory [illegible]

There remains an overall lack of standardisation and rigour across these technologies which hindered previous advancements in breath discovery; something we intend to minimize.

The instrument used will be an Advion Compact Mass Spectrometer Express, with atmospheric pressure chemical ionisation, interfaced with a heated breath sampling line (Advion, New York, USA).

4. Chemometric processing and data analysis:

GC-MS breath data will be aligned, deconvoluted and the features for each participant will be extracted. The extracted features will be grouped and classified by retention index and mass spectrum. The registered and aligned data will be linked to participant meta-data to generate a breath matrix. Data handling and analysis will be performed by a senior statistician.

The breath matrix is a $n \times p$ matrix where n is the number of subjects and p is the number of VOC. The breath matrix is high dimensional with $p \gg n$ and many potentially correlated VOC. In view of this, we will employ sparse partial least squares discriminant analysis (sPLS-DA)[81] to investigate which of the VOC can identify breathlessness. We will also investigate which of the VOC can discriminate between the different disease states including acute exacerbations of asthma and COPD and

For peer review only

Pneumonia. In addition to the supervised methods, unsupervised methods will be explored, specifically sparse principle component analysis (sPCA)[82].

Extracted VOC will also be investigated. Relationships between VOC and patient reported acute breathlessness will be analysed using logistic regression model. VOC associated with patient associated acute breathlessness will be incorporated into multinomial logistic regression models in conjunction with CRP, BNP, blood eosinophils and Troponin-I, pathology biomarkers currently in use for diagnosing undifferentiated breathlessness. In addition to the conventional binary and multinomial logistic regression models, regression models [83]..

5. Ethics and dissemination:

The study has obtained full ethical approval from the London South East Research ethics Committee, REC reference 16/LO/1747. IRAS project ID 198921. Publications will be prepared according to the MRC-EMBER consortium agreement and the University of Leicester publications policy. All intended publications will be submitted to the EMBER executive board for review and comments within 60 days of journal submission. Authorship will be according to contribution and internationally recognised guidance on journal authorship.

6. Study dates: 01/2/2017 – 30/10/2020

7. Authors' contributions:

S.S, C.E.B, N.Gr, P.Th and P.Mo conceived the study, obtained funding, wrote the study protocol, obtained ethical and MHRA approvals for the study and coordinated the deployment of analytical testing methods for breath analysis. W.I took the lead in writing the manuscript with support from S.S. Planning and recruitment of adult participants was carried out by W.I, S.Jo, B.Pa, A.Aw, R.Ph, G.Fo, A.Yo, R. J. R and C.Wh. Paediatrics study design was conceived by E.Ga and C.Be and participants recruited by T. Mc and C. Fo. Analytical chemistry team formed of M.Wi, R.Co, D.Sa, D.Ru and L.Br expertly handled all the breath samples and planned an analysis structure. M.Ri, a

For peer review only - http://bmjopen.bmj.com/site/about/guidelines.xhtml

senior statistician, constructed a statistics and data analysis plan in conjunction with SS. Bioinformatics pipeline and electronic CRFs developed by R.Fr and B.Zh. All authors, including R.Pe, H. Bh, B.Ha, A. Si, K. Ry, H. Pa, T. Su, L. L Ng, T. Co contributed to the study design and study protocol.

8. Protocol version: Version 4, 1st April 2018

9. Public and patient involvement:

A series of consultations have taken place with our patient involvement team within the NIHR Biomedical Research Centre (Respiratory Theme) and across the wider BRC PPI group. Representations from the paediatrics team were also present. This group was sent copies of the participant documentation for review and discussion. Various revisions have been made following on from these discussions.

10. Funding statement:

Research funding was provided by the MRC and EPSRC Stratified Medicine Grant for Molecular Pathology Nodes (Grant No. MR/N005880/1). The research was supported by the National Institute for Health Research (NIHR) Leicester Biomedical Research Centre and NIHR, Leicester Clinical Research Facility and the Midlands Asthma and Allergy Research Association (MAARA) to whom we are extremely grateful. The authors would like to acknowledge the invaluable efforts of the research nurses responsible for the in-clinic sample collection as well as the input from the wider EMBER consortium (Members list can be found at: https://ember.le.ac.uk/web). The views expressed are those of the author(s) and not necessarily those of the NHS and NIHR or the Department of Health

11. Competing interests: SS has performed advisory services for Owlstone Medical.

For peer review only - http://bmjopen.bmj.com/site/about/guidelines.xhtml

Figure legends:

Figure (1) Relationship between lung proximity and degree of invasiveness of different lung matrices. The figure plots the level of invasiveness of various lung matrices in relation to their proximity to the lung. Given their pathological relevance, the degree of invasiveness of bronchoalveolar lavage (BAL) and lung biopsy makes them less favourable in diagnosing respiratory diseases.

Figure (2) Multi-instrument use in breath sampling. Figure illustrates the various combinations of offline and online devices used in breath sampling and the relevant pros and cons. Offline and online technologies are used for the discovery and validation phases of the study respectively.

Figure (3) Study flow chart. Figure outlines the patient journey from admission through to discharge and follow up. Participants with self-reported acute breathlessness presenting to University Hospitals of Leicester are recruited within 24 hours following a senior decision maker review. Breath sampling is carried out on the first visit, at recruitment, and the second visit, up to 6 months after discharge. Patients are admitted through the standard operational emergency medical streaming and care pathways at the University Hospitals of Leicester NHS Trust. Early outcomes (hospital readmission) are measured at 30 and 60 days and late outcomes (mortality) including respiratory and all-cause mortality are measured at 2 years. Assessments carried out at each time point are summarised in **Table 1**.

Figure (4) Multi-instrument use in breath sampling. Operational space of the analytical technologies used in EMBER for the analysis of volatile organic compounds (VOC) in exhaled breath, including proton transfer reaction-mass spectrometry (PTR-MS), atmospheric pressure chemical ionisation-mass spectrometry (CMS), gas chromatography-ion mobility spectrometry (GC-IMS), gas chromatography-mass spectrometry (GC-MS) and two-dimensional gas chromatography-mass spectrometry (GCxGC). Comparing the typical molar mass range detectable; selectivity in detection owing to the type of ionisation involved and the proton affinity of analytes; and the inclusion of a chromatographic separation affecting total time of analysis. The online technologies involving

For peer review only - http://bmjopen.bmj.com/site/about/guidelines.xhtml

chemical ionisation (PTR-MS, CMS and GC-IMS) can be used in-clinic owing to short analysis times, but only detect lower molar mass molecules with a proton affinity higher than 697 KJ mol-1. Offline chromatographic techniques (GC-MS and GCxGC) detect a wider range of compounds independent of proton affinity; however, the techniques have longer analysis times and involve sample transportation and storage.

For peer review only

For peer review only - http://bmjopen.bmj.com/site/about/guidelines.xhtml

8. References

1. Stephenson, J.J., et al., *Clinical and economic burden of dyspnea and other COPD symptoms in a managed care setting.* Int J Chron Obstruct Pulmon Dis, 2017. **12**: p. 1947-1959.
2. David Ponka, M.K., *Top differential diagnoses in family medicine Dyspnoea Can Fam Physician.* Canadian Family Physician, 2008.
3. Figarska, S.M., H.M. Boezen, and J.M. Vonk, *Dyspnea severity, changes in dyspnea status and mortality in the general population: the Vlagtwedde/Vlaardingen study.* European Journal of Epidemiology, 2012. **27**(11): p. 867-876.
4. Parshall, M.B., et al., *An Official American Thoracic Society Statement: Update on the Mechanisms, Assessment, and Management of Dyspnea.* American Journal of Respiratory and Critical Care Medicine, 2012. **185**(4): p. 435-452.
5. Lim, W.S., et al., *BTS guidelines for the management of community acquired pneumonia in adults: update 2009.* Thorax, 2009. **64 Suppl 3**: p. iii1-55.
6. Leuzzi, G., et al., *C-reactive protein level predicts mortality in COPD: a systematic review and meta-analysis.* Eur Respir Rev, 2017. **26**(143).
7. Moe, G.W., *B-type natriuretic peptide in heart failure.* Curr Opin Cardiol, 2006. **21**(3): p. 208-14.
8. Ponikowski, P., et al., *2016 ESC Guidelines for the diagnosis and treatment of acute and chronic heart failure: The Task Force for the diagnosis and treatment of acute and chronic heart failure of the European Society of Cardiology (ESC)Developed with the special contribution of the Heart Failure Association (HFA) of the ESC.* Eur Heart J, 2016. **37**(27): p. 2129-2200.
9. Alex Sakula, *Charcot leyden crystals and crushmann spirals.* Thorax, 1986.
10. Calhoun, W.J., J. Sedgwick, and W.W. Busse, *The role of eosinophils in the pathophysiology of asthma.* Ann N Y Acad Sci, 1991. **629**: p. 62-72.
11. Pavord, I.D., *Eosinophilic phenotypes of airway disease.* Ann Am Thorac Soc, 2013. **10 Suppl**: p. S143-9.
12. Tashkin, D.P. and M.E. Wechsler, *Role of eosinophils in airway inflammation of chronic obstructive pulmonary disease.* International Journal of Chronic Obstructive Pulmonary Disease, 2018. **13**: p. 335-349.
13. Bafadhel, M., et al., *Blood Eosinophils to Direct Corticosteroid Treatment of Exacerbations of Chronic Obstructive Pulmonary Disease: A Randomized Placebo-Controlled Trial.* American Journal of Respiratory and Critical Care Medicine, 2012. **186**(1): p. 48-55.
14. Bedossa, P. and K. Patel, *Biopsy and Noninvasive Methods to Assess Progression of Nonalcoholic Fatty Liver Disease.* Gastroenterology, 2016. **150**(8): p. 1811-1822 e4.
15. Fens, N., et al., *Exhaled breath analysis by electronic nose in airways disease. Established issues and key questions.* Clin Exp Allergy, 2013. **43**(7): p. 705-15.
16. Probert, C.S., et al., *Volatile organic compounds as diagnostic biomarkers in gastrointestinal and liver diseases.* J Gastrointestin Liver Dis, 2009. **18**(3): p. 337-43.
17. Schubert, J.K., et al., *Breath analysis in critically ill patients: potential and limitations.* Expert Rev Mol Diagn, 2004. **4**(5): p. 619-29.
18. Fowler, S.J., et al., *Surveillance for lower airway pathogens in mechanically ventilated patients by metabolomic analysis of exhaled breath: a case-control study.* Thorax, 2015. **70**(4): p. 320-5.
19. Florian Gahleitner, C.G.-H., Caroline S Beardsmore, Hitesh C Pandya, CL Paul Thomas, *Metabolomics pilot study to identify VOC of childhood asthma in exhaled breath.* Bioanalysis, 2013.
20. Van Berkel, J.J., et al., *A profile of volatile organic compounds in breath discriminates COPD patients from controls.* Respir Med, 2010. **104**(4): p. 557-63.

For peer review only - http://bmjopen.bmj.com/site/about/guidelines.xhtml

21. Basanta, M., et al., *Exhaled volatile organic compounds for phenotyping chronic obstructive pulmonary disease: a cross-sectional study.* Respir Res, 2012. **13**: p. 72.
22. Besa, V., et al., *Exhaled volatile organic compounds discriminate patients with chronic obstructive pulmonary disease from healthy subjects.* International Journal of Chronic Obstructive Pulmonary Disease, 2015. **10**: p. 399-406.
23. Hauschild, A.C., J.I. Baumbach, and J. Baumbach, *Integrated statistical learning of metabolic ion mobility spectrometry profiles for pulmonary disease identification.* Genet Mol Res, 2012. **11**(3): p. 2733-44.
24. Jareno-Esteban, J.J., et al., *Study of 5 Volatile Organic Compounds in Exhaled Breath in Chronic Obstructive Pulmonary Disease.* Archivos De Bronconeumologia, 2017. **53**(5): p. 251-256.
25. Paredi, P., et al., *Exhaled ethane, a marker of lipid peroxidation, is elevated in chronic obstructive pulmonary disease.* Am J Respir Crit Care Med, 2000. **162**(2 Pt 1): p. 369-73.
26. Caldeira, M., et al., *Profiling allergic asthma volatile metabolic patterns using a headspace-solid phase microextraction/gas chromatography based methodology.* J Chromatogr A, 2011. **1218**(24): p. 3771-80.
27. Caldeira, M., et al., *Allergic asthma exhaled breath metabolome: a challenge for comprehensive two-dimensional gas chromatography.* J Chromatogr A, 2012. **1254**: p. 87-97.
28. Dallinga, J.W., et al., *Volatile organic compounds in exhaled breath as a diagnostic tool for asthma in children.* Clin Exp Allergy, 2010. **40**(1): p. 68-76.
29. Dragonieri, S., et al., *An electronic nose in the discrimination of patients with asthma and controls.* J Allergy Clin Immunol, 2007. **120**(4): p. 856-62.
30. Fens, N., et al., *Exhaled breath profiling enables discrimination of chronic obstructive pulmonary disease and asthma.* Am J Respir Crit Care Med, 2009. **180**(11): p. 1076-82.
31. Ibrahim, B., et al., *Non-invasive phenotyping using exhaled volatile organic compounds in asthma.* Thorax, 2011. **66**(9): p. 804-9.
32. Montuschi, P., et al., *Diagnostic performance of an electronic nose, fractional exhaled nitric oxide, and lung function testing in asthma.* Chest, 2010. **137**(4): p. 790-6.
33. Christiansen, A., et al., *A systematic review of breath analysis and detection of volatile organic compounds in COPD.* J Breath Res, 2016. **10**(3): p. 034002.
34. de Vries, R., et al., *Integration of electronic nose technology with spirometry: validation of a new approach for exhaled breath analysis.* J Breath Res, 2015. **9**(4): p. 046001.
35. Shafiek, H., et al., *Using the Electronic Nose to Identify Airway Infection during COPD Exacerbations.* PLoS One, 2015. **10**(9): p. e0135199.
36. Finamore, P., et al., *Analysis of volatile organic compounds: an innovative approach to heart failure characterization in older patients.* J Breath Res, 2018. **12**(2): p. 026007.
37. *MRC Molecular Pathology, Review 20 Aug.*
38. Medical Research Council, *Molecular Pathology Nodes*. 2014: MRC website.
39. Corradi, M., et al., *Aldehydes in exhaled breath condensate of patients with chronic obstructive pulmonary disease.* Am J Respir Crit Care Med, 2003. **167**(10): p. 1380-6.
40. Larstad, M., et al., *Determination of malondialdehyde in breath condensate by high-performance liquid chromatography with fluorescence detection.* J Chromatogr B Analyt Technol Biomed Life Sci, 2002. **766**(1): p. 107-14.
41. F. Y. Hsieh, D.A.B., Michael D. Larsen,, *A simple method of sample size calculation for linear and logistic regression* Statis Med, 1998.
42. Hajian-Tilaki, K., *Sample size estimation in diagnostic test studies of biomedical informatics.* J Biomed Inform, 2014. **48**: p. 193-204.
43. Quan, H., et al., *Updating and validating the Charlson comorbidity index and score for risk adjustment in hospital discharge abstracts using data from 6 countries.* Am J Epidemiol, 2011. **173**(6): p. 676-82.

For peer review only - http://bmjopen.bmj.com/site/about/guidelines.xhtml

855 44. Roffman, C.E., J. Buchanan, and G.T. Allison, *Charlson Comorbidities Index.* J Physiother, 2016. **62**(3): p. 171.
857 45. Bjarnason, H. and P. Hougaard, *Fisher information for two gamma frailty bivariate Weibull models.* Lifetime Data Anal, 2000. **6**(1): p. 59-71.
859 46. Lawrence Gould, A., et al., *Joint modeling of survival and longitudinal non-survival data: current methods and issues. Report of the DIA Bayesian joint modeling working group.* Stat Med, 2015. **34**(14): p. 2181-95.
862 47. Steer, J., et al., *Dyspnoea severity and pneumonia as predictors of in-hospital mortality and early readmission in acute exacerbations of COPD.* Thorax, 2012. **67**(2): p. 117-21.
864 48. Tsimogianni, A.M., et al., *Predictors of Outcome After Exacerbation of Chronic Obstructive Pulmonary Disease.* Journal of General Internal Medicine, 2009. **24**(9): p. 1043-1048.
866 49. Kitchen, S., et al., *LATE-BREAKING ABSTRACT: Breathe free: Open source development of a breath sampler by a consortium of breath researchers.* European Respiratory Journal, 2015. **46**(suppl 59).
869 50. Oostveen, E., et al., *The forced oscillation technique in clinical practice: methodology, recommendations and future developments.* Eur Respir J, 2003. **22**(6): p. 1026-41.
871 51. Pendharkar, S. and S. Mehta, *The clinical significance of exhaled nitric oxide in asthma.* Canadian Respiratory Journal : Journal of the Canadian Thoracic Society, 2008. **15**(2): p. 99-106.
874 52. Yates, D.H., *Role of exhaled nitric oxide in asthma.* Immunol Cell Biol, 2001. **79**(2): p. 178-90.
875 53. Lang, R.M., et al., *Recommendations for chamber quantification: a report from the American Society of Echocardiography's Guidelines and Standards Committee and the Chamber Quantification Writing Group, developed in conjunction with the European Association of Echocardiography, a branch of the European Society of Cardiology.* J Am Soc Echocardiogr, 2005. **18**(12): p. 1440-63.
880 54. J. Throck Watson, O.D.S., *Introduction to Mass Spectrometry: Instrumentation, Applications, and Strategies for Data Interpretation, 4th Edition*. 2007: Wiley. 862.
882 55. Buszewski, B., et al., *Human exhaled air analytics: biomarkers of diseases.* Biomed Chromatogr, 2007. **21**(6): p. 553-66.
884 56. Amann, A., et al., *Applications of breath gas analysis in medicine.* International Journal of Mass Spectrometry, 2004. **239**(2-3): p. 227-233.
886 57. Almstetter, M.F., P.J. Oefner, and K. Dettmer, *Comprehensive two-dimensional gas chromatography in metabolomics.* Anal Bioanal Chem, 2012. **402**(6): p. 1993-2013.
888 58. Das, M.K., et al., *Investigation of Gender-Specific Exhaled Breath Volatome in Humans by GCxGC-TOF-MS.* Analytical Chemistry, 2014. **86**(2): p. 1229-1237.
890 59. Phillips, M., et al., *Detection of an extended human volatome with comprehensive two-dimensional gas chromatography time-of-flight mass spectrometry.* PLoS One, 2013. **8**(9): p. e75274.
893 60. Gruber, B., et al., *Vacuum ultraviolet absorption spectroscopy in combination with comprehensive two-dimensional gas chromatography for the monitoring of volatile organic compounds in breath gas: A feasibility study.* J Chromatogr A, 2016. **1464**: p. 141-6.
896 61. Gruber, B., et al., *Breath gas monitoring during a glucose challenge by a combined PTR-QMS/GCxGC-TOFMS approach for the verification of potential volatile biomarkers.* J Breath Res, 2016. **10**(3): p. 036003.
899 62. Mellors, T.R., et al., *A new method to evaluate macaque health using exhaled breath: A case study of M. tuberculosis in a BSL-3 setting.* J Appl Physiol (1985), 2017. **122**(3): p. 695-701.
901 63. Phillips, M., et al., *Breath biomarkers of whole-body gamma irradiation in the Gottingen minipig.* Health Phys, 2015. **108**(5): p. 538-46.
903 64. Bajtarevic, A., et al., *Noninvasive detection of lung cancer by analysis of exhaled breath.* BMC Cancer, 2009. **9**: p. 348.

For peer review only - http://bmjopen.bmj.com/site/about/guidelines.xhtml

65. Lavra, L., et al., *Investigation of VOCs associated with different characteristics of breast cancer cells.* Sci Rep, 2015. **5**: p. 13246.
66. Zhou, W., et al., *Exhaled breath online measurement for cervical cancer patients and healthy subjects by proton transfer reaction mass spectrometry.* Anal Bioanal Chem, 2017. **409**(23): p. 5603-5612.
67. Fernández del Río, R., et al., *Volatile Biomarkers in Breath Associated With Liver Cirrhosis — Comparisons of Pre- and Post-liver Transplant Breath Samples.* EBioMedicine, 2015. **2**(9): p. 1243-1250.
68. O'Hara, M.E., et al., *Limonene in exhaled breath is elevated in hepatic encephalopathy.* J Breath Res, 2016. **10**(4): p. 046010.
69. Tomasz Ligor, C.A., Anton Amann, Konrad Schwarz, Bogusaw Buszewski, Wojciech ebrowski,, *<Comparison of Proton Transfer Reaction-Mass Spectrometry and Gas Chromatography-Mass Spectrometry in Analysis of Breath Samples.pdf>.* Chem. Anal., 2009.
70. Darwiche, K., et al., *Bronchoscopically obtained volatile biomarkers in lung cancer.* Lung, 2011. **189**(6): p. 445-52.
71. Handa, H., et al., *Exhaled Breath Analysis for Lung Cancer Detection Using Ion Mobility Spectrometry.* PLoS ONE, 2014. **9**(12): p. e114555.
72. Bessa, V., et al., *Detection of volatile organic compounds (VOCs) in exhaled breath of patients with chronic obstructive pulmonary disease (COPD) by ion mobility spectrometry.* International Journal for Ion Mobility Spectrometry, 2011. **14**(1): p. 7-13.
73. Schivo, M., et al., *Mobile instrumentation platform to distinguish airway disorders.* Journal of breath research, 2013. **7**(1): p. 017113-017113.
74. Badjagbo, K., et al., *Direct atmospheric pressure chemical ionization-tandem mass spectrometry for the continuous real-time trace analysis of benzene, toluene, ethylbenzene, and xylenes in ambient air.* J Am Soc Mass Spectrom, 2009. **20**(5): p. 829-36.
75. Healy, R.M., et al., *A multi-year study of VOC emissions at a chemical waste disposal facility using mobile APCI-MS and LPCI-MS instruments.* Environ Pollut, 2018. **232**: p. 220-228.
76. Linforth, R. and A.J. Taylor, *Persistence of Volatile Compounds in the Breath after Their Consumption in Aqueous Solutions.* Journal of Agricultural and Food Chemistry, 2000. **48**(11): p. 5419-5423.
77. Perez-Hurtado, P., et al., *Direct analysis of volatile organic compounds in foods by headspace extraction atmospheric pressure chemical ionisation mass spectrometry.* Rapid Commun Mass Spectrom, 2017. **31**(22): p. 1947-1956.
78. Berchtold, C., L. Meier, and R. Zenobi, *Evaluation of extractive electrospray ionization and atmospheric pressure chemical ionization for the detection of narcotics in breath.* International Journal of Mass Spectrometry, 2011. **299**(2-3): p. 145-150.
79. Kapishon, V., et al., *Atmospheric pressure chemical ionization mass spectrometry of pyridine and isoprene: potential breath exposure and disease biomarkers.* J Breath Res, 2013. **7**(2): p. 026005.
80. Heaney, L.M., et al., *Real-time monitoring of exhaled volatiles using atmospheric pressure chemical ionization on a compact mass spectrometer.* Bioanalysis, 2016. **8**(13): p. 1325-36.
81. Lê Cao, K.-A., S. Boitard, and P. Besse, *Sparse PLS discriminant analysis: biologically relevant feature selection and graphical displays for multiclass problems.* BMC Bioinformatics, 2011. **12**: p. 253-253.
82. Zou, H., T. Hastie, and R. Tibshirani, *Sparse Principal Component Analysis.* Journal of Computational and Graphical Statistics, 2006. **15**(2): p. 265-286.
83. Zou H, H.T., *Regularization and Variable Selection via the Elastic Net.* Journal of the Royal Statistical Society, 2005.

For peer review only

For peer review only - http://bmjopen.bmj.com/site/about/guidelines.xhtml

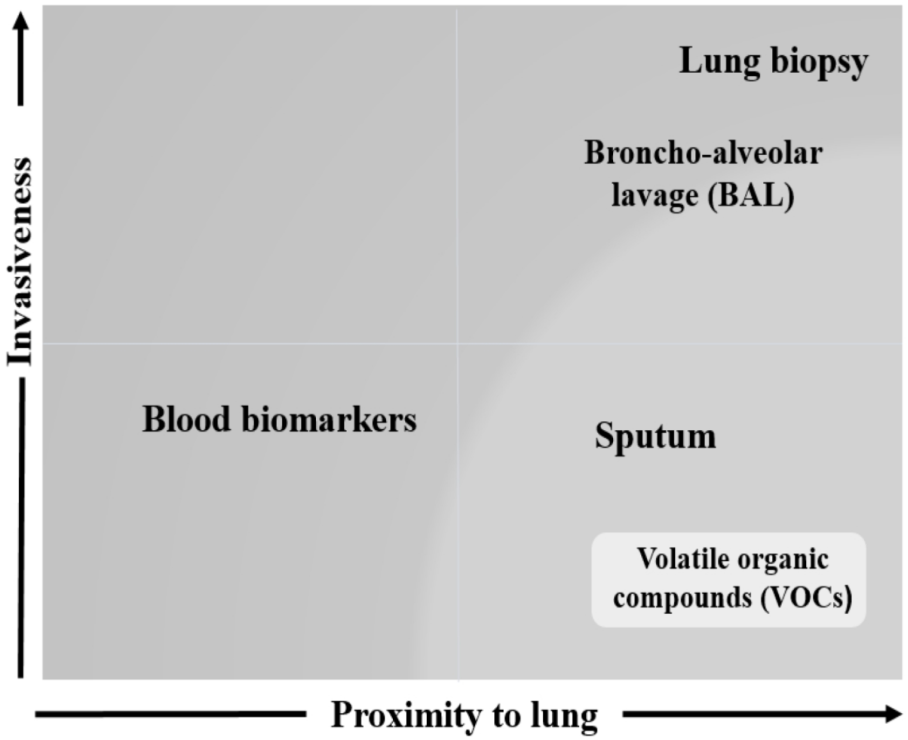

Figure (1): Relationship between lung proximity and degree of invasiveness of different lung matrices. The figure plots the level of invasiveness of various lung matrices in relation to their proximity to the lung. Given their pathological relevance, the degree of invasiveness of bronchoalveolar lavage (BAL) and lung biopsy makes them less favourable in diagnosing respiratory diseases.

144x115mm (300 x 300 DPI)

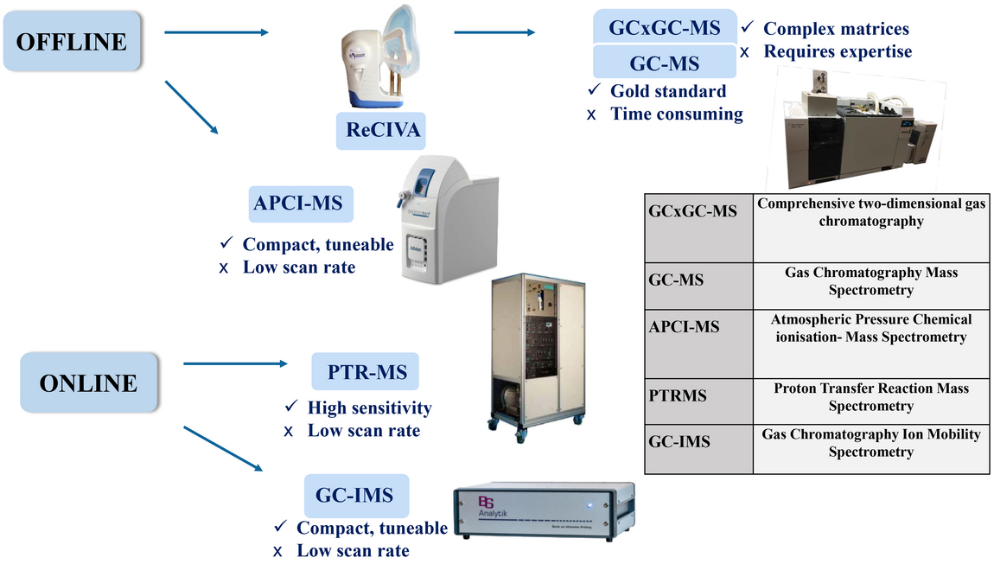

Figure (2): Multi-instrument use in breath sampling. Figure illustrates the various combinations of offline and online devices used in breath sampling and the relevant pros and cons. Offline and online technologies are used for the discovery and validation phases of the study respectively.

97x55mm (300 x 300 DPI)

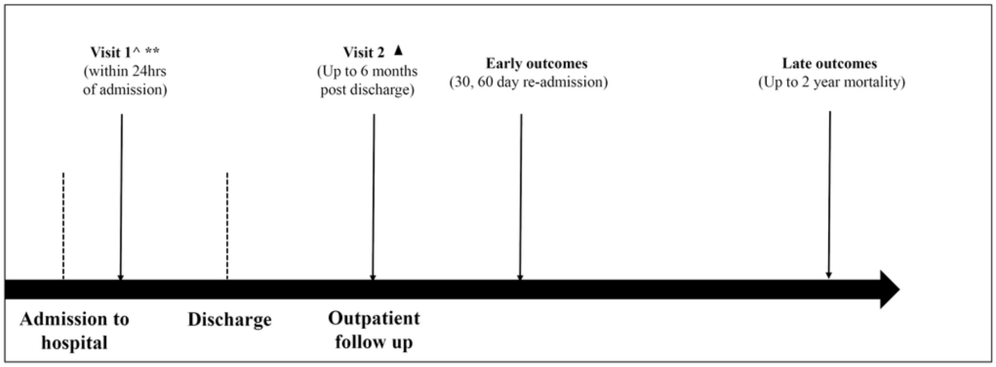

^ Following senior decision maker review
** Breath sampling (ReCIVA-GC-MS, ReCIVA- GCxGC-MS, GC-IMS, APCI-MS)
▲ Breath sampling (ReCIVA-GC-MS, ReCIVA- GCxGC-MS, PTR-MS, APCI-MS)
---- Following University Hospitals of Leicester streaming and clinical care pathways

Figure (3): Study flow chart. Figure outlines the patient journey from admission through to discharge and follow up. Participants with self-reported acute breathlessness presenting to University Hospitals of Leicester are recruited within 24 hours following a senior decision maker review. Breath sampling is carried out on the first visit, at recruitment, and the second visit, up to 6 months after discharge. Patients are admitted through the standard operational emergency medical streaming and care pathways at the University Hospitals of Leicester NHS Trust. Early outcomes (hospital readmission) are measured at 30 and 60 days and late outcomes (mortality) including respiratory and all-cause mortality are measured at 2 years. Assessments carried out at each time point are summarised in Table 1.

86x43mm (300 x 300 DPI)

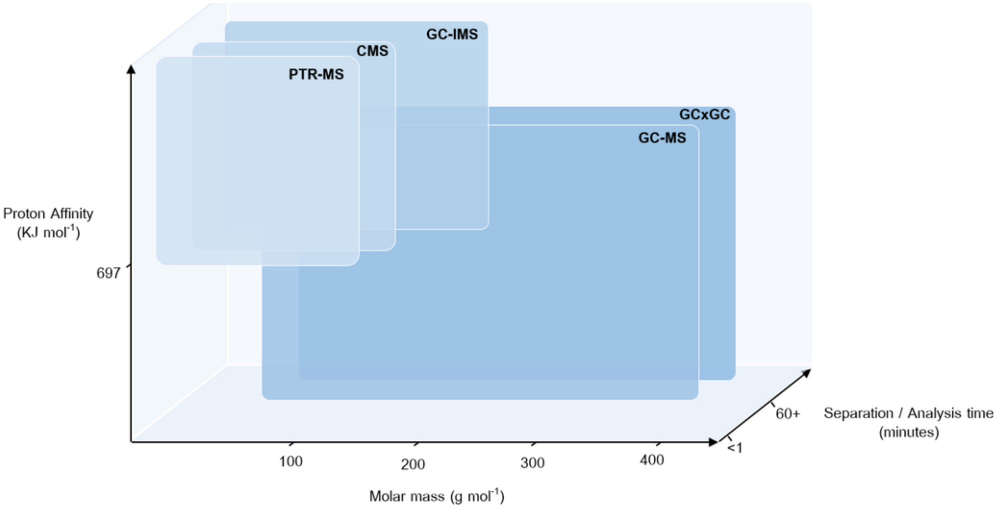

Figure (4): Multi-instrument use in breath sampling. Operational space of the analytical technologies used in EMBER for the analysis of volatile organic compounds (VOC) in exhaled breath, including proton transfer reaction-mass spectrometry (PTR-MS), atmospheric pressure chemical ionisation-mass spectrometry (CMS), gas chromatography-ion mobility spectrometry (GC-IMS), gas chromatography-mass spectrometry (GC-MS) and two-dimensional gas chromatography-mass spectrometry (GCxGC). Comparing the typical molar mass range detectable; selectivity in detection owing to the type of ionisation involved and the proton affinity of analytes; and the inclusion of a chromatographic separation affecting total time of analysis. The online technologies involving chemical ionisation (PTR-MS, CMS and GC-IMS) can be used in-clinic owing to short analysis times, but only detect lower molar mass molecules with a proton affinity higher than 697 KJ mol-1. Offline chromatographic techniques (GC-MS and GCxGC) detect a wider range of compounds independent of proton affinity; however, the techniques have longer analysis times and involve sample transportation and storage.

88x44mm (300 x 300 DPI)
